# Molecular Determinants for Recognition of Divergent SAMHD1 Proteins by the Lentiviral Accessory Protein Vpx

**DOI:** 10.1016/j.chom.2015.03.004

**Published:** 2015-04-08

**Authors:** David Schwefel, Virginie C. Boucherit, Evangelos Christodoulou, Philip A. Walker, Jonathan P. Stoye, Kate N. Bishop, Ian A. Taylor

**Affiliations:** 1 Division of Molecular Structure, MRC National Institute for Medical Research, The Ridgeway, Mill Hill, London NW7 1AA, UK; 2 Division of Virology, MRC National Institute for Medical Research, The Ridgeway, Mill Hill, London NW7 1AA, UK; 3 Faculty of Medicine, Imperial College London, London SW7 2AZ, UK

## Abstract

The SAMHD1 triphosphohydrolase inhibits HIV-1 infection of myeloid and resting T cells by depleting dNTPs. To overcome SAMHD1, HIV-2 and some SIVs encode either of two lineages of the accessory protein Vpx that bind the SAMHD1 N or C terminus and redirect the host cullin-4 ubiquitin ligase to target SAMHD1 for proteasomal degradation. We present the ternary complex of Vpx from SIV that infects mandrills (SIV_mnd-2_) with the cullin-4 substrate receptor, DCAF1, and N-terminal and SAM domains from mandrill SAMHD1. The structure reveals details of Vpx lineage-specific targeting of SAMHD1 N-terminal “degron” sequences. Comparison with Vpx from SIV that infects sooty mangabeys (SIV_smm_) complexed with SAMHD1-DCAF1 identifies molecular determinants directing Vpx lineages to N- or C-terminal SAMHD1 sequences. Inspection of the Vpx-DCAF1 interface also reveals conservation of Vpx with the evolutionally related HIV-1/SIV accessory protein Vpr. These data suggest a unified model for how Vpx and Vpr exploit DCAF1 to promote viral replication.

## Highlights

• The crystal structure of SIV_mnd-2_ Vpx complexed with SAMHD1 and DCAF1
• Comparisons to SIV_smm_ Vpx reveal determinants for N- versus C-terminal SAMHD1 binding
• Vpx and Vpr use conserved determinants to bind to DCAF1

## Graphical Abstract

**Figure:**
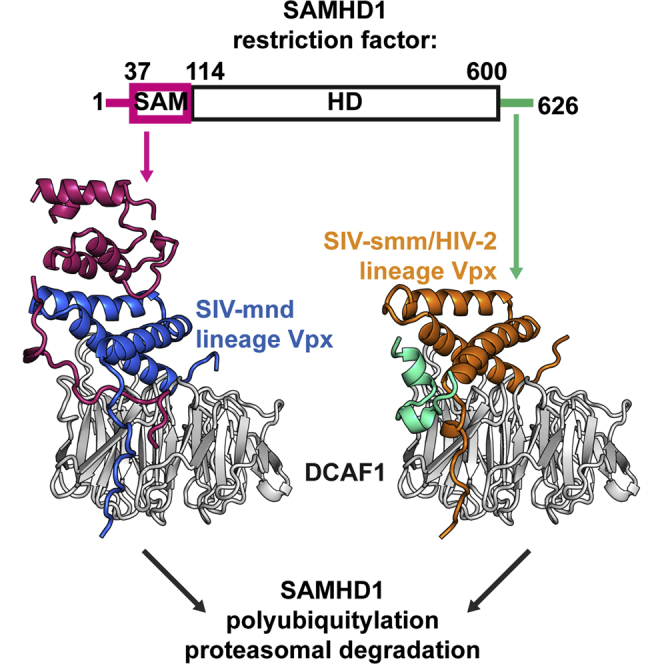

## Teaser

Vpx of HIV-2 and SIV target the restriction factor SAMHD1 for degradation using the host ubiquitin ligase substrate receptor DCAF1. Schwefel et al. present the ternary complex of Vpx_mnd-2_ with DCAF1 and the SAMHD1 N terminus and reveal how Vpx subverts host cell defenses by employing differing strategies to recruit SAMHD1 for degradation.

## Introduction

Host cell restriction factors are the first line of cellular defense against retrovirus infection. Successful viral evasion of these adaptive hurdles is essential for productive infection of new hosts and has occurred during multiple cross-species transmission events such as those that led to the emergence of pandemic human immunodeficiency virus-1 (HIV-1) (Hatziioannou and Bieniasz, 2011; Hatziioannou et al., 2014; Sharp and Hahn, 2011). Restriction factors are often interferon induced and inhibit distinct stages of the viral replication cycle (Towers and Noursadeghi, 2014; Wolf and Goff, 2008). Important examples are the TRIM5α, APOBEC3 family, and Tetherin proteins, which interfere with retroviral uncoating, reverse transcription, and budding processes, respectively (Neil et al., 2008; Sheehy et al., 2002; Stremlau et al., 2004). The sterile alpha motif domain and histidine-aspartate domain-containing protein 1 (SAMHD1) was first identified as a disease gene associated with the rare infantile encephalopathy Aicardi-Goutières syndrome (Rice et al., 2009). More recently, SAMHD1 was shown to be a factor that restricts HIV-1 replication in non-dividing myeloid-lineage cells and resting T cells (Baldauf et al., 2012; Berger et al., 2011; Descours et al., 2012; Hrecka et al., 2011; Laguette et al., 2011). SAMHD1 is a dGTP/GTP-activated deoxynucleotide (dNTP) triphosphohydrolase (Amie et al., 2013; Goldstone et al., 2011; Ji et al., 2013; Miazzi et al., 2014; Powell et al., 2011; Zhu et al., 2013) involved in balancing cellular dNTP pools (Franzolin et al., 2013) and regulated by Cyclin A2/CDK1-dependent phosphorylation (Cribier et al., 2013; Kretschmer et al., 2015; Pauls et al., 2014; White et al., 2013). It is proposed that SAMHD1 restriction results from this triphosphohydrolase activity by reducing the cellular dNTP concentration to a level insufficient for the viral reverse transcriptase to function (Kim et al., 2012; Lahouassa et al., 2012; Rehwinkel et al., 2013; St Gelais et al., 2012). An alternative mechanism of SAMHD1 HIV-1 restriction requiring a putative nuclease activity as also been reported, but the nature of the polynucleotide substrate is disputed (Beloglazova et al., 2013; Ryoo et al., 2014; Tüngler et al., 2013).

A hallmark of most retrovirus restriction factors is the existence of viral antagonists in the form of accessory proteins (Malim and Bieniasz, 2012; Strebel, 2013). A common mechanism of action of accessory proteins is the subversion of host cell protein degradation pathways (Strebel, 2013). In particular, the host cell’s Cullin-RING-type E3 ubiquitin ligases are often engaged by viral accessory proteins to induce restriction factor poly-ubiquitylation to direct proteasomal degradation (Barry and Früh, 2006). Cullin-RING ubiquitin ligases consist of a central Cullin scaffold protein, a catalytic RING subunit, and varying substrate receptors (Zimmerman et al., 2010). Owing to their modular architecture, Cullin-RING ligases allow for specific placement of a large number of substrates in the ubiquitylation zone of the catalytic subunit for efficient poly-ubiquitylation (Fischer et al., 2011; Zimmerman et al., 2010). Simian immunodeficiency virus (SIV) and HIV accessory proteins exploit these characteristics by modifying the specificity of Cullin-RING substrate receptors (e.g., HIV-1 Vif and Vpu redirect cullin-1 and cullin-5 receptor specificity to induce APOBEC3 and Tetherin restriction factor downregulation, respectively) (Guo et al., 2014; Mitchell et al., 2009). Vpr and Vpx from HIV and SIVs target the cullin-4 ligase substrate receptor DDB1- and CUL4-associated factor 1 (DCAF1, also known as VprBP) from their host (Bergamaschi et al., 2009; Hrecka et al., 2007; Le Rouzic et al., 2007; Srivastava et al., 2008).

Restriction factors and accessory proteins are engaged in an evolutionary “molecular arms race” consisting of multiple rounds of host adaptation, virus counteraction, and host re-adaptation, resulting in accumulation of amino acid changes in restriction factor-accessory protein interaction interfaces (Daugherty and Malik, 2012). In the lentiviral Vpx/Vpr accessory proteins, analyses of positively selected residues and subsequent functional studies have demonstrated the occurrence of several significant specificity changes during adaptation to primate hosts (Laguette et al., 2012; Lim et al., 2012). It is proposed that originally a subset of Vpr proteins acquired the capability to induce cullin-4/DCAF1-dependent proteasomal degradation of SAMHD1. These SAMHD1-degrading Vpr proteins are found in the SIV_syk_ (SIV that infects Sykes’ monkey), SIV_deb_ (De Brazza’s monkey), and SIV_agm_ (African green monkey) lineages. Later in its evolutionary history, a Vpr gene duplication or recombination event gave rise to Vpx, which completely took over the anti-SAMHD1 functionality and produced viruses that contain both Vpx and Vpr genes (Lim et al., 2012). In these adaptation events, the mode of SAMHD1 binding seems to have toggled between recognition sequences at the SAMHD1 N terminus and others located at the C terminus. Vpx from HIV-2, SIV_smm_ (sooty mangabey) and SIV_mac_ (macaque) all target the SAMHD1 C terminus, whereas SIV_mnd-2_ (mandrill) and SIV_rcm_ (red-capped mangabey) Vpx induce SAMHD1 degradation dependent on the N-terminal 110 residues. The SAMHD1-degrading Vpr proteins from SIV_syk_, SIV_deb_, and SIV_agm_ have varying specificities for the SAMHD1 N or C terminus or even target both (Fregoso et al., 2013; Spragg and Emerman, 2013).

Here, we report the crystal structure of the ternary complex of SIV_mnd-2_ Vpx bound to the N-terminal region and SAM domain of mandrill SAMHD1 (SAMHD1_mnd_-NtD) and the WD40 domain of the cullin-4 substrate receptor DCAF1 (DCAF1-CtD). This structure reveals unanticipated complexity in Vpx-mediated targeting of SAMHD1. Comparison to the recently determined structure of a SIV_smm_ Vpx/SAMHD1-CtD/DCAF1-CtD (Schwefel et al., 2014) complex provides the molecular details of the structural determinants for N-terminal versus C-terminal SAMHD1 specificity and a plausible scenario for the events occurring during evolutionary change.

## Results

### Identifying the Mandrill SAMHD1 Degron

**Figure 1:**
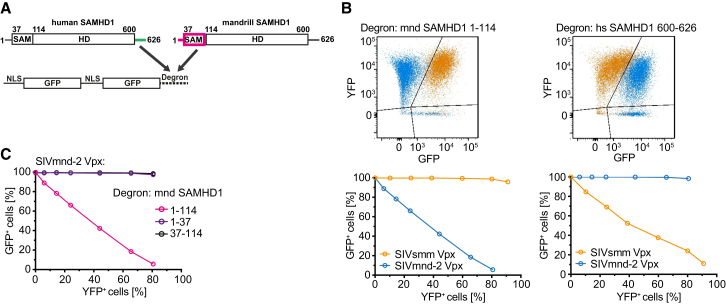
The Mandrill SAMHD1 Degron Comprises the N-Terminal and SAM Domains (A) Schematic of the human and mandrill SAMHD1 and the tandem NLS-EGFP degron fusion protein construct employed in fluorescent degron assays. The C- and N-terminal degron regions in SAMHD1 are highlighted in green and magenta, respectively. (B) Stable cell lines expressing either the mandrill (mnd) SAMHD1 1–114 degron (left) or human (hs) SAMHD1 600–626 degron (right) were transduced with increasing titers of particles carrying either SIV_smm_ Vpx (orange) or SIV_mnd-2_ Vpx (blue) together with YFP. The level of degron (EGFP) and Vpx (YFP) expression was measured by flow cytometry. The upper panels are example FACS plots illustrating the populations of cells observed: untransduced cells (bottom right quadrant), cells transduced with a Vpx protein that can (top left quadrant) or cannot (top right quadrant) induce degradation of the degron reporter construct. The lower panels are quantification of degron reporter expression with increasing Vpx expression, demonstrating differential Vpx recognition of degron sequences. (C) Quantification of degron reporter expression in stable cells lines expressing constructs containing the indicated N-terminal regions of SAMHD1_mnd_ after transduction with increasing amounts of SIV_mnd-2_ Vpx; see also Figure S1.

To locate the Vpx-DCAF1 recognition sequence within the SAMHD1_mnd_ N terminus, a cell-based EGFP-degron reporter degradation assay was employed (Schwefel et al., 2014). Degron fusion proteins (Figure 1A) were constructed comprising two copies of EGFP with a nuclear localization signal (NLS) fused to either the N-terminal 114 residues of SAMHD1_mnd_ (SAMHD1_mnd_-NtD) or a control C-terminal region of human SAMHD1, residues 600–626, (SAMHD1_hs_-CtD), which is targeted for degradation by Vpx from SIV_smm_ (Schwefel et al., 2014). Stable cell lines expressing each reporter construct were produced and expression levels of degron fusion proteins were confirmed by western blot (Figure S1). Cells were then transduced with increasing amounts of a bicistronic IRES vector expressing Vpx from SIV_mnd-2_ or SIV_smm_ and YFP, and 48 hr later, the level of degron reporter (EGFP) and Vpx (YFP) expression was measured by flow cytometry. These data show that SIV_mnd-2_ Vpx induces degradation of the SAMHD1_mnd_-NtD reporter construct but not EGFP-SAMHD1_hs_-CtD (Figure 1B, left panel). In contrast, SIV_smm_ Vpx induces degradation of the SAMHD1_hs_-CtD reporter but not SAMHD1_mnd_-NtD (Figure 1B, right panel). To further delineate the SIV_mnd-2_ SAMHD1 binding determinants, reporter constructs containing residues 1–37 and 37–114 of SAMHD1_mnd_ were prepared. Residues 1–37 comprise an N-terminal disordered sequence containing a NLS and 37–114 constitutes the SAM domain (cp. PDB: 2E8O). However, neither fragment alone was sufficient to induce degradation of the reporter construct (Figure 1C), demonstrating that both the N-terminal NLS region and SAM domain are required for SIV_mnd-2_ Vpx/DCAF1-mediated degradation.

### Structure of the SIV_mnd-2_ Vpx/SAMHD_mnd_-NtD/DCAF1-CtD Ternary Complex

**Figure 2:**
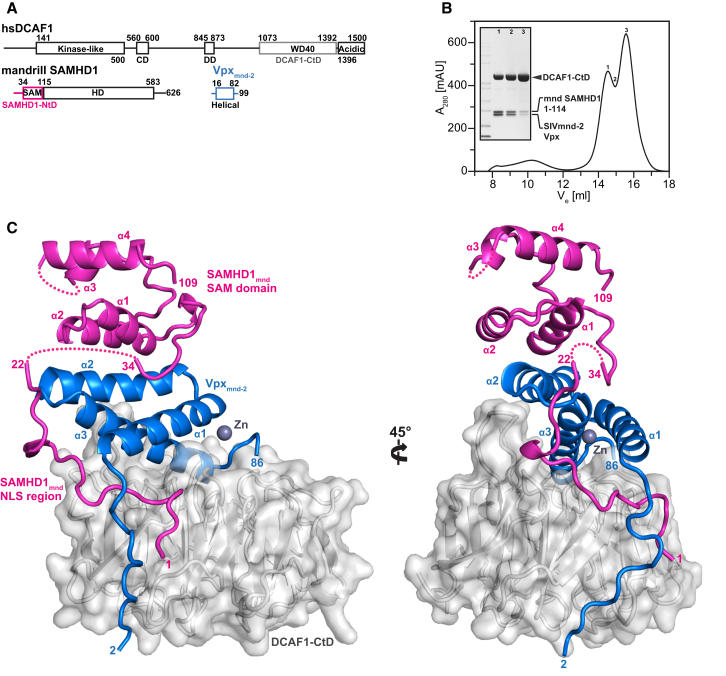
Structure of the SIV_mnd-2_ Vpx/DCAF1-CtD/SAMHD1_mnd_-NtD Complex (A) Schematic of components of the protein complex. (B) Analytical gel filtration of the in vitro-assembled ternary protein complex consisting of SIV_mnd-2_ Vpx, DCAF1-CtD and SAMHD1_mnd_-NtD (Peak-1). The inset shows SDS-PAGE analysis of the indicated peak fractions. (C) Structure of the ternary complex. The backbone for each protein is shown in cartoon representation, SIV_mnd-2_ Vpx (blue), DCAF1-CtD (gray), and SAMHD1_mnd_-NtD (magenta). See also Figure S2. The solvent-accessible surface is also shown for DCAF1-CtD, and a zinc ion co-ordinated by Vpx is displayed as a gray sphere, see also Figure S3. For SIV_mnd-2_ Vpx and SAMHD1_mnd_-NtD, residue numbers at chain termini are indicated and secondary structure elements labeled.

To gain further insights into molecular recognition in the SIV_mnd-2_ Vpx system, SIV_mnd-2_ Vpx, SAMHD1_mnd_-NtD, and DCAF1-CtD (Figure 2A) were assembled in vitro using recombinant proteins and the ternary complex purified by size-exclusion chromatography (Figure 2B). The protein complex crystallized in space group P6_5_22, the crystals diffracted to 2.8 Å resolution, and the structure was solved by molecular replacement using the SIV_smm_ Vpx/SAMHD1_hs_-CtD/DCAF1-CtD structure (Schwefel et al., 2014) as a search model. Details of the data collection, structure determination, map quality, and refinement are presented in Table 1 and Figure S2.

DCAF1-CtD consists of a typical WD40-repeat seven-bladed β-propeller fold approximately 45 Å diameter and 25 Å in height (Figure 2C). SIV_mnd-2_ Vpx comprises an N-terminal extended arm that precedes three α helices. The side chains of amino acid residues H35 at the C terminus of helix α1 together with H78 and C83 at the C terminus of helix α3 constitute a zinc-binding motif that links the helices together, similar to that observed in SIV_smm_ Vpx (Schwefel et al., 2014). However, in contrast the fourth zinc coordinating ligand in SIV_mnd-2_ Vpx is a water molecule rather than an additional cysteine C-terminal to C83 as occurs in SIV_smm_ Vpx (Figure S3). In the structure, two sections of the SAMHD1_mnd_-NtD are visible. Residues 1–22 are in an extended conformation, and residues 34-109 fold into the SAM domain, which consists of four α helices packed together by interaction of inward-facing hydrophobic amino acid side chains. The ternary complex is built up by three major protein interaction interfaces. These are (i) a Vpx-DCAF1 interface on top and side of the DCAF1 propeller, (ii) a combined interface between the N-terminal extended segment of SAMHD1-NtD, DCAF1 and the Vpx on the side of DCAF1, and (iii) contacts between the SAM domain and Vpx on top of DCAF1.

### The DCAF1 Binding Mode Is Conserved in Vpx Proteins

**Figure 3:**
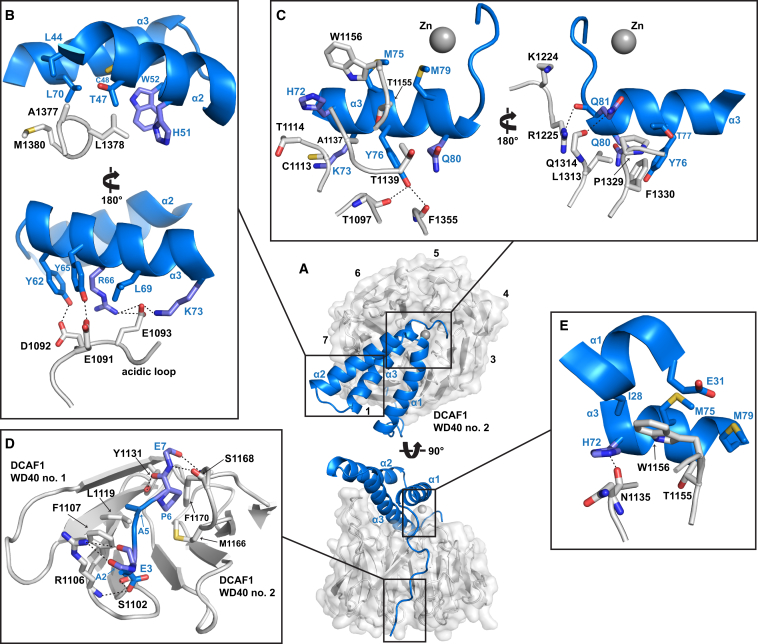
The SIV_mnd-2_ Vpx/DCAF1-CtD Interaction Interface (A) Overview of Vpx/DCAF1-CtD interactions. Molecules are in the same representation as Figure 2, the WD40 β-propeller repeats of DCAF1-CtD are numbered. (B–E) Details of Vpx/DCAF1-CtD interactions in the regions boxed in (A). Residues that make interactions are shown in stick representation, dashed lines indicate hydrogen bonds and salt bridges, see also Figure S4 and Table S1.

The extensive Vpx-DCAF1 interface comprises four contact regions with a total surface area of 1,600 Å^2^ (Figure 3; Table S1A). The first region comprises the C-terminal section of α2 together with first half of α3 of Vpx. On one face, apolar amino acid side chains from both α2 and α3 pack against a cluster of hydrophobic amino acids located in the “tall” loop that connects β2 to β3 in WD40 repeat 7 on the upper surface of DCAF1-CtD (Figure 3B, upper panel). On the other face, a network of hydrogen bonds and salt bridges connects Vpx residues Y62, Y65, R66, and K73 at the N terminus of α3 to acidic side chains E1191, D1092, and E1193 in the “acidic” loop of DCAF1 that intersperses WD40 repeats 7 and 1 (acidic loop, Figure 3B, lower panel). A second region of interaction involves the carboxy terminus of Vpx α3, which packs into a radial groove on the top side of DCAF1 lined by amino acids from WD40 repeats 1, 2, 4, 6, and 7. Interactions here include both the packing of hydrophobic residues as well as specific hydrogen bonding between the sidechains of Vpx Y76 and Q81 and the DCAF1 protein backbone (Figure 3C). The third binding interface involves residues at the extreme amino terminal of the Vpx N-terminal extended arm that inserts into a cavity between WD40 repeats 1 and 2 on the underside of DCAF1. Here, Vpx A2, E3 and E7 make main chain and side chain hydrogen-bonds to DCAF1 residues S1102, R1106, Y1131, and S1168 while the interposing Vpx residues A5 and P6 project into the hydrophobic cavity (Figure 3D). The remaining site of interaction involves contacts between residues in the WD40 repeat 2 of DCAF1 and residues in both Vpx α1 and α3. These include hydrophobic interactions between DCAF1 T1155 and W1156 and apolar side chains displayed on the underside of Vpx α1 and α3 and Vpx H72 on α3 that makes a hydrogen bond with mainchain carbonyl of N1135 of DCAF1 (Figure 3E).

Structural alignment of the DCAF1-CtD and Vpx molecules in the SIV_mnd-2_ and SIV_smm_ complexes (Schwefel et al., 2014) reveals that in both instances they adopt very similar conformations. The root-mean-square deviations (RMSDs) are 0.65 Å (305 aligned residues) for the DCAF1-CtD molecules and 1.24 Å for Vpx (80 aligned residues) (Figure S4A). Moreover, detailed comparison of SIV_mnd-2_ and SIV_smm_ Vpx amino acid side chains that contact DCAF1 shows that nearly all are at least type-conserved (Figures S4B–S4E). A notable exception is T47 in α2 that is exchanged to arginine in SIV_smm_ Vpx and is concomitant with a small movement of α2 and differences in conformation of the interacting DCAF1 tall loop between the two complexes (Figure S4B, upper panel). However, residues on the upper face of α2 also interact with the SAMHD1_mnd_ SAM domain (see below) so the cause of the α2 shift is unclear. Therefore, given that the mode of the N-terminal arm - α2 - α3 Vpx association with DCAF1 is a common feature of SIV_mnd-2_ and SIV_smm_ complexes and that nearly all the DCAF1-interacting residues are type-conserved in HIV-2/SIV Vpx proteins (Figure S5), it is likely that the same principles of DCAF1 binding apply to both the SIV_mnd-2_ and SIV_smm_ Vpx lineages.

### A Combined SIV_mnd-2_ Vpx/SAMHD1_mnd_(1-22)/DCAF1-CtD Ternary Interface

**Figure 4:**
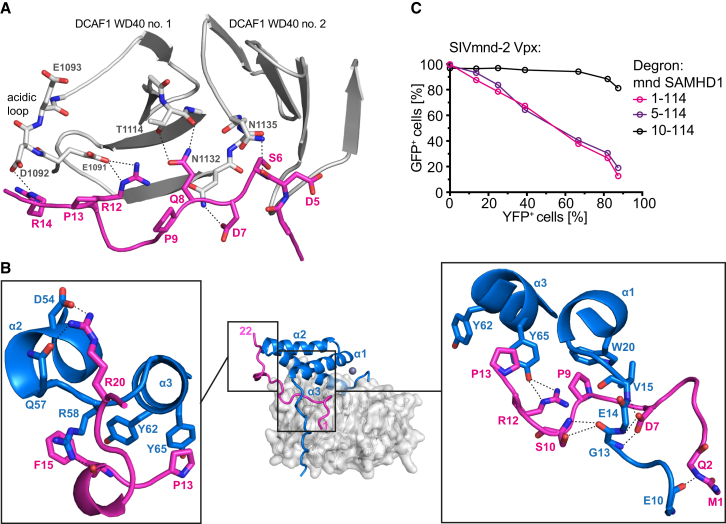
The Combined SIV_mnd-2_ Vpx/DCAF1-CtD/SAMHD1_mnd_-NtD Ternary Interface (A) Interface of DCAF1-CtD and SAMHD1_mnd_ NLS region. β strands from WD40 repeat-1 and -2 of DCAF1-CtD are shown in gray cartoon representation. The N-terminal NLS region of SAMHD1_mnd_ is shown in magenta. Residues making contacts at the interface are shown in stick representation with hydrogen bonding and salt bridge interactions displayed as dashed lines. (B) The Vpx-SAMHD1_mnd_ NLS region interface. The ternary complex is displayed (central) in the same orientation and representation as Figure 2. Details of SAMHD1-Vpx interactions within the boxed region are shown left and right. Residues in SAMHD1 (magenta) and Vpx (blue) making contacts at the interface are shown in stick representation with hydrogen bonding and salt bridge interactions displayed as dashed lines. (C) Quantification of reporter expression in stable cell lines containing degron constructs with the indicated N-terminal truncations of SAMHD1_mnd_. Cells were transduced with increasing titers of particles expressing SIV_mnd-2_ Vpx together with YFP and degron reporter expression (EGFP) measured by flow cytometry.

In the complex, the N-terminal NLS region (residues 1–22) of SAMHD1_mnd_ comprises an extended chain that binds to the edge of DCAF1 in an interface that buries 500 Å^2^ of solvent accessible surface (Figure 4A; Table S1B). Here, residues D5, D7, and Q8 make extensive sidechain and mainchain hydrogen bonds to residues in the interspersing loops of DCAF1 WD40 repeat-1. In addition, SAMHD1 residues R12 and R14, which are part of the SAMHD1 NLS (Brandariz-Nuñez et al., 2012; Hofmann et al., 2012), make salt bridges to E1091 and D1092 in the DCAF1 acidic loop. The ternary interaction is completed by a 750 Å^2^ SAMHD1_mnd_-SIV_mnd-2_ Vpx interface that includes multiple interactions with Vpx residues from the N-terminal arm, α1, and the α2-α3 loop (Figure 4B; Table S1C). One feature of this interface is a “trapped” region where the Vpx N-terminal arm wraps over SAMHD1 packing it against DCAF1. Within the trapped region, the main chains of SAMHD1 and Vpx interact through hydrogen bonding between E10, G13, and E14 of Vpx and Q2 and S10 of SAMHD1. In addition, the main chain of Vpx G13, and E14 also make interactions with the SAMHD1 D7 and S10 side chains. C-terminal to the trapped region there are further interactions where Vpx V15, L17, and W20 create a hydrophobic pocket at the N terminus of α1 that SAMHD1 P9 inserts into. Additionally, SAMHD1 NLS residue R12 makes a hydrogen bond to Vpx Y65, SAMHD1 F15 stacks with Vpx R58, and SAMHD1 R20 makes an electrostatic interaction with Vpx D54 (Figure 4B).

The functional importance of the Vpx trapping of the SAMHD1 N-terminal region was revealed in an EGFP-degron fusion protein degradation assay. Figure 4C shows that SIVmnd-2 Vpx induces efficient degradation of constructs containing SAMHD1 amino acids 1–114 and 5–114. In contrast, EGFP-SAMHD1 (10–114), which lacks D7 and Q8 that make hydrogen bonds to Vpx and DCAF1 within the trapped region, is not degraded by Vpx indicating the requirement of the trapping interaction for degron activity.

### The SIV_mnd-2_ Vpx/ SAMHD1_mnd_ SAM Domain Interface

**Figure 5:**
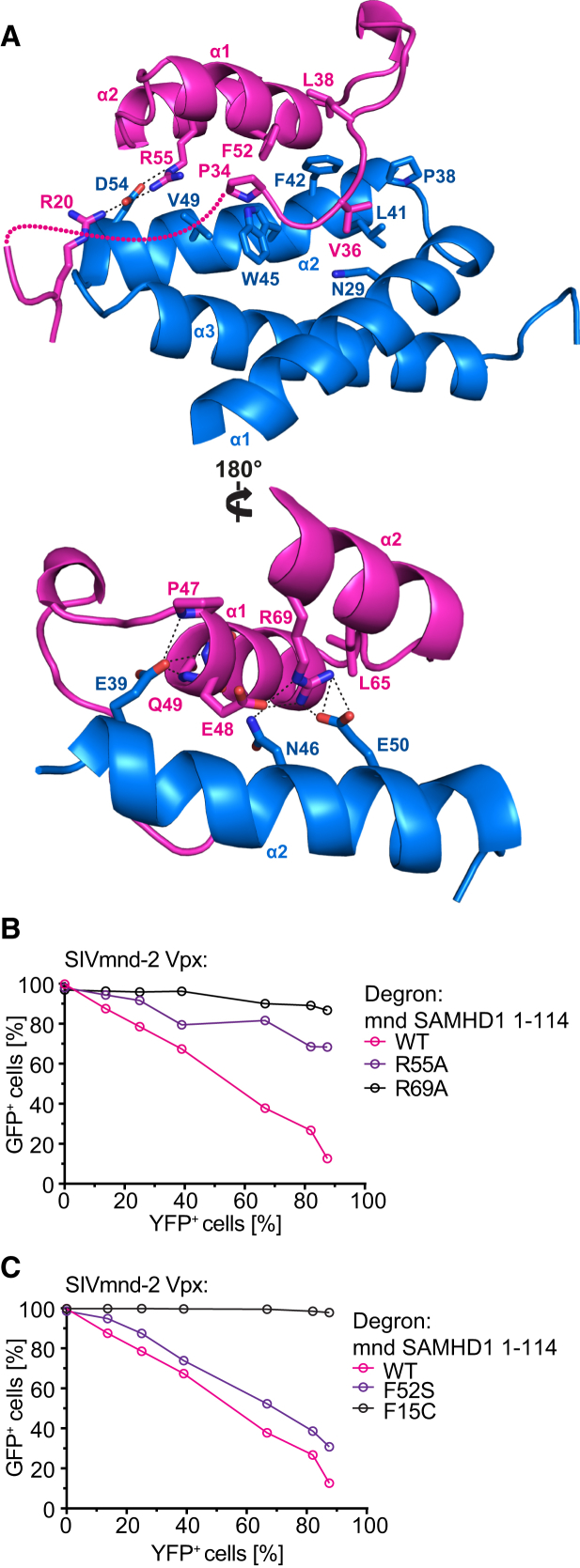
SAM Domain-SIV_mnd-2_ Vpx Interactions (A) The SAMHD1_mnd_ SAM domain-SIV_mnd-2_ Vpx interface. The SAMHD1_mnd_ SAM domain (magenta) and SIV_mnd-2_ Vpx (blue) are shown in cartoon representation. Residues making interactions are shown in stick representation with hydrogen bonding and salt bridges displayed as dashed lines. (B and C) Quantification of reporter expression in cells stably expressing SAMHD1_mnd_ (1–114) degron reporter constructs with (B) SAM domain point mutations and (C) mandrill to human SAMHD1 amino acid substitutions. Cells were transduced with increasing titers of particles expressing SIV_mnd-2_ Vpx, and the level of transduction (YFP) and degron reporter (EGFP) expression was measured by flow cytometry.

In addition to the ternary interface that “traps” the SAMHD1_mnd_ N terminus within the complex a further DCAF1-independent interaction between the SAM domain of SAMHD1_mnd_-NtD and SIV_mnd-2_ Vpx is also observed. This interface has a buried surface area of 580 Å^2^ and comprises residues that project upward from Vpx α2 that contact SAMHD1 residues N-terminal to the SAM domain helix 1 as well as those on the underside of the SAM domain in helices 1 and 2 (Figure 5A). The center of the interface comprises a hydrophobic core created by Vpx residues P38, L41, F42, W45, and V49 and SAMHD1 residues P34, V36, L38, and F52 (Figure 5A, upper panel). In addition to this hydrophobic patch, there are electrostatic interactions between SAMHD1 R55 and Vpx D54 (Figure 5A, upper panel), SAMHD1 R69 and Vpx E50 (Figure 5A, lower panel), as well as hydrogen bonding from Vpx E39 to the backbone of the first turn of SAM domain helix 1 (Figure 5B, lower panel). The contribution of residues in the SAM domain-Vpx interface to degron function was assessed by introduction of alanine mutations at SAMHD1 residues R55 and R69 in the EGFP-fusion reporter. Both residues make extensive electrostatic interactions at the interface, and the alanine substitutions result in a loss of reporter degradation (Figure 5B). R69 is also among a group of residues in the N-terminal region of SAMHD1 that have undergone positive evolutionary selection (Lim et al., 2012) indicative of an interaction hotspot between restriction factor and viral antagonist, further underlining the importance of Vpx-SAM domain contacts for induction of SAMHD1 proteasomal degradation.

Comparison of the sequences of human and mandrill SAMHD1-NtD regions reveals 13 amino-acid changes, nine in the SAM domain and four in the NLS region. Of these, only two make side chain contacts in the mandrill ternary complex, F15 in the NLS region and F52 in the SAM domain. In human SAMHD1, these residues are replaced by non-conservative cysteine and serine substitutions (Figure S6). In the mandrill complex, both phenylalanine residues make interactions, F15 stacks against the side chain of R58 in the Vpx α2-α3 loop (Figure 4B, left panel), and F52 is central to the hydrophobic SAM domain-Vpx interaction (Figure 5A). In order to assess their role in the specificity of the Vpx-SAMHD1 interaction, F15 and F52 were mutated to cysteine and serine that are found in human SAMHD1 and their contribution tested in the degron assay (Figure 5C). These data show the F15C mutation renders SAMHD1_mnd_ resistant to SIV_mnd-2_ Vpx-induced proteasomal degradation but that surprisingly the F52S mutation only slightly reduces degradation of the degron reporter protein. Nevertheless, introduction of the reverse mutations C15F and S52F in human SAMHD1 has previously been shown to render human SAMHD1 sensitive to SIV_mnd-2_ Vpx-mediated degradation (Wei et al., 2014). Therefore, taken together, these data support the idea that amino acid changes in primate SAM domain and NLS regions determine the lineage-specific formation of a Vpx-SAMHD1 interaction interface.

### Specificity of Vpx-SAMHD1-NtD and -CtD Interactions

**Figure 6:**
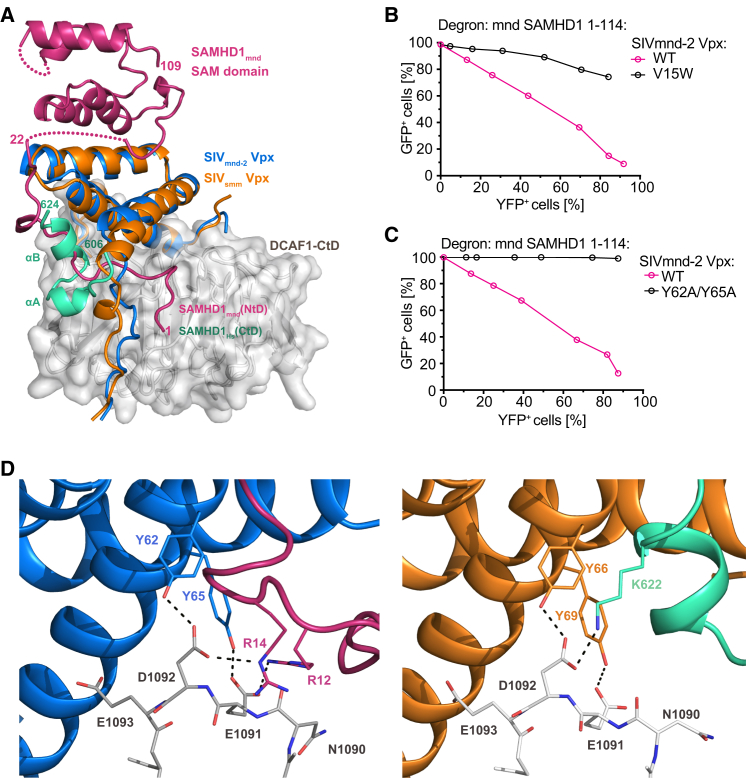
Comparison to the SIV_smm_ Vpx/DCAF1-CtD/SAMHD1_hs_-CtD Structure (A) Superposition of the SIV_mnd-2_ Vpx/DCAF1-CtD/SAMHD1_mnd_-NtD and SIV_smm_ Vpx/DCAF1-CtD/SAMHD1_hs_-CtD ternary complexes. Cartoons are colored as Figure 2 with the addition of SIV_smm_ Vpx (orange) and SAMHD1_hs_-CtD (green). For clarity, only DCAF1-CtD from the mandrill complex is shown. (B and C) Quantification of reporter expression in cells stably expressing the SAMHD1_mnd_ (1–114) degron reporter construct after transduction with increasing titers of particles expressing SIV_mnd-2_ Vpx with mutations in (B) the N-terminal arm (V15W) and in (C) the di-tyrosine motif (Y62A/Y65A). The level of Vpx transduction (YFP) and degron reporter (EGFP) expression was measured by flow cytometry. (D) Detailed view of the conserved VR2 Vpx di-tyrosine motif interaction with the DCAF1-CtD acidic loop and basic motifs of the SAMHD1_mnd_-NtD (left) and SAMHD1_hs_-CtD (right) degrons; see also Figure S5. Residues that contribute to the ternary interface are shown in stick representation with hydrogen bonding and salt bridge interactions displayed as dashed lines; see also Figure S6.

Despite similar overall structure and an identical mode of DCAF1 interaction (Figure S4), the SIV_mnd-2_ Vpx/DCAF1 assembly recognizes the SAMHD1_mnd_-NtD, while the HIV-2/SIV_mac_/SIV_smm_-type Vpx/DCAF1 are specific for the SAMHD1 C terminus. Comparison of the SIV_mnd-2_ system presented here with the previously determined SIV_smm_/DCAF1/SAMHD1-Ctd assembly (Schwefel et al., 2014) reveals the structural determinants for these fundamentally different specificities. In both complexes, a Vpx N-terminal arm and residues at the N terminus of α1 make contacts with either the amino-terminal region in SAMHD1_mnd_ or the CtD region of SAMHD1_hs_. The location of this contact on the DCAF1 disk is similar in both combined ternary interfaces (Figure 6A). However, the SAMHD1_mnd_ N terminus makes additional contacts and occupies a larger surface area on Vpx and DCAF1, 750 Å^2^ and 500 Å^2^ versus 710 Å^2^ and 210 Å^2^ for the SAMHD1_hs_ C terminus. In addition, in the mandrill complex, the Vpx arm folds over the first 20 residues of SAMHD1_mnd_ trapping it against DCAF1, while the human SAMHD1 C-terminal peptide associates rather peripherally to the DCAF1/Vpx interface (Figure 6A).

In this context, it is important to note that Vpx amino acid residues at the N terminus of α1 that contribute to SAMHD1 binding differ significantly between SIV_smm_ and SIV_mnd-2_ type Vpx proteins constituting a variable region (VR1) (Figure S5). In the SIV_smm_ complex, VR1 side chains of the conserved GEET-motif form salt bridges with basic residues in SAMHD1-CtD (Schwefel et al., 2014). However, in SIV_mnd-2_ Vpx, VR1 comprises a proline (P9^mnd-2^) followed by two glycine residues interspersed with a small hydrophobic amino acid and then a conserved acidic and hydrophobic residue (^9^P[x]GAG[D/E]V/A^15^). This configuration specifically accommodates the SAMHD1_mnd_-NtD and sandwiches it between Vpx and the DCAF1 disk. The importance of this VR1 interaction for Vpx-mediated proteasomal degradation of SAMHD1_mnd_ is demonstrated in EGFP-degron fusion protein assays where substitution of valine by bulky tryptophan at position 15 of SIV_mnd-2_ Vpx substantially reduces degron reporter protein degradation (Figure 6B). In addition, analysis of the clustering of VR1 sequences (Figure S5) shows that SIV_rcm_ and SIV_drl-1_ Vpx that are SAMHD1-NtD specific also contain a SIV_mnd-2_ type VR1, supporting the idea that VR1 is a major determinant for discrimination between SAMHD1 N-terminal and C-terminal target sequences.

As well as VR1, which confers NtD and CtD degron specificity to Vpx, a second region, VR2, in the α2-α3 loop and N terminus of α3 is conserved among Vpx but not in Vpr (Figure S5). Here, a di-tyrosine motif (Y62^mnd-2^/Y66^smm^ and Y65^mnd-2^/Y69^smm^) is present, which engages the DCAF1 acidic loop (Figures 3B and S4B), providing a hydrophobic platform for binding of SAMHD1 residues (P13^mnd^ or V618^hs^/F621^hs^) and for positively charged amino acids (R12^mnd^/R14^mnd^ or K622^hs^) to form salt bridges to the DCAF1 acidic loop (Figure 6D). The importance of the Vpx di-tyrosine pair at the center of a ternary DCAF1-degron interaction was demonstrated in the degron assay where introduction of a double tyrosine to alanine mutant into SIV_mnd-2_ Vpx abolished reporter degradation (Figure 6C). Similarly, mutations of the basic residues that make salt bridges to the DCAF1 acidic loop also prevent degron reporter degradation (Schwefel et al., 2014) and in a pigtail macaque model SIV_mne_, a Vpx Y66A, Y69A, Y71A triple mutant causes a reduction of viral load (Belshan et al., 2012). Taken together, these observations suggest that the VR2 region contains common binding determinants utilized in both SIV_mnd-2_ and SIV_smm_ type Vpx molecules for recognition of SAMHD1-NtD and SAMHD1-CtD degrons.

## Discussion

The current properties of accessory proteins Vpr and Vpx likely reflect a lengthy history of conflict between lentiviruses and their hosts. Eight different lineages of exogenous primate lentivirus including HIV-1 can be identified. All encode a Vpr that induces host G2/M cell-cycle arrest (Berger et al., 2015), probably by targeting a still poorly defined host cell protein for degradation through the proteasomal pathway or through interaction with proteins of the DNA damage pathway (Laguette et al., 2014). Vpr from two viral clades, typified by SIV_agm_ and SIV_syk_, is also able to target SAMHD1 for degradation, facilitating infection of some types of non-dividing cells. In two further clades, both Vpr and Vpx are present with Vpr retaining only cell-cycle arrest activity and Vpx only inducing SAMHD1 degradation.

Phylogenetic reconstructions suggest that the Vprs from SIV_agm_ and SIV_syk_ acquired anti-SAMHD1 activity prior to the molecular events leading to the generation of Vpx (Lim et al., 2012). Moreover, it appears that once Vpr acquires anti-SAMHD1 activity, it does not lose it. This would imply that viruses containing both Vpr and Vpx arose by the recombination of two viruses; the first encoding an ancestral Vpr with the dual ability to cause cell-cycle arrest and degrade SAMHD1 and the second with a Vpr that had not acquired the capacity to induce SAMHD1 degradation. This allowed the ancestral, dual function Vpr to convert to Vpx and maintain SAMHD1 degradation activity but loose the cell-cycle arrest function provided by the second Vpr. Evaluation of our crystal structures is most informative in understanding these evolutionary changes and specifically the interaction between (i) Vpr/x and DCAF1 and (ii) between the two classes of Vpx and their SAMHD1 targets.

Amino acid residues that bind to DCAF1 and those that co-ordinate the bound zinc are highly conserved in all lentiviral Vpr/Vpx proteins (Figure S5), suggesting that the overall structure of the protein as well as mode of DCAF1 binding have been conserved during Vpr/Vpx evolution. This implies that the HIV-1 Vpr/DCAF1 association is likely to be similar to the one observed in the SIV_mnd-2_ and SIV_smm_ (Schwefel et al., 2014) Vpx/DCAF1 crystal structures (Figure S4). Therefore, these structures might provide suitable starting points for rational design of compounds interfering with HIV-1 Vpr function even in the absence of definitive information concerning its cellular target. A notable exception is Vpr from SIV_deb_ that induces SAMHD1 degradation apparently without binding to DCAF1 (Berger et al., 2015), and it will be interesting to determine whether it utilizes an alternative pathway or different receptor protein to target SAMHD1.

Vpx from SIV_smm_ and SIV_mnd-2_ have been shown to interact with the C- and N-terminal regions of SAMHD1, respectively (Fregoso et al., 2013). Comparison of Vpx/SAMHD1/DCAF1 ternary complexes reveals that this discrimination of SAMHD1 N- and C-terminal degrons by the two classes of Vpx is determined both by polymorphisms in the SAMHD1 amino-terminal region (Figure S6) and by specific sequences in the VR1 region of Vpx (Figures 6 and S5). By contrast, in both classes of Vpx the VR2 region contains a conserved di-tyrosine pair that interacts with the DCAF1 acidic loop to provide a hydrophobic platform for N- and C-terminal SAMHD1 degrons. In this way, basic residues from both degron types are positioned for electrostatic interactions with the DCAF1 acidic loop. Interestingly, only the second tyrosine is present in SIV_agm_ Vpr proteins, which are incapable of inducing human SAMHD1 degradation, while the position corresponding to the first tyrosine is occupied by a glutamate residue (Figure S5). Gain of the bi-tyrosine motif might therefore have been a critical step in the development of Vpx allowing multiple modes of binding SAMHD1 while at the same time resulting in loss of the Vpr capacity to cause cell-cycle arrest.

A third highly variable region (VR3) in Vpr/Vpx proteins is directly C-terminal to the fourth conserved zinc-coordinating residue (Figure S5) that in both SIV_smm_ and SIV_mnd-2_ type Vpx contains proline-rich stretches of variable length. In the corresponding SAMHD1/Vpx/DCAF1 crystal structures, VR3 is either disordered (this study) or involved in crystal contacts (Schwefel et al., 2014), strongly suggesting that the region is not involved in SAMHD1 degron recognition. In agreement, SIV_mac_ Vpx lacking the poly-proline tail is still able to assemble into a ternary SAMHD1/Vpx/DCAF1 complex and to induce cullin-4-dependent SAMHD1 poly-ubiquitination (Ahn et al., 2012) and degradation (Belshan et al., 2012). Functional studies suggest involvement of VR3 in nuclear localization of Vpx and Vpr (Pancio et al., 2000; Zhou et al., 1998) with consequences for viral replication (Belshan et al., 2012), but notably, mutations in this region also influence protein expression levels significantly (Miyake et al., 2014).

Predictions regarding DCAF1/Vpr-mediated recruitment of target protein(s) that result in G2/M cell-cycle arrest remain speculative. In HIV-1, VR2 differs significantly from other Vpr and Vpx proteins, suggesting that in contrast to the Vpx di-tyrosine/DCAF1 acidic loop/SAMHD1 interaction, other principles may apply to HIV-1 Vpr target recognition. By contrast, VR1 of HIV-1 Vpr contains a ^12^REP[F/Y/W][D/N]^16^ motif that is also conserved in SIV_agm_ Vpr proteins (Figure S5), suggesting similarities in VR1-mediated SIV_agm_ and HIV-1 Vpr target recruitment. Therefore, considering recent evidence for HIV-1 Vpr/DCAF1 association with components of the SLX4/MUS81/EME1 DNA-repair complex (Berger et al., 2015; Laguette et al., 2014), structural analysis of SIV_agm_ Vpr/DCAF1 together with the cognate SAMHD1_agm_ will be of interest to further delineate the degron and/or target sequence(s) recognized by HIV-1 Vpr.

## Experimental Procedures

### Degron Assay

Degron reporter constructs were generated by replacing the human SAMHD1-CtD degron sequence in pCMS28-NLS-EGFP-SAMHD1-CtD with N-terminal sequences from SAMHD1_mnd_. Point mutations were created by site-directed mutagenesis. Virus-like particles (VLPs) were generated by co-transfecting 293T cells with pVSVG, pKB4 and pCMS28-NLS-EGFP-SAMHD1. Stable cell lines were produced by transduction of *Mus dunni* cells followed by puromycin selection. Expression of degron constructs was assessed using western blotting with anti-EGFP antibodies.

SIV_smm_ or SIV_mnd-2_ Vpx sequences were transferred into pLgatewayIeYFP (Gateway LR clonase™ II, Invitrogen) to create bicistronic Vpx-IRES-YFP expression constructs. Point mutations were created by site-directed mutagenesis. VLPs expressing Vpx-IRES-YFP were generated by co-transfecting 293T cells with pVSV-G, pKB4, and pLgatewayIeYFP-Vpx.

Parental *Mus dunni* or stable cell lines expressing degron reporters were seeded at 5 × 10^4^ cells per well in a 24-well plate 1 day prior to infection with 2-fold serial dilutions of Vpx-YFP VLPs. After 48 hr, the percentage of EGFP- and YFP-positive cells was determined by flow cytometry.

### Protein Expression and Purification

The nucleotide sequences coding for SIV_mnd-2_ Vpx isolate 5440 and amino acid residues 1–114 of SAMHD1_mnd_ were inserted into pET-49b and pET-52b (Merck Millipore) expression plasmids respectively to generate N-terminally GST-tagged and Strep-II-tagged fusion proteins. SAMHD1 (1–114) was expressed in *E. coli* strain Rosetta 2 and purified using Strep-Tactin affinity followed by size-exclusion chromatography. SIV_mnd-2_ Vpx was captured from the bacterial lysate onto Glutathione Sepharose (GSH-Sepharose) (GE Healthcare) prior to an assembly reaction (see below). His-tagged DCAF1, residues 1,058–1,396 (DCAF1-CtD), was expressed in insect cells and purified using Ni-NTA Sepharose and size-exclusion chromatography.

For assembly, the GST-SIV_mnd-2_ Vpx bound beads were resuspended and incubated with 1 mg of DCAF1-CtD, an equimolar amount of SAMHD1_mnd_-NtD, and 1 mg of HRV-3C protease (GE Healthcare) overnight at 4°C. After removal of the beads by centrifugation, the eluted ternary complex was further purified by size-exclusion chromatography. Details of protein expression constructs, protein production, and storage are provided in Supplemental Experimental Procedures.

### Crystallization and Structure Solution

Crystals of the SIV_mnd-2_ Vpx/SAMHD1_mnd_-NtD/DCAF1CtD complex were grown using the hanging drop vapor diffusion at 18°C in 2 μl droplets comprising 1 μl complex (6.34 mg/ml) and 1 μl of reservoir solution (0.16 M Trisodium Citrate-HCl [pH 5.2] and 4% PEG 6000). X-ray diffraction data were collected on beamline I04 at the Diamond Light Source, UK at a wavelength of 0.97965 Å. The structure was solved by molecular replacement using the previously determined DCAF1-CtD structure, and a homology model constructed with the previously determined SIV_smm_ Vpx as template (PDB: 4CC9). Details of crystallization, data collection, processing, and structure solution are provided in Supplemental Experimental Procedures.

### Multiple Sequence Alignment

Amino acid sequences were aligned using the ClustalW server and adjusted manually. NCBI accession numbers for Vpr, Vpx, and SAMHD1 are provided in Supplemental Experimental Procedures.

## Author Contributions

D.S., V.C.B., E.C., and P.A.W. performed experiments. D.S., V.C.B., E.C., P.A.W., J.P.S., K.N.B., and I.A.T. contributed to experimental design, data analysis, and manuscript writing. The authors declare no competing financial interests.

## Figures and Tables

**Table 1:** X-Ray Data Collection and Refinement Statistics

	mnd-8498
**Data collection**	

Space group	P6_5_22
Cell dimensions: *a*, *b*, *c* (Å)	102.04, 102.04, 265.09
Cell dimensions: α, β, γ (°)	90, 90, 120
Resolution (Å)	30 (2.81)^1^ – 2.65
*R*_sym_ or *R*_merge_ (%)	7.9 (145.3)
CC_1/2_^2^	99.9 (59.9)
*I*/σ*I*	20.70 (1.63)
Completeness (%)	99.6 (99.3)
Redundancy	9.4 (9.7)

**Refinement**	

Resolution (Å)	30 – 2.65
No. reflections	24,554
*R*_work_/*R*_free_	17.5/23.1
No. atoms: Protein	3,817
No. atoms: zinc ion	1
No. atoms: water	2
*B* factors (Å)^2^:	
*B* factors (Å)^2^: protein	87.6
*B* factors (Å)^2^: zinc ion	105.1
*B* factors (Å)^2^: water	71.15
rms deviations: bond lengths (Å)	0.008
rms deviations: bond angles (°)	1.183

1 Values in parentheses are for highest-resolution shell. A single crystal was used for data collection.

2 High-resolution cutoff based on criteria according to Karplus and Diederichs (2012).

## Accession Numbers

The coordinates and structure factors of the SIV_mnd-2_-Vpx/SAMHD_mnd_-NtD/DCAF1-CtD ternary complex have been deposited in the Protein Data Bank (PDB: 5aja).

## Supplemental Information

Document S1. Figures S1–S6, Table S1A–S1C, and Supplemental Experimental Procedures

Document S2. Article plus Supplemental Information
