# Supplementary material for: Molecular Determinants for Recognition of Divergent SAMHD1 Proteins by the Lentiviral Accessory Protein Vpx
Source: Cell Host Microbe. 2015 Apr 8;17(4):489–99. doi: 10.1016/j.chom.2015.03.004 (PMC4400269; doi:10.1016/j.chom.2015.03.004)
Supplement: Document S1. Figures S1–S6, Table S1A–S1C, and Supplemental Experimental Procedures [file mmc1.pdf]

Cell Host & Microbe, Volume *17*

Supplemental Information

Molecular Determinants for Recognition

of Divergent SAMHD1 Proteins

by the Lentiviral Accessory Protein Vpx

David Schwefel, Virginie C. Boucherit, Evangelos Christodoulou, Philip A. Walker, Jonathan P. Stoye, Kate N. Bishop, and Ian A. Taylor

Molecular determinants for recognition of divergent SAMHD1 proteins by the lentiviral accessory protein Vpx.

INVENTORY OF SUPPLEMENTAL INFORMATION

ITEM 1) **Figure S1**. Western blot analysis of expression and primary FACS data for SAMHD1 degron assay. Panel **A** of this item shows the level of expression of each SAMHD1 degron used in the study. Panel **B** shows the primary FACS data from individual degron assays. Both **A** and **B** are associated with Figures 1, 4, 5 and 6 in the main article.

ITEM 2) **Figure S2**. Experimental electron density for each component of the SIV_{mnd-2} Vpx/$SAMHD_{mnd}$-NtD/DCAF1-CtD ternary complex. This item shows the quality of the maps used to build the structure presented in the paper and is associated with Table 1 and Figures 2-6 in the main article.

ITEM 3) **Figure S3**. Zinc ion co-ordination of Vpx SIV_{mnd-2} and Vpx SIV_{smm}. This item shows the details of the co-ordinated zinc ions bound in the Vpx SIV_{mnd-2} and Vpx SIV_{smm} structures. It is associated with Figure 2 in the main article.

ITEM 4) **Figure S4**. Superposition of Vpx SIV_{mnd-2} and Vpx SIV_{smm} ternary complexes. This item shows the structural overlap between Vpx SIV_{mnd-2} and Vpx SIV_{smm} at the DCAF1 interface. This alignment facilitates a comparison of the common interactions and is associated with Figure 3 of the main article.

ITEM 5) **Figure S5**. Multiple sequence alignment of Vpx and Vpr proteins. This alignment in combination with Vpx SIV_{mnd-2} and Vpx SIV_{smm} structures shows all the conserved and variable regions found in the different Vpx and Vpr lineages and is associated with Figure 3 and 6 of the main article.

ITEM 6) **Figure S6**. SAMHD1 multiple sequence alignment. This alignment shows the sequence conservation in SAMHD1 NtD and CtD degron regions and highlights the residues making contacts at the Vpx interface. It is associated with Figure 5 and 6 of the main article.

ITEM 7) Supplemental Figure legends. Detailed legends for Supplemental Figures S1-S6.

ITEM 8) **Supplemental Table S1A-C**. These tables provide a list of all the interactions observed in the SIV_{mnd-2} Vpx/$SAMHD_{mnd}$-NtD/DCAF1-CtD ternary complex and are associated Figures 3-6 of the main article.

ITEM 9) SUPPLEMENTAL EXPERIMENTAL PROCEDURES

This item provides detailed methods to supplement the experimental procedures reported in the paper.

ITEM 10) SUPPLEMENTAL REFERENCES

This item contains the full references that are cited Supplemental Experimental Procedures

Figure S1

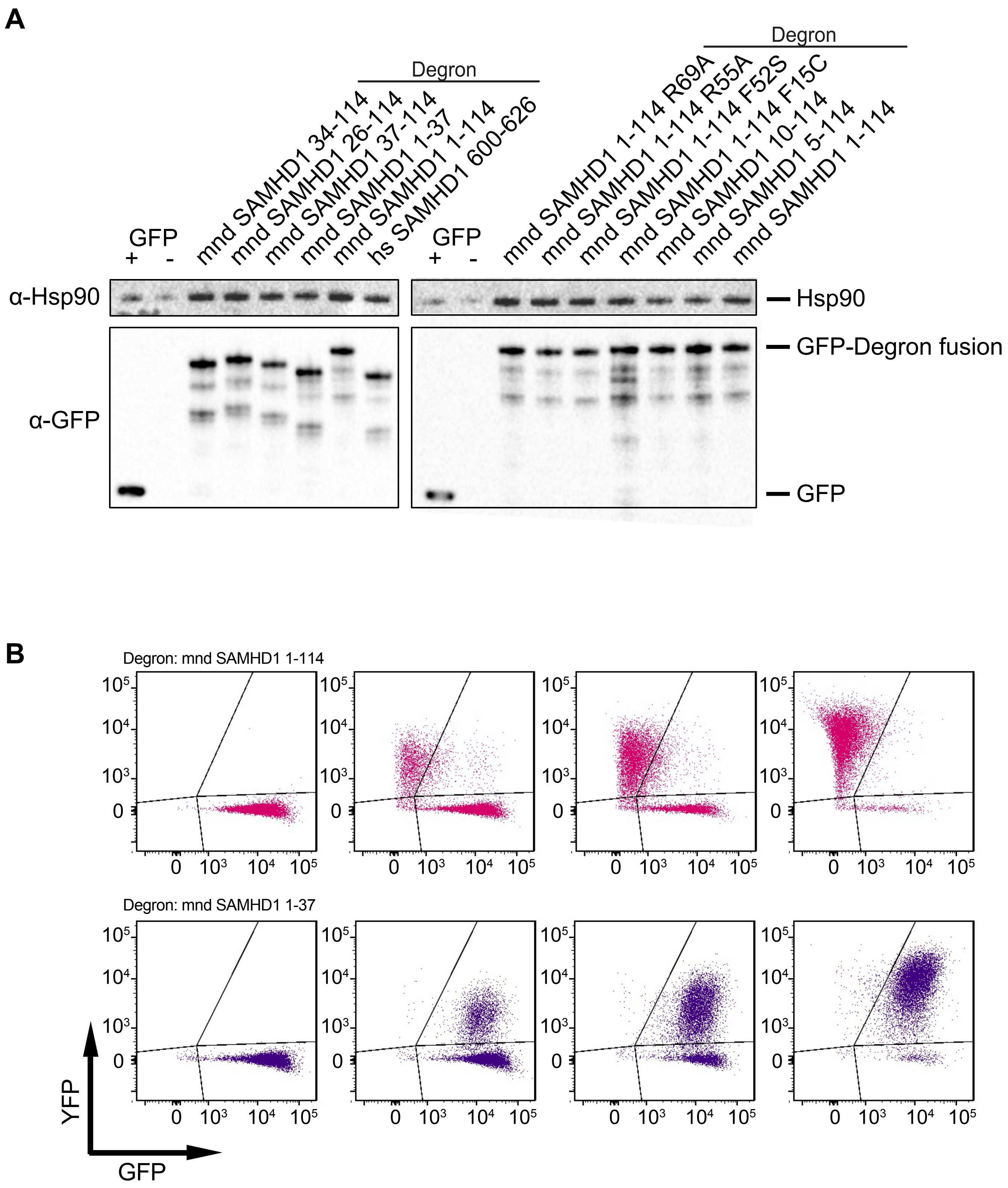
A
Degron
Degron
mnd SAMHD1 34-114
mnd SAMHD1 26-114
mnd SAMHD1 37-114
mnd SAMHD1 1-37
mnd SAMHD1 1-114
hs SAMHD1 600-626
mnd SAMHD1 1-114 R69A
mnd SAMHD1 1-114 R55A
mnd SAMHD1 1-114 F52S
mnd SAMHD1 1-114 F15C
mnd SAMHD1 10-114
mnd SAMHD1 5-114
mnd SAMHD1 1-114
GFP
+ -
α-Hsp90
α-GFP
Hsp90
GFP-Degron fusion
GFP
B
Degron: mnd SAMHD1 1-114
Degron: mnd SAMHD1 1-37
YFP
GFP

Figure S2

DCAF1-CtD

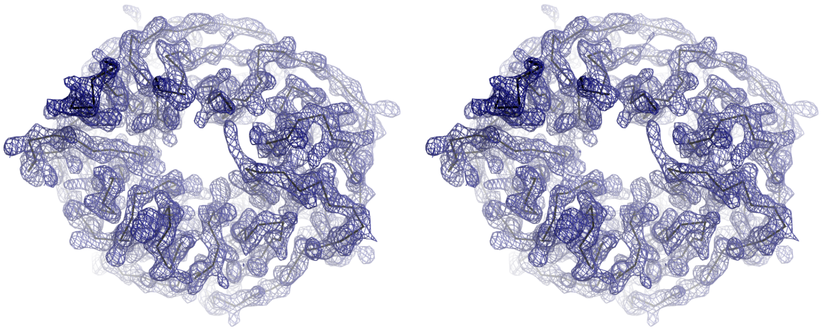

SIV_{mnd-2} Vpx

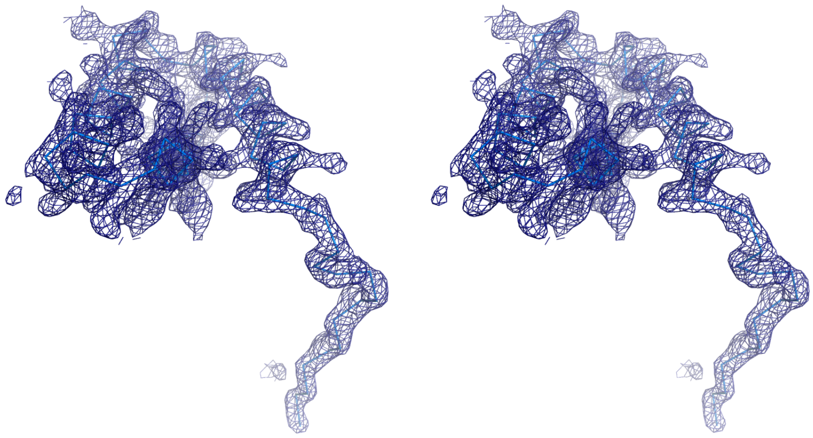

$SAMHD1_{mnd}$ 1-114

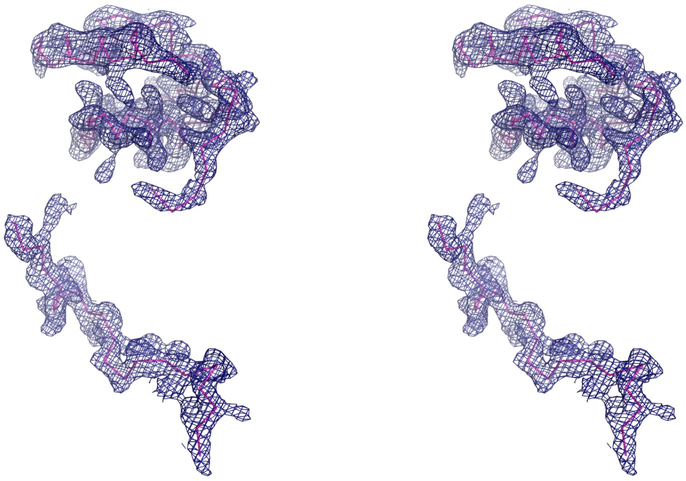

Figure S3

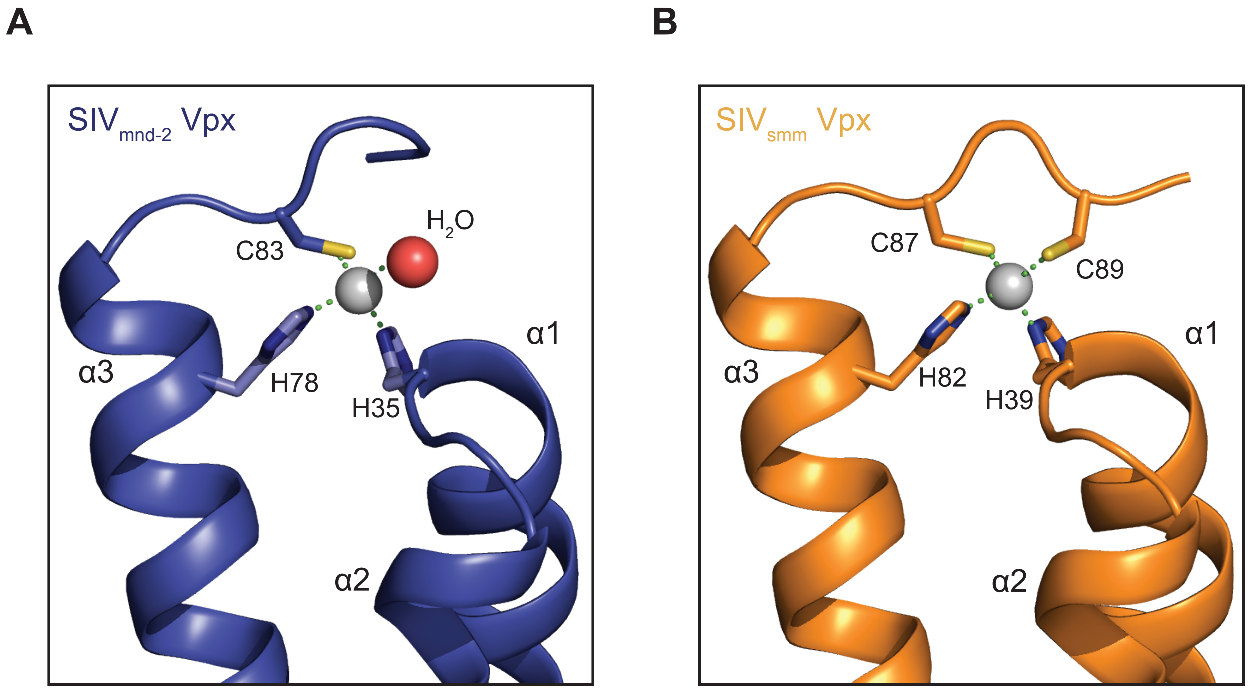
A
B
SIVmnd-2 Vpx
H2O
C83
α3
H78
H35
α1
α2
SIVsmm Vpx
C87
C89
α3
H82
H39
α1
α2

Figure S4

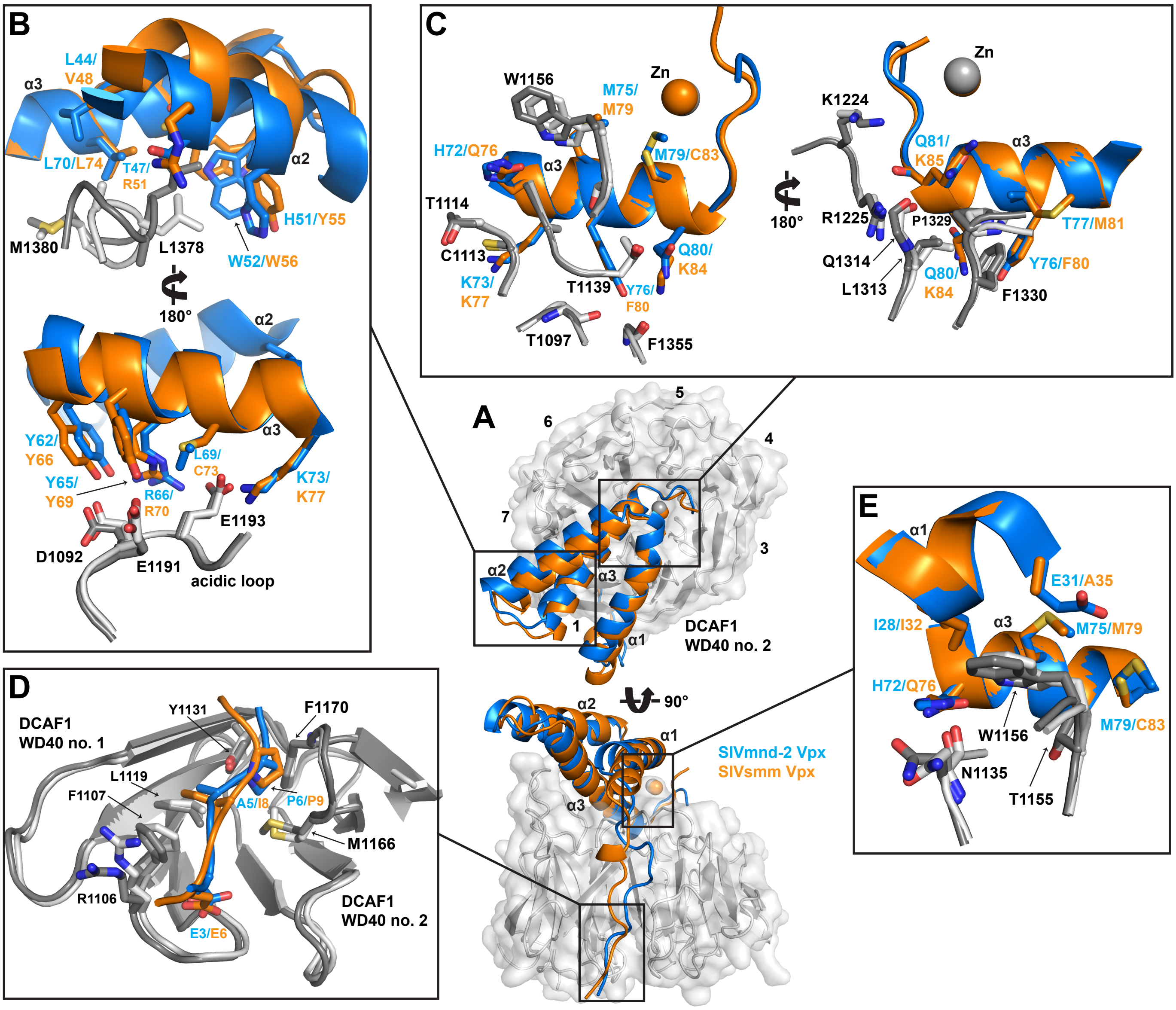

B
L44/
V48
α3
L70/L74
T47/
R51
α2
M1380
L1378
H51/Y55
W52/W56
180°
α2
α3
Y62/
Y66
Y65/
Y69
R66/
R70
L69/
C73
K73/
K77
E1193
D1092
E1191
acidic loop
C
W1156
Zn
M75/
M79
H72/Q76
α3
M79/C83
T1114
C1113
K73/
K77
T1139
Y76/
F80
Q80/
K84
T1097
F1355
Zn
K1224
Q81/
K85
α3
180°
R1225
P1329
T77/M81
Q1314
L1313
Q80/
K84
Y76/F80
F1330
A
6
5
4
7
3
α2
α3
1
α1
DCAF1
WD40 no. 2
α2
90°
α1
α3
SIVmnd-2 Vpx
SIVsmm Vpx
D
DCAF1
WD40 no. 1
Y1131
F1170
L1119
F1107
A5/I8
P6/P9
M1166
R1106
E3/E6
DCAF1
WD40 no. 2
E
α1
E31/A35
I28/I32
α3
M75/M79
H72/Q76
M79/C83
W1156
N1135
T1155

Figure S5

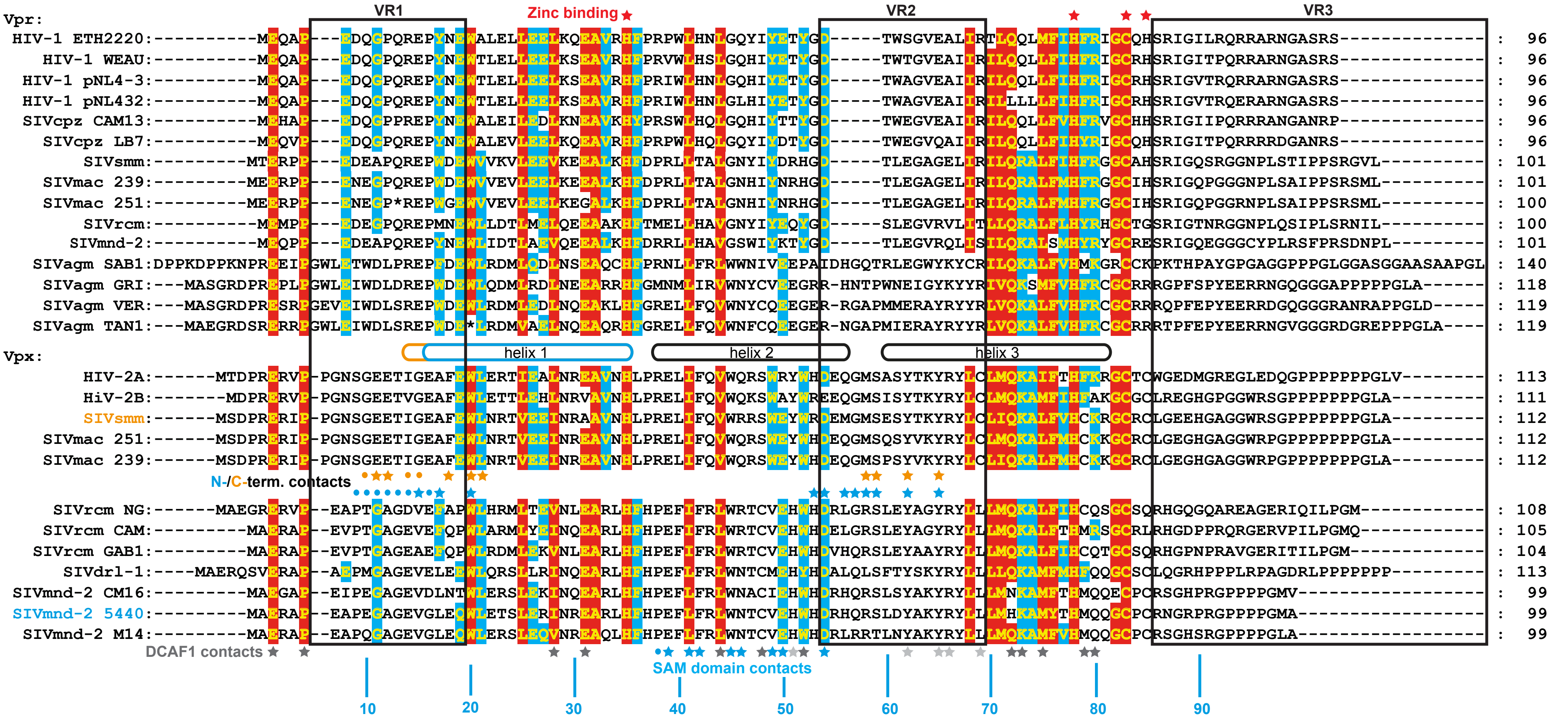

VR1
Zinc binding
VR2
VR3
Vpr:
HIV-1 ETH2220:-----------MEQAP---EDQGPQREPYNEWALELLEELKQEAVRHFPRPWLHNLGQYIYETYGD-----TWSGVEALIRTLQQLMFIHFRIGCQHSRIGILRQRRARNGASRS-------------- : 96
HIV-1 WEAU:-----------MEQAP---EDQGPQREPYNEWTLELLEELKSEAVRHFPRVWLHSLGQHIYETYGD-----TWTGVEAIIRILQQLLFIHFRIGCRHSRIGITPQRRARNGASRS-------------- : 96
HIV-1 pNL4-3:-----------MEQAP---EDQGPQREPYNEWTLELLEELKSEAVRHFPRIWLHNLGQHIYETYGD-----TWAGVEAIIRILQQLLFIHFRIGCRHSRIGVTRQRRARNGASRS-------------- : 96
HIV-1 pNL432:-----------MEQAP---EDQGPQREPYNEWTLELLEELKSEAVRHFPRIWLHNLGLHIYETYGD-----TWAGVEAIIRILLLLLFIHFRIGCRHSRIGVTRQERARNGASRS-------------- : 96
SIVcpz CAM13:-----------MEHAP---EDQGPPREPYNEWALEILEDLKNEAVKHYPRSWLHQLGQHIYTTYGD-----TWEGVEAIIRILQQLLFVHFRVGCHHSRIGIIPQRRRANGANRP-------------- : 96
SIVcpz LB7:-----------MEQVP---EDQGPQREPYNEWALEVLEELKQEAVRHFPRPWLHQLGQYIYDTYGD-----TWEGVQAIIRILQQLLFIHYRIGCQHSRIGITPQRRRRDGANRS-------------- : 96
SIVsmm:----------MTERPP---EDEAPQREPWDEWVVKVLEEVKEEALKHFDPRLLTALGNYIYDRHGD-----TLEGAGELIRILQRALFIHFRGGCAHSRIGQSRGGNPLSTIPPSRGVL---------- : 101
SIVmac 239:----------MEERPP---ENEGPQREPWDEWVVEVLEELKEEALKHFDPRLLTALGNHIYNRHGD-----TLEGAGELIRILQRALFMHFRGGCIHSRIGQPGGGNPLSAIPPSRSML---------- : 101
SIVmac 251:----------MEERPP---ENEGP*REPWGEWVVEVLEELKEGALKHFDPRLLTALGNHIYNRHGD-----TLEGAGELIRILQRALFMHFRGGCIHSRIGQPGGRNPLSAIPPSRSML---------- : 100
SIVrcm:-----------MEMPP---EDEGPQREPMNEWLLDTLMELQEEAAKHFTMELLHAVGNYIYEQYGD-----SLEGVRVLITLLQRALFLHYRHGCTGSRIGTNRGGNPLQSIPLSRNIL---------- : 100
SIVmnd-2:-----------MEQPP---EDEAPQREPYNEWLIDTLAEVQEEALKHFDRRLLHAVGSWIYKTYGD-----TLEGVRQLISILQKALSMHYRYGCRESRIGQEGGGCYPLRSFPRSDNPL--------- : 101
SIVagm SAB1:DPPKDPPKNPREEIPGWLETWDLPREPFDEWLRDMLQDLNSEAQCHFPRNLLFRLWWNIVEEPAIDHGQTRLEGWYKYCRILQKALFVHMKGRCCKPKTHPAYGPGAGGPPPGLGGASGGAASAAPGL : 140
SIVagm GRI:---MASGRDPREPLPGWLEIWDLDREPWDEWLQDMLRDLNEEARRHFGMNMLIRVWNYCVEEGRR-HNTPWNEIGYKYYRIVQKSMFVHFRCGCRRRGPFSPYEERRNGQGGGAPPPPPGLA------ : 118
SIVagm VER:---MASGRDPRESRPGEVEIWDLSREPWDEWLRDMLEDLNQEAKLHFGRELLFQVWNYCQEEGER-RGAPMMERAYRYYRLVQKALFVHFRCGCRRRQPFEPYEERRDGQGGGRANRAPPGLD----- : 119
SIVagm TAN1:---MAEGRDSRERRPGWLEIWDLSREPWDE*LRDMVAELNQEAQRHFGRELLFQVWNFCQEEGER-NGAPMIERAYRYYRLVQKALFVHFRCGCRRRTPFEPYEERRNGVGGGRDGREPPPGLA---- : 119
helix 1
helix 2
helix 3
Vpx:
HIV-2A:------MTDPRERVP-PGNSGEETIGEAFEWLERTIEALNREAVNHLPRELIFQVWQRSWRYWHDEQGMSASYTKYRYLCLMQKAIFTHFKRGCTCWGEDMGREGLEDQGPPPPPPPGLV-------- : 113
HiV-2B:-------MDPRERVP-PGNSGEETVGEAFEWLETTLEHLNRVAVNHLPRELIFQVWQKSWAYWREEQGMSISYTKYRYLCLMQKAMFIHFAKGCGCLREGHGPGGWRSGPPPPPPPGLA--------- : 111
SIVsmm:------MSDPRERIP-PGNSGEETIGEAFEWLNRTVEEINRAAVNHLPRELIFQVWRRSWEYWRDEMGMSESYTKYRYLCLIQKALFVHCKRGCRCLGEEHGAGGWRSGPPPPPPPGLA--------- : 112
SIVmac 251:------MSDPRERIP-PGNSGEETIGEAFEWLNRTVEEINREAVNHLPRELIFQVWQRSWEYWHDEQGMSQSYVKYRYLCLMQKALFMHCKKGCRCLGEGHGAGGWRPGPPPPPPPGLA--------- : 112
SIVmac 239:------MSDPRERIP-PGNSGEETIGEAFEWLNRTVEEINREAVNHLPRELIFQVWQRSWEYWHDEQGMSPSYVKYRYLCLIQKALFMHCKKGCRCLGEGHGAGGWRPGPPPPPPPGLA--------- : 112
N-/C-term. contacts
SIVrcm NG:------MAEGRERVP--EAPTGAGDVEFAPWLHRMLTEVNLEARLHFHPEFIFRLWRTCVEHWHDRLGRSLEYAGYRYLLLMQKALFIHCQSGCSQRHGQGQAREAGERIQILPGM------------ : 108
SIVrcm CAM:----------MAERAP--EVPTGAGEVEFQPWLARMLYEINQEARLHFHPEFIFRLWRTCVEHWHDELGRSLEYAGYRYLLLMQKALFTHMRSGCRLRHGDPPRQRGERVPILPGMQ------------ : 105
SIVrcm GAB1:----------MAERAP--EVPTGAGEAEFQPWLRDMLEKVNLEARLHFHPEFIFRLWRTCVEHWHDVHQRSLEYAAYRYLLLMQKALFIHCQTGCSQRHGPNPRAVGERITILPGM------------ : 104
SIVdrl-1:----MAERQSVERAP--AEPMGAGEVELEEWLQRSLLRINQEARLHFHPEFLFRLWNTCMEHYHDALQLSFTYSKYRYLLLLQKAMFMHFQQGCSCLQGRHPPPLRPAGDRLPPPPPPP--------- : 113
SIVmnd-2 CM16:----------MAEGAP--EIPEGAGEVDLNTWLERSLEKINQEARLHFHPEFLFRLWNACIEHWHDRHQRSLSYAKYRYLLLMNKAMFTHMQQECPCRSGHPRGPPPPGMV------------------ : 99
SIVmnd-2 5440:----------MAERAP--EAPEGAGEVGLEQWLETSLERINREARLHFHPEFLFRLWNTCVEHWHDRHQRSLDYAKYRYLLLMHKAMYTHMQQGCPCRNGRPRGPPPPGMA------------------ : 99
SIVmnd-2 M14:----------MAERAP--EAPQGAGEVGLEQWLERSLEQVNREAQLHFHPEFLFRLWNTCVEHWHDRLRRTLNYAKYRYLLLMQKAMFVHMQQGCPCRSGHSRGPPPPGLA------------------ : 99
DCAF1 contacts
SAM domain contacts
10
20
30
40
50
60
70
80
90

Figure S6

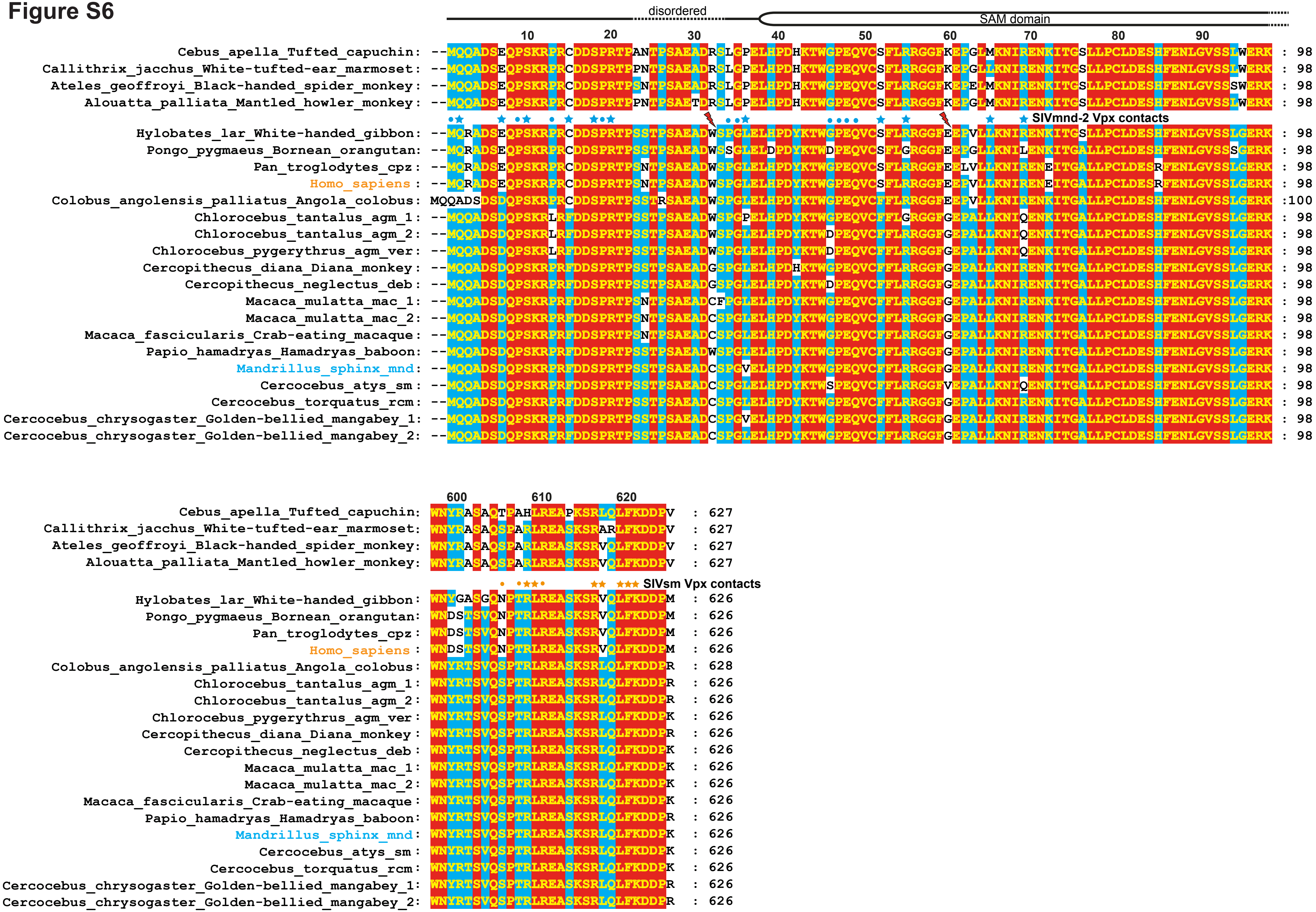

disordered
SAM domain
10 20 30 40 50 60 70 80 90
Cebus_apella_Tufted_capuchin: --MQQADSEQPSKRPRCDDSPRTPANTPSAEADRSLGPELHPDHKTWGPEQVCSFLRRGGFKEPGLMKNIRENKITGSLLPCLDESHFENLGVSSLWERK : 98
Callithrix_jacchus_White-tufted-ear_marmoset: --MQQADSEQPSKRPRCDDSPRTPPNTPSAEADRSLGPELHPDHKTWGPEQVCSFLRRGGFKEPGLLKNIRENKITGSLLPCLDESHFENLGVSSLWERK : 98
Ateles_geoffroyi_Black-handed_spider_monkey: --MQQADSEQPSKRPRCDDSPRTPSNTPSAEADRSLGPELHPDHKTWGPEQVCSFLRRGGFKEPELMKNIRENKITGSLLPCLDESHFENLGVSSSWERK : 98
Alouatta_palliata_Mantled_howler_monkey: --MQQADSEQPSKRPRCDDSPRTPPNTPSAETDRSLGPELHPDHKTWGPEQVCSFLRRGGFKEPGLMKNIRENKITGSLLPCLDESHFENLGVSSLWERK : 98
SIVmnd-2 Vpx contacts
Hylobates_lar_White-handed_gibbon: --MQRADSEQPSKRPRCDDSPRTPSSTPSAEADWSPGLELHPDYKTWGPEQVCSFLRRGGFEEPVLLKNIRENKITGSLLPCLDESHFENLGVSSLGERK : 98
Pongo_pygmaeus_Bornean_orangutan: --MQRADSEQPSKRPRCDDSPRTPSSTPSAEADWSSGLELDPDYKTWDPEQVCSFLGRGGFEEPGLLKNILENKITGALLPCLDESHFENLGVSSSGERK : 98
Pan_troglodytes_cpz: --MQRADSEQPSKRPRCDDSPRTPSNTPSAEADWSPGLELHPDYKTWGPEQVCSFLRRGGFEELVLLKNIRENEITGALLPCLDESRFENLGVSSLGERK : 98
Homo_sapiens: --MQRADSEQPSKRPRCDDSPRTPSNTPSAEADWSPGLELHPDYKTWGPEQVCSFLRRGGFEEPVLLKNIRENEITGALLPCLDESRFENLGVSSLGERK : 98
Colobus_angolensis_palliatus_Angola_colobus: MQQADSDSDQPSKRPRCDDSPRTPSSTRSAEADWSPGLELHPDYKTWGPEQVCFFLRRGGFEEPVLLKNIRENKITGALLPCLDESHFENLGVSSLGERK :100
Chlorocebus_tantalus_agm_1: --MQQADSDQPSKRLRFDDSPRTPSSTPSAEADWSPGPELHPDYKTWGPEQVCFFLGRGGFGEPALLKNIQENKITGALLPCLDESHFENLGVSSLGERK : 98
Chlorocebus_tantalus_agm_2: --MQQADSDQPSKRLRFDDSPRTPSSTPSAEADWSPGLELHPDYKTWDPEQVCFFLRRGGFGEPALLKNIQENKITGALLPCLDESHFENLGVSSLGERK : 98
Chlorocebus_pygerythrus_agm_ver: --MQQADSDQPSKRLRFDDSPRTPSSTPSAEADWSPGLELHPDYKTWDPEQVCFFLRRGGFGEPALLKNIQENKITGALLPCLDESHFENLGVSSLGERK : 98
Cercopithecus_diana_Diana_monkey: --MQQADSDQPSKRPRFDDSPRTPSSTPSAEADGSPGLELHPDHKTWGPEQVCFFLRRGGFGEPALLKNIRENKITGALLPCLDESHFENLGVSSLGERK : 98
Cercopithecus_neglectus_deb: --MQQADSDQPSKRPRFDDSPRTPSSTPSAEADGSPGLELHPDYKTWDPEQVCFFLRRGGFGEPALLKNIRENKITGALLPCLDESHFENLGVSSLGERK : 98
Macaca_mulatta_mac_1: --MQQADSDQPSKRPRFDDSPRTPSNTPSAEADCFPGLELHPDYKTWGPEQVCFFLRRGGFGEPALLKNIRENKITGALLPCLDESHFENLGVSSLGERK : 98
Macaca_mulatta_mac_2: --MQQADSDQPSKRPRFDDSPRTPSNTPSAEADCSPGLELHPDYKTWGPEQVCFFLRRGGFGEPALLKNIRENKITGALLPCLDESHFENLGVSSLGERK : 98
Macaca_fascicularis_Crab-eating_macaque: --MQQADSDQPSKRPRFDDSPRTPSNTPSAEADCSPGLELHPDYKTWGPEQVCFFLRRGGFGEPALLKNIRENKITGALLPCLDESHFENLGVSSLGERK : 98
Papio_hamadryas_Hamadryas_baboon: --MQQADSDQPSKRPRFDDSPRTPSSTPSAEADWSPGLELHPDYKTWGPEQVCFFLRRGGFGEPALLKNIRENKITGALLPCLDESHFENLGVSSLGERK : 98
Mandrillus_sphinx_mnd: --MQQADSDQPSKRPRFDDSPRTPSSTPSAEADCSPGVELHPDYKTWGPEQVCFFLRRGGFGEPALLKNIRENKITGALLPCLDESHFENLGVSSLGERK : 98
Cercocebus_atys_sm: --MQQADSDQPSKRPRFDDSPRTPSSTPSAEADCSPGLELHPDYKTWSPEQVCFFLRRGGFVEPALLKNIQENKITGALLPCLDESHFENLGVSSLGERK : 98
Cercocebus_torquatus_rcm: --MQQADSDQPSKRPRFDDSPRTPSSTPSAEADCSPGLELHPDYKTWGPEQVCFFLRRGGFGEPALLKNIRENKITGALLPCLDESHFENLGVSSLGERK : 98
Cercocebus_chrysogaster_Golden-bellied_mangabey_1: --MQQADSDQPSKRPRFDDSPRTPSSTPSAEADCSPGVELHPDYKTWGPEQVCFFLRRGGFGEPALLKNIRENKITGALLPCLDESHFENLGVSSLGERK : 98
Cercocebus_chrysogaster_Golden-bellied_mangabey_2: --MQQADSDQPSKRPRFDDSPRTPSSTPSAEADCSPGLELHPDYKTWGPEQVCFFLRRGGFGEPALLKNIRENKITGALLPCLDESHFENLGVSSLGERK : 98
600 610 620
Cebus_apella_Tufted_capuchin: WNYRASAQTPAHLREAPKSRLQLFKDDPV : 627
Callithrix_jacchus_White-tufted-ear_marmoset: WNYRASAQSPARLREASKSRARLFKDDPV : 627
Ateles_geoffroyi_Black-handed_spider_monkey: WNYRASAQSPARLREASKSRVQLFKDDPV : 627
Alouatta_palliata_Mantled_howler_monkey: WNYRASAQSPARLREASKSRVQLFKDDPV : 627
SIVsm Vpx contacts
Hylobates_lar_White-handed_gibbon: WNYGASGQNPTRLREASKSRVQLFKDDPM : 626
Pongo_pygmaeus_Bornean_orangutan: WNDSTSVQNPTRLREASKSRVQLFKDDPM : 626
Pan_troglodytes_cpz: WNDSTSVQNPTRLREASKSRVQLFKDDPM : 626
Homo_sapiens: WNDSTSVQNPTRLREASKSRVQLFKDDPM : 626
Colobus_angolensis_palliatus_Angola_colobus: WNYRTSVQSPTRLREASKSRLQLFKDDPR : 628
Chlorocebus_tantalus_agm_1: WNYRTSVQSPTRLREASKSRLQLFKDDPR : 626
Chlorocebus_tantalus_agm_2: WNYRTSVQSPTRLREASKSRLQLFKDDPR : 626
Chlorocebus_pygerythrus_agm_ver: WNYRTSVQSPTRLREASKSRLQLFKDDPK : 626
Cercopithecus_diana_Diana_monkey: WNYRTSVQSPTRLREASKSRLQLFKDDPR : 626
Cercopithecus_neglectus_deb: WNYRTSVQSPTRLREASKSRLQLFKDDPK : 626
Macaca_mulatta_mac_1: WNYRTSVQSPTRLREASKSRLQLFKDDPK : 626
Macaca_mulatta_mac_2: WNYRTSVQSPTRLREASKSRLQLFKDDPK : 626
Macaca_fascicularis_Crab-eating_macaque: WNYRTSVQSPTRLREASKSRLQLFKDDPK : 626
Papio_hamadryas_Hamadryas_baboon: WNYRTSVQSPTRLREASKSRLQLFKDDPR : 626
Mandrillus_sphinx_mnd: WNYRTSVQSPTRLREASKSRLQLFKDDPK : 626
Cercocebus_atys_sm: WNYRTSVQSPTRLREASKSRLQLFKDDPK : 626
Cercocebus_torquatus_rcm: WNYRTSVQSPTRLREASKSRLQLFKDDPK : 626
Cercocebus_chrysogaster_Golden-bellied_mangabey_1: WNYRTSVQSPTRLREASKSRLQLFKDDPR : 626
Cercocebus_chrysogaster_Golden-bellied_mangabey_2: WNYRTSVQSPTRLREASKSRLQLFKDDPR : 626

SUPPLEMENTAL FIGURE LEGENDS

Figure S1, related to Figure 1. Degron fusion proteins. (**A**) Expression of (NLS)-GFP-degron fusion proteins in was assessed *M. dunni* cells by Western blotting. For each construct the top panel shows an anti-Hsp90 blot loading control. In the lower panels an anti-GFP antibody was used to detect the expression level of each degron fusion indicated. (**B**) Primary FACS data for degron assays. Stable cell lines expressing degrons containing $SAMHD1_{mnd}$ residues 1-114 (upper panels, magenta) or 1-37 (lower panels, purple) were transduced with increasing $SIV_{mnd\text{-}2}$ Vpx (left to right) and analysed by flow cytometry.

Figure S2, related to Figure 2. Electron density. Stereo image of ($2F_{obs} - F_c$) refined electron density for DCAF1 (top), Vpx (middle) and SAMHD1 (bottom) contoured at 1 σ. The density is shown as light blue wireframe and the backbone Cα traces of the final refined models as ribbon representation.

Figure S3, related to Figure 2. Vpx conservation of zinc coordination. The protein backbones of (**A**) Vpx from $SIV_{mnd\text{-}2}$ (blue) and (**B**) SIV_{smm} (orange) are shown in cartoon representation, α-helices are labelled and zinc ions are shown as grey spheres. Residues that co-ordinate zinc ions are shown as sticks, the coordinating water molecule in $SIV_{mnd\text{-}2}$ Vpx as a red sphere and co-ordinating bonds as dashed lines.

Figure S4, related to Figure 3. Superposition of DCAF1-CtD-Vpx complexes. (**A**) Overview of structurally aligned Vpx/DCAF1-CtD complexes. The orientation is as in **Figure 3.** Bound Vpx molecules are shown in cartoon representation, $SIV_{mnd\text{-}2}$ Vpx (blue) and SIV_{smm} Vpx

(orange). (**B-E**) Details of Vpx/DCAF1-CtD interactions in the regions boxed in **a**. Residues that make interactions are shown in stick representation, the DCAF1-CtD cartoon is coloured white, in the SIV_{mnd-2} and grey in the SIV_{smm} complexes.

Figure S5, related to Figure 3 and Figure 6. Multiple sequence alignment of HIV/SIV Vpx and Vpr proteins. 90% type-conserved amino acid residues are highlighted in red, 60% type-conserved in cyan, variable regions are boxed and the position of secondary structure elements is displayed between the Vpr and Vpx groupings. Red stars above the alignment indicate zinc-binding side chains. Residues that interact with SAMHD1 degrons are indicated with blue (SIV_{mnd-2}) or orange (SIV_{smm}) stars (side chain) or dots (backbone). Grey stars indicate Vpx residues with side chains that interact with DCAF1. Darker shading is applied to those that are also type-conserved in Vpr. Numbering below is for SIV_{mnd-2} 5440 Vpx.

Figure S6, related to Figure 5 and Figure 6. Multiple sequence alignment of primate SAMHD1 N- and C-terminal regions. 100% type-conserved amino acid residues are highlighted in red, 60% type-conserved in cyan. SAMHD1 residues that interact with the respective Vpx are indicated with blue (SIV_{mnd-2}) or orange (SIV_{smm}) stars (side chain) or dots (backbone). Flashes indicate the highly divergent residues 32 and 60.

Table S1A related to Figure 3. Vpx-DCAF1 Interface residues (1595 Å^2)

Vpx residue	Region	Interaction	DCAF1 residue	Region
A2$_{MC}$	Nt	HB	R1106	WD40 1
E3$_{MC}$	Nt	HB	R1106	WD40 1
E3	Nt	HB	R1106$_{MC}$	WD40 1
E3	Nt	HB	S1102	WD40 1
E3	Nt	HI	R1106, F1107	WD40 1
A5	Nt	HI	F1107, L1119	WD40 1
P6	Nt	HB	Y1131	WD40 1
P6	Nt	HI	Y1131	WD40 1
P6	Nt	HI	M1166, F1170	WD40 2
E7$_{MC}$	Nt	HB	S1168$_{MC}$	WD40 2
E7$_{MC}$	Nt	HB	F1170$_{MC}$	WD40 2
I28	α1	HI	W1156	WD40 2
E31	α1	HI	W1156	WD40 2
L44	α2	HI	A1377	WD40 7
T47	α2	HI	L1378	WD40 7
C48	α2	HI	L1378	WD40 7
H51	α2	HI	L1378	WD40 7
C52	α2	HI	L1378	WD40 7
Y62	α3	HB	D1092	WD40 7/1
Y65	α3	HB	E1091	WD40 7/1
R66	α3	SB	E1093	WD40 7/1
L68	α3	HI	T1114	WD40 1
L69	α3	HI	C1113, T1114	WD40 1
L70	α3	HI	A1377, L1378, M1380	WD40 7
H72	α3	HB	N1135$_{MC}$	WD40 1/2
H72	α3	HI	C1113, T1114	WD40 1
K73	α3	SB	E1093	WD40 7/1
K73	α3	HB	S1094$_{MC}$	WD40 7/1
K73	α3	HI	C1113	WD40 1
M75	α3	HI	W1156	WD40 2
Y76	α3	HB	T1097$_{MC}$	WD40 1
Y76	α3	HB	F1355$_{MC}$	WD40 7
Y76	α3	HI	A1137, T1139	WD40 1/2
Y76	α3	HI	F1330	WD40 6
T77	α3	HI	P1329, F1330	WD40 6
M79	α3	HI	T1155, W1156	WD40 2
Q81	α3	HB	Q1314$_{MC}$	WD40 6
Q81$_{MC}$	α3	HB	R1225	WD40 4
Q81	α3	HI	L1313, P1329	WD40 6

HB – hydrogen bond, HI – hydrophobic interaction, SB – salt bridge MC – mainchain

Table S1B, related to Figure 3. DCAF1-SAMHD1 interface residues (499 Å^2)

DCAF1 residue	Region	Type of contact	SAMHD1 residue	Region
$A1089_{MC}$	WD40 7/1	HB	R14	Nt
N1090	WD40 7/1	HI	R12	Nt
E1091	WD40 7/1	SB	R12	Nt
D1092	WD40 7/1	SB	R14	Nt
T1114, $T1114_{MC}$	WD40 1	HB	Q8	Nt
Q1116	WD40 1	HI	Q8, R12	Nt
N1132	WD40 1/2	HB	D7, $Q8_{MC}$	Nt
$H1134_{MC}$	WD40 1/2	HB	Q8	Nt
N1135	WD40 1/2	HB	$D5_{MC}$	Nt

HB – hydrogen bond, HI – hydrophobic interaction, SB – salt bridge MC – mainchain

Table S1C, related to Figure 3. Vpx-SAMHD1 Interface residues (1334 Å^2)

Vpx residue	Region	Interaction	SAMHD1 residue	Region
P9	Nt	HI	M1, Q2	Nt
$Q10_{MC}$	Nt	HB	$M1_{MC}$, $Q2_{MC}$	Nt
G11	Nt	HI	Q2	Nt
A12	Nt	HI	Q2	Nt
G13	Nt	HB	D7, S10, $S10_{MC}$	Nt
$E14_{MC}$	Nt	HB	D7	Nt
$V15_{MC}$	Nt	HB	D7	Nt
V15	Nt	HI	D7, P9	Nt
L17	α1	HI	P9, P13	Nt
W20	α1	HI	Q8, P9	Nt
N29	α1	HI	V36	Nt
E39	α2	HB	$P47_{MC}$, $E48_{MC}$, $Q49_{MC}$	SAM domain
L41	α2	HI	V36	Nt
F42	α2	HI	L38, Q49, F52	SAM domain
W45	α2	HI	P34	Nt
N46	α2	HB	E48, $E48_{MC}$	SAM domain
V49	α2	HI	R55	SAM domain
E50	α2	SB	R69	SAM domain
H53	α2	HI	R20	Nt
D54	α2	SB	R20	Nt
D54	α2	SB	R55	SAM domain
H56	α2	HI	F15	Nt
Q57	α2/ α3 loop	HB	R20	Nt
$Q57_{MC}$	α2/ α3 loop	HB	$R20_{MC}$	Nt
R58	α2/ α3 loop	HI	F15	Nt
R58	α2/ α3 loop	HB	$F15_{MC}$	Nt
$S59_{MC}$	α2/ α3 loop	HB	$S18_{MC}$	Nt
Y62	α3	HI	F15, S18	Nt
Y65	α3	HI	P13	Nt
Y65	α3	HB	R12	Nt

HB – hydrogen bond, HI – hydrophobic interaction, SB – salt bridge MC – mainchain

SUPPLEMENTAL EXPERIMENTAL PROCEDURES

Degron assay

Degron reporter constructs comprising two copies of Nuclear Localisation Signal (NLS)-EGFP fused to N-terminal sequences from SAMHD1$_{mnd}$ (NLS-EGFP-SAMHD1$_{mnd}$-NtD) were generated by replacing the human SAMHD1-CtD degron sequence in pCMS28-NLS-EGFP-SAMHD1-CtD (Schwefel et al., 2014) with sequences from the N-terminal region of SAMHD1$_{mnd}$. DNA coding for residues 1-114, 1-37, 37-114, 5-114 or 10-114 was amplified by PCR and inserted into the reporter construct using XhoI/EcoRI restriction sites. Point mutations were created by PCR-based site directed mutagenesis. Virus-like particles (VLPs) were generated by co-transfecting 293T cells with pVSVG, pKB4 and pCMS28-NLS-EGFP-SAMHD1 human-CtD or mandrill-NtD, wildtype or mutant (Schwefel et al., 2014). Stable cell lines were produced by transduction of *Mus dunni* cells followed by puromycin selection. Expression of degron constructs was assessed using Western blotting with anti-EGFP antibodies.

The SIV$_{smm}$ Vpx sequence was amplified by PCR from pIRES2-EGFP-Vpx (a gift from Mario Stevenson), and SIV$_{mnd-2}$ Vpx was amplified from the PET49 plasmid used for *E. coli* expression. Sequences were inserted into pENTR/D/TOPO (Invitrogen) and transferred into pLgatewayIeYFP (Gateway LR clonase^{TM} II, Invitrogen) to create bicistronic Vpx-IRES-YFP expression constructs. Point mutations were created by PCR-based site directed mutagenesis. VLPs expressing Vpx-IRES-YFP were generated by co-transfecting 293T cells with pVSV-G, pKB4 and pLgatewayIeYFP-Vpx (wildtype or mutants) (Schwefel et al., 2014). Approximately 18 hours after transfection, cells were washed and sodium butyrate medium (0.02 M sodium butyrate, 10% FCS and 1% penicillin/streptomycin in DMEM) was added for 6 hours before

replacing with fresh media. After a further 15 hours VLPs were harvested from the media by filtration.

Parental *Mus dunni* or stable cell lines expressing degron reporters were seeded at 5×10^4 cells per well in a 24-well plate one day prior to infection. Cells were infected with 2-fold serial dilutions of Vpx-YFP VLPs in the presence of 1μg/mL Polybrene. After 48 hours, cells were harvested and the percentage of EGFP-positive and YFP-positive cells was determined by flow cytometry using a FACSVerse analyser (BD Biosciences).

Protein expression and purification

The nucleotide sequences coding for SIV_{mnd-2} Vpx isolate 5440 (Hu et al., 2003) and amino acid residues 1-114 of $SAMHD1_{mnd}$ (Uniprot ID H6WEA4) were synthesised codon-optimised for *E. coli* (Life Technologies). The open reading frames were inserted into pET-49b and pET-52b (Merck Millipore) expression plasmids respectively using flanking XmaI/NotI restriction sites to generate N-terminally GST-tagged and N-terminally Strep-II-tagged fusion proteins. DCAF1-CtD was cloned and expressed as described previously (Schwefel et al., 2014).

SIV_{mnd-2} Vpx and $SAMHD1_{mnd}$-NtD were expressed in the *E. coli* strain Rosetta 2 (DE3) (Merck Millipore). Bacterial cultures were grown in terrific broth medium in a shaking incubator at 37 °C. Protein expression was induced by the addition of 0.1 mM IPTG at A_{600} = 0.5, then further incubated at 18 °C for 20 hours to express recombinant proteins. Cells were harvested by centrifugation for 20 min at 4,500 xg and 4 °C, the cell pellets resuspended in 30 mL lysis buffer (50 mM Tris-HCl pH 7.8, 500 mM NaCl, 4 mM $MgCl_2$, 0.5 mM TCEP, 1x EDTA-free mini complete protease inhibitors (Roche), 0.1 U/ml Benzonase (Novagen) per pellet of 1 L bacteria culture and stored at -20 °C.

$SAMHD1_{mnd}$-NtD cell suspensions were lysed by disruption using an EmulsiFlex-C5 homogeniser (Avestin). The lysate was cleared by centrifugation for 1 hour at 48,000 xg at 4 °C. All further purification steps were performed at 4 °C or on ice. Lysates were applied to 10 mL StrepTactin column (IBA). The column was washed with 600 mL of Wash buffer (50 mM Tris-HCl pH 7.8, 500 mM NaCl, 4 mM $MgCl_2$, 0.5 mM TCEP) and bound proteins were eluted in wash buffer containing 2.5 mM d-desthiobiotin. Eluted fractions were concentrated to 5 mL and further purified on a Superdex75 gel filtration column (GE Healthcare) equilibrated in Gel filtration buffer (10 mM Tris-HCl pH 7.8, 150 mM NaCl, 4 mM $MgCl_2$, 0.5 mM TCEP). Peak fractions containing $SAMHD1_{mnd}$-NtD were pooled, concentrated to 30 mg/mL, snap-frozen in liquid nitrogen in small aliquots and stored at -80 °C.

Protein complex assembly

Cell suspension from 1 L of GST-SIV_{mnd-2} Vpx was lysed by homogenisation in an EmulsiFlex-C5 (Avestin). The lysate was cleared by centrifugation for 1 h at 48,000 xg at 4 °C. All further purification steps were performed at 4 °C or on ice. 1 mL of glutathione Sepharose (GSH-Sepharose) beads (GE Healthcare) were added to the lysate and incubated for 1 hour on a roller agitator. Beads were pelleted by centrifugation at 4000 xg for 10 min, the supernatant was discarded and the beads washed four times with 50 mL of Wash buffer. For assembly, the GST-SIV_{mnd-2} Vpx bound beads were resuspended in 10 mL wash buffer in a 15 mL Falcon tube. 1 mg of DCAF1-CtD together with an equimolar amount of $SAMHD1_{mnd}$-NtD and 1 mg of HRV-3C protease (GE Healthcare) were added and the tube was incubated overnight on a rolling agitator. Beads were then removed by centrifugation at 4000 xg for 10 min. The supernatant was concentrated to 5 mL and applied to a Superdex200 size exclusion column equilibrated in Gel

filtration buffer. Peak fractions containing the ternary complex were pooled, concentrated to 12 mg/mL, snap-frozen in liquid nitrogen in small aliquots and stored at -80 °C.

Crystallization and data collection

Crystals of the SIV_{mnd-2} Vpx/$SAMHD1_{mnd}$-NtD/DCAF1CtD complex were grown using the hanging drop vapour diffusion method by mixing 1 μL complex at a concentration of 6.34 mg/mL with 1 μL of reservoir solution containing 0.16 M Trisodium Citrate-HCl pH 5.2 and 4% PEG 6000. Drops were equilibrated over a 450 μL reservoir solution at 18 °C. Crystals were adjusted to 25 % glycerol and cryo-cooled in liquid nitrogen. A data set from a single crystal was collected on station I04 at the Diamond synchrotron light source, UK at a wavelength of 0.97965 Å.

Structure solution

Diffraction data were reduced with the program XDS (Kabsch, 2010). The high resolution cut-offwas based on the $CC_{1/2}$ criteria (Karplus and Diederichs, 2012). The structure was solved by molecular replacement with the program Molrep (Vagin and Teplyakov, 2010) using the previously determined DCAF1-CtD structure and a homology model constructed with the previously determined SIV_{smm} Vpx as template (PDB code 4CC9 (Schwefel et al., 2014)). The SAM domain was placed manually into density using the NMR structure of the human SAMHD1 SAM domain as guidance (PDB code 2E8O). Iterative model adjustment using the program Coot (Emsley et al., 2010) combined with positional, real-space, individual b-factor and TLS refinement with the program phenix.refine (Adams et al., 2010) produced a final model for DCAF1-CtD residues 1073-1315, 1327-1392 (chain A), SIV_{mnd-2} Vpx residues 2-86 (chain B)

and SAMHD1$_{mnd}$-NtD residues 1-22, 34-88, 93-109 (chain C) with R(R_{free})-factors of 17.5% (23.1%). 95.8% of all residues fall in the favoured region of the Ramachandran plot with 0.42% outliers. Data collection and refinement statistics are shown in Table 1.

Multiple sequence alignment

Amino acid sequences were aligned using the ClustalW server and adjusted manually. NCBI accession numbers for Vpr sequences: HIV-1 ETH2220 - U46016, HIV-1 WEAU - U21135, HIV-1 pNL4-3 - AF324493, HIV-1 pNL432 - M28355, SIV$_{smm}$ - AF077017, SIV$_{cpzCAM13}$ - AY169968, SIVcpz$_{LB7}$ - DQ373064, SIV$_{mac239}$ - M33262, SIV$_{mac251}$ - M76764, SIV$_{rcm}$ - HM803689, SIV$_{mnd-2}$ - AF367411, SIV$_{agmSAB1}$ - U04005, SIV$_{agmGRI}$ - M66437, SIV$_{agmVER}$ - KF741091, SIV$_{agmTAN1}$ - U58991; for Vpx sequences: HIV-2A - M30502, HIV-2B - U27200, SIV$_{smm}$ - AF077017, SIV$_{mac251}$ - M76764, SIV$_{mac239}$ - M33262, SIV$_{rcmNG}$ - AF349680, SIV$_{rcmCAM}$ - HM803689, SIV$_{rcmGAB1}$ - AF382829, SIV$_{drl-1}$ - AY159321, SIV$_{mnd-2CM16}$ - AF367411, SIV$_{mnd-25440}$ - AY159322, SIV$_{mnd-2M14}$ - AF328295; for SAMHD1 sequences: *Cebus paella* - JN936910, *Callithrix jacchus* - JN936906, *Ateles geoffroyi* - JN936911, *Alouatta palliata* - JN936912, *Hylobates lar* - JN936889, *Pongo pygmaeus* - JN936888, *Pan troglodytes* - JN936887, *Homo sapiens* - BC036450, *Colobus angolensis* - JN936905, *Chlorocebus tantalus 1* - JN936891, *Chlorocebus tantalus 2* - JN936892, *Chlorocebus pygerythrus* - JQ231137, *Cercopithecus Diana* - JN936902, *Cercopithecus neglectus* - JQ231141, *Macaca mulatta 1* - JN936894, *Macaca mulatta 2* - JN936895, *Macaca fascicularis* - JN936893, *Papio hamadryas* - JN936890, *Mandrillus sphinx* - JN936897, *Cercocebus atys* - JQ231132, *Cercocebus torquatus* - JQ231133, *Cercocebus chrysogaster 1* - JN936898, *Cercocebus chrysogaster 2* - JN936899.

SUPPLEMENTAL REFERENCES

Adams, P.D., Afonine, P.V., Bunkoczi, G., Chen, V.B., Davis, I.W., Echols, N., Headd, J.J., Hung, L.W., Kapral, G.J., Grosse-Kunstleve, R.W., *et al*. (2010). PHENIX: a comprehensive Python-based system for macromolecular structure solution. Acta crystallographica. Section D, Biological crystallography *66*, 213-221.

Emsley, P., Lohkamp, B., Scott, W.G., and Cowtan, K. (2010). Features and development of Coot. Acta crystallographica. Section D, Biological crystallography *66*, 486-501.

Hu, J., Switzer, W.M., Foley, B.T., Robertson, D.L., Goeken, R.M., Korber, B.T., Hirsch, V.M., and Beer, B.E. (2003). Characterization and comparison of recombinant simian immunodeficiency virus from drill (Mandrillus leucophaeus) and mandrill (Mandrillus sphinx) isolates. J Virol *77*, 4867-4880.

Kabsch, W. (2010). Xds. Acta crystallographica. Section D, Biological crystallography *66*, 125-132.

Karplus, P.A., and Diederichs, K. (2012). Linking crystallographic model and data quality. Science *336*, 1030-1033.

Vagin, A., and Teplyakov, A. (2010). Molecular replacement with MOLREP. Acta Crystallographica Section D *66*, 22-25.
