# Supplementary material for: Molecular Determinants for Recognition of Divergent SAMHD1 Proteins by the Lentiviral Accessory Protein Vpx
Source: Cell Host Microbe. 2015 Apr 8;17(4):489–99. doi: 10.1016/j.chom.2015.03.004 (PMC4400269; doi:10.1016/j.chom.2015.03.004)
Supplement: Document S2. Article plus Supplemental Information [file mmc2.pdf]

Article

Cell Host & Microbe

Molecular Determinants for Recognition of Divergent SAMHD1 Proteins by the Lentiviral Accessory Protein Vpx

Graphical Abstract

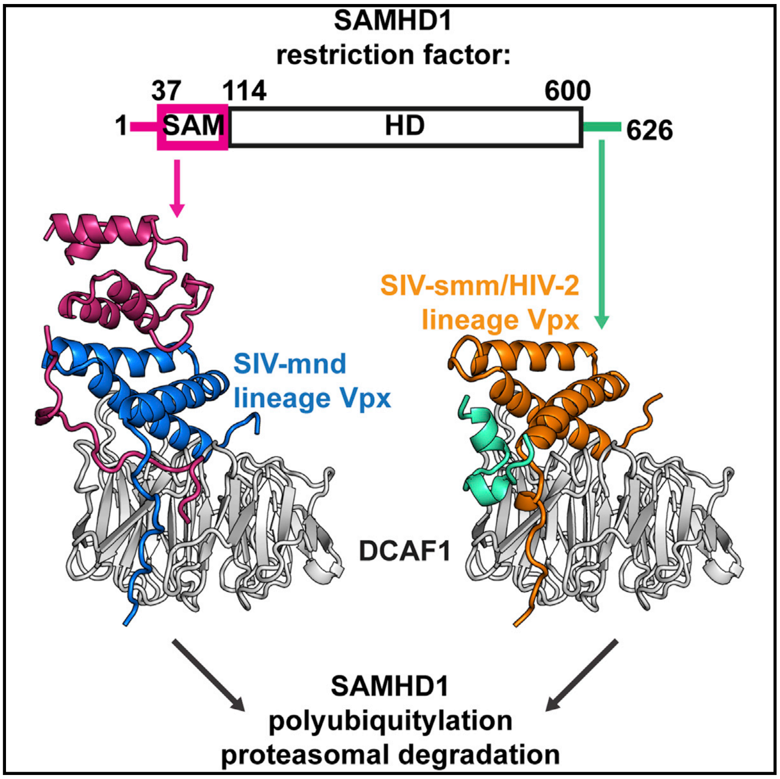

Authors

David Schwefel,
Virginie C. Boucherit, ...,
Kate N. Bishop, Ian A. Taylor

Correspondence

kbishop@nimr.mrc.ac.uk (K.N.B.),
itaylor@nimr.mrc.ac.uk (I.A.T.)

In Brief

Vpx of HIV-2 and SIV target the restriction factor SAMHD1 for degradation using the host ubiquitin ligase substrate receptor DCAF1. Schwefel et al. present the ternary complex of Vpx_{mnd-2} with DCAF1 and the SAMHD1 N terminus and reveal how Vpx subverts host cell defenses by employing differing strategies to recruit SAMHD1 for degradation.

Highlights

- The crystal structure of SIV_{mnd-2} Vpx complexed with SAMHD1 and DCAF1
- Comparisons to SIV_{smm} Vpx reveal determinants for N- versus C-terminal SAMHD1 binding
- Vpx and Vpr use conserved determinants to bind to DCAF1

Accession Numbers

5aja

Schwefel et al., 2015, Cell Host & Microbe *17*, 489–499
April 8, 2015 ©2015 The Authors
http://dx.doi.org/10.1016/j.chom.2015.03.004

Cell Host & Microbe
Article

CellPress

Molecular Determinants for Recognition of Divergent SAMHD1 Proteins by the Lentiviral Accessory Protein Vpx

David Schwefel,[1] Virginie C. Boucherit,[2] Evangelos Christodoulou,[1] Philip A. Walker,[1] Jonathan P. Stoye,[2,3] Kate N. Bishop,[2,*] and Ian A. Taylor[1,*]
[1]Division of Molecular Structure
[2]Division of Virology
MRC National Institute for Medical Research, The Ridgeway, Mill Hill, London NW7 1AA, UK
[3]Faculty of Medicine, Imperial College London, London SW7 2AZ, UK
*Correspondence: kbishop@nimr.mrc.ac.uk (K.N.B.), itaylor@nimr.mrc.ac.uk (I.A.T.)
http://dx.doi.org/10.1016/j.chom.2015.03.004
This is an open access article under the CC BY license (http://creativecommons.org/licenses/by/4.0/).

SUMMARY

The SAMHD1 triphosphohydrolase inhibits HIV-1 infection of myeloid and resting T cells by depleting dNTPs. To overcome SAMHD1, HIV-2 and some SIVs encode either of two lineages of the accessory protein Vpx that bind the SAMHD1 N or C terminus and redirect the host cullin-4 ubiquitin ligase to target SAMHD1 for proteasomal degradation. We present the ternary complex of Vpx from SIV that infects mandrills (SIV_{mnd-2}) with the cullin-4 substrate receptor, DCAF1, and N-terminal and SAM domains from mandrill SAMHD1. The structure reveals details of Vpx lineage-specific targeting of SAMHD1 N-terminal "degron" sequences. Comparison with Vpx from SIV that infects sooty mangabeys (SIV_{smm}) complexed with SAMHD1-DCAF1 identifies molecular determinants directing Vpx lineages to N- or C-terminal SAMHD1 sequences. Inspection of the Vpx-DCAF1 interface also reveals conservation of Vpx with the evolutionally related HIV-1/SIV accessory protein Vpr. These data suggest a unified model for how Vpx and Vpr exploit DCAF1 to promote viral replication.

INTRODUCTION

Host cell restriction factors are the first line of cellular defense against retrovirus infection. Successful viral evasion of these adaptive hurdles is essential for productive infection of new hosts and has occurred during multiple cross-species transmission events such as those that led to the emergence of pandemic human immunodeficiency virus-1 (HIV-1) (Hatziioannou and Bieniasz, 2011; Hatziioannou et al., 2014; Sharp and Hahn, 2011). Restriction factors are often interferon induced and inhibit distinct stages of the viral replication cycle (Towers and Noursadeghi, 2014; Wolf and Goff, 2008). Important examples are the TRIM5α, APOBEC3 family, and Tetherin proteins, which interfere with retroviral uncoating, reverse transcription, and budding processes, respectively (Neil et al., 2008; Sheehy et al., 2002; Stremlau et al., 2004). The sterile alpha motif domain and histidine-aspartate domain-containing protein 1 (SAMHD1) was first identified as a disease gene associated with the rare infantile encephalopathy Aicardi-Goutières syndrome (Rice et al., 2009). More recently, SAMHD1 was shown to be a factor that restricts HIV-1 replication in non-dividing myeloid-lineage cells and resting T cells (Baldauf et al., 2012; Berger et al., 2011; Descours et al., 2012; Hrecka et al., 2011; Laguette et al., 2011). SAMHD1 is a dGTP/GTP-activated deoxynucleotide (dNTP) triphosphohydrolase (Amie et al., 2013; Goldstone et al., 2011; Ji et al., 2013; Miazzi et al., 2014; Powell et al., 2011; Zhu et al., 2013) involved in balancing cellular dNTP pools (Franzolin et al., 2013) and regulated by Cyclin A2/CDK1-dependent phosphorylation (Cribier et al., 2013; Kretschmer et al., 2015; Pauls et al., 2014; White et al., 2013). It is proposed that SAMHD1 restriction results from this triphosphohydrolase activity by reducing the cellular dNTP concentration to a level insufficient for the viral reverse transcriptase to function (Kim et al., 2012; Lahouassa et al., 2012; Rehwinkel et al., 2013; St Gelais et al., 2012). An alternative mechanism of SAMHD1 HIV-1 restriction requiring a putative nuclease activity as also been reported, but the nature of the polynucleotide substrate is disputed (Beloglazova et al., 2013; Ryoo et al., 2014; Tüngler et al., 2013).

A hallmark of most retrovirus restriction factors is the existence of viral antagonists in the form of accessory proteins (Malim and Bieniasz, 2012; Strebel, 2013). A common mechanism of action of accessory proteins is the subversion of host cell protein degradation pathways (Strebel, 2013). In particular, the host cell's Cullin-RING-type E3 ubiquitin ligases are often engaged by viral accessory proteins to induce restriction factor poly-ubiquitylation to direct proteasomal degradation (Barry and Früh, 2006). Cullin-RING ubiquitin ligases consist of a central Cullin scaffold protein, a catalytic RING subunit, and varying substrate receptors (Zimmerman et al., 2010). Owing to their modular architecture, Cullin-RING ligases allow for specific placement of a large number of substrates in the ubiquitylation zone of the catalytic subunit for efficient poly-ubiquitylation (Fischer et al., 2011; Zimmerman et al., 2010). Simian immunodeficiency virus (SIV) and HIV accessory proteins exploit these

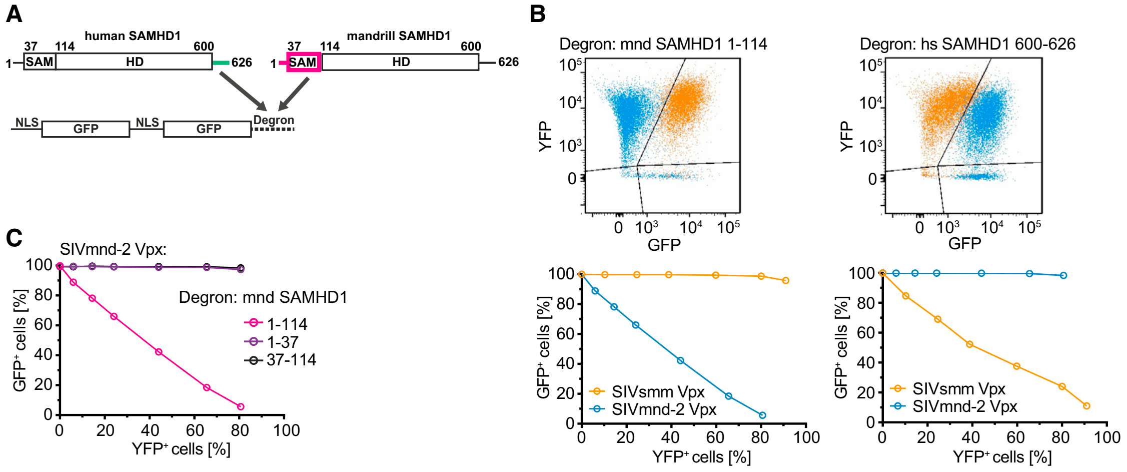

Figure 1. The Mandrill SAMHD1 Degron Comprises the N-Terminal and SAM Domains

(A) Schematic of the human and mandrill SAMHD1 and the tandem NLS-EGFP degron fusion protein construct employed in fluorescent degron assays. The C- and N-terminal degron regions in SAMHD1 are highlighted in green and magenta, respectively.

(B) Stable cell lines expressing either the mandrill (mnd) SAMHD1 1–114 degron (left) or human (hs) SAMHD1 600–626 degron (right) were transduced with increasing titers of particles carrying either SIV_{smm} Vpx (orange) or $SIV_{mnd\text{-}2}$ Vpx (blue) together with YFP. The level of degron (EGFP) and Vpx (YFP) expression was measured by flow cytometry. The upper panels are example FACS plots illustrating the populations of cells observed: untransduced cells (bottom right quadrant), cells transduced with a Vpx protein that can (top left quadrant) or cannot (top right quadrant) induce degradation of the degron reporter construct. The lower panels are quantification of degron reporter expression with increasing Vpx expression, demonstrating differential Vpx recognition of degron sequences.

(C) Quantification of degron reporter expression in stable cells lines expressing constructs containing the indicated N-terminal regions of $SAMHD1_{mnd}$ after transduction with increasing amounts of $SIV_{mnd\text{-}2}$ Vpx; see also Figure S1.

characteristics by modifying the specificity of Cullin-RING substrate receptors (e.g., HIV-1 Vif and Vpu redirect cullin-1 and cullin-5 receptor specificity to induce APOBEC3 and Tetherin restriction factor downregulation, respectively) (Guo et al., 2014; Mitchell et al., 2009). Vpr and Vpx from HIV and SIVs target the cullin-4 ligase substrate receptor DDB1- and CUL4-associated factor 1 (DCAF1, also known as VprBP) from their host (Bergamaschi et al., 2009; Hrecka et al., 2007; Le Rouzic et al., 2007; Srivastava et al., 2008).

Restriction factors and accessory proteins are engaged in an evolutionary "molecular arms race" consisting of multiple rounds of host adaptation, virus counteraction, and host re-adaptation, resulting in accumulation of amino acid changes in restriction factor-accessory protein interaction interfaces (Daugherty and Malik, 2012). In the lentiviral Vpx/Vpr accessory proteins, analyses of positively selected residues and subsequent functional studies have demonstrated the occurrence of several significant specificity changes during adaptation to primate hosts (Laguette et al., 2012; Lim et al., 2012). It is proposed that originally a subset of Vpr proteins acquired the capability to induce cullin-4/DCAF1-dependent proteasomal degradation of SAMHD1. These SAMHD1-degrading Vpr proteins are found in the SIV_{syk} (SIV that infects Sykes' monkey), SIV_{deb} (De Brazza's monkey), and SIV_{agm} (African green monkey) lineages. Later in its evolutionary history, a Vpr gene duplication or recombination event gave rise to Vpx, which completely took over the anti-SAMHD1 functionality and produced viruses that contain both Vpx and Vpr genes (Lim et al., 2012). In these adaptation events, the mode of SAMHD1 binding seems to have toggled between recognition sequences at the SAMHD1 N terminus and others located at the C terminus. Vpx from HIV-2, SIV_{smm} (sooty mangabey) and SIV_{mac} (macaque) all target the SAMHD1 C terminus, whereas $SIV_{mnd\text{-}2}$ (mandrill) and SIV_{rcm} (red-capped mangabey) Vpx induce SAMHD1 degradation dependent on the N-terminal 110 residues. The SAMHD1-degrading Vpr proteins from SIV_{syk}, SIV_{deb}, and SIV_{agm} have varying specificities for the SAMHD1 N or C terminus or even target both (Fregoso et al., 2013; Spragg and Emerman, 2013).

Here, we report the crystal structure of the ternary complex of $SIV_{mnd\text{-}2}$ Vpx bound to the N-terminal region and SAM domain of mandrill SAMHD1 ($SAMHD1_{mnd}$-NtD) and the WD40 domain of the cullin-4 substrate receptor DCAF1 (DCAF1-CtD). This structure reveals unanticipated complexity in Vpx-mediated targeting of SAMHD1. Comparison to the recently determined structure of a SIV_{smm} Vpx/SAMHD1-CtD/DCAF1-CtD (Schwefel et al., 2014) complex provides the molecular details of the structural determinants for N-terminal versus C-terminal SAMHD1 specificity and a plausible scenario for the events occurring during evolutionary change.

RESULTS

Identifying the Mandrill SAMHD1 Degron

To locate the Vpx-DCAF1 recognition sequence within the $SAMHD1_{mnd}$ N terminus, a cell-based EGFP-degron reporter degradation assay was employed (Schwefel et al., 2014). Degron fusion proteins (Figure 1A) were constructed comprising two copies of EGFP with a nuclear localization signal (NLS) fused to either the N-terminal 114 residues of $SAMHD1_{mnd}$ ($SAMHD1_{mnd}$-NtD) or a control C-terminal region of human SAMHD1, residues 600–626, ($SAMHD1_{hs}$-CtD), which is targeted for degradation by Vpx from SIV_{smm} (Schwefel et al., 2014). Stable cell lines expressing each reporter construct were produced and expression levels of degron fusion proteins

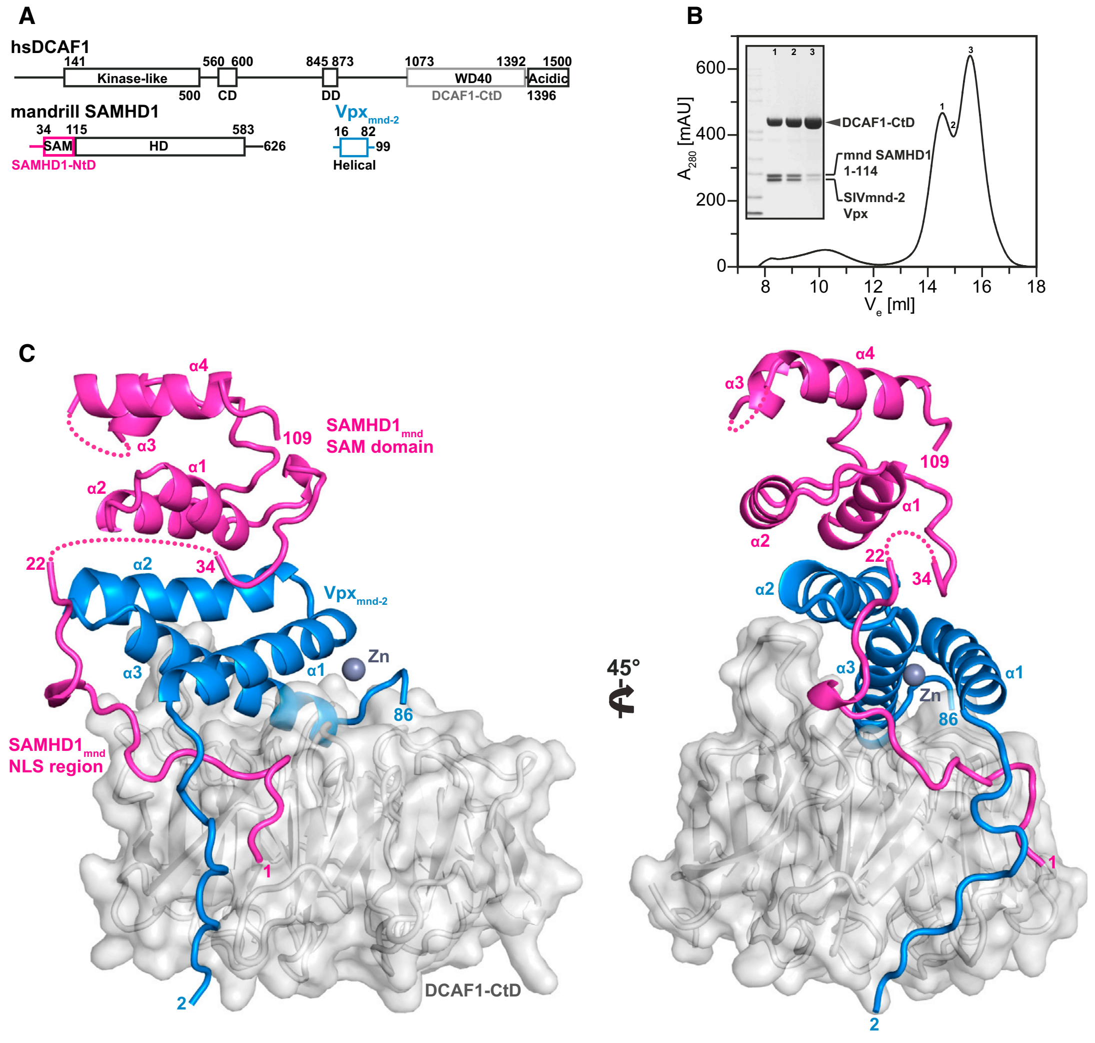

Figure 2. Structure of the SIV$_{mnd\text{-}2}$ Vpx/DCAF1-CtD/SAMHD1$_{mnd}$-NtD Complex

(A) Schematic of components of the protein complex.

(B) Analytical gel filtration of the in vitro-assembled ternary protein complex consisting of SIV$_{mnd\text{-}2}$ Vpx, DCAF1-CtD and SAMHD1$_{mnd}$-NtD (Peak-1). The inset shows SDS-PAGE analysis of the indicated peak fractions.

(C) Structure of the ternary complex. The backbone for each protein is shown in cartoon representation, SIV$_{mnd\text{-}2}$ Vpx (blue), DCAF1-CtD (gray), and SAMHD1$_{mnd}$-NtD (magenta). See also Figure S2. The solvent-accessible surface is also shown for DCAF1-CtD, and a zinc ion co-ordinated by Vpx is displayed as a gray sphere, see also Figure S3. For SIV$_{mnd\text{-}2}$ Vpx and SAMHD1$_{mnd}$-NtD, residue numbers at chain termini are indicated and secondary structure elements labeled.

were confirmed by western blot (Figure S1). Cells were then transduced with increasing amounts of a bicistronic IRES vector expressing Vpx from SIV$_{mnd\text{-}2}$ or SIV$_{smm}$ and YFP, and 48 hr later, the level of degron reporter (EGFP) and Vpx (YFP) expression was measured by flow cytometry. These data show that SIV$_{mnd\text{-}2}$ Vpx induces degradation of the SAMHD1$_{mnd}$-NtD reporter construct but not EGFP-SAMHD1$_{hs}$-CtD (Figure 1B, left panel). In contrast, SIV$_{smm}$ Vpx induces degradation of the SAMHD1$_{hs}$-CtD reporter but not SAMHD1$_{mnd}$-NtD (Figure 1B, right panel). To further delineate the SIV$_{mnd\text{-}2}$ SAMHD1 binding determinants, reporter constructs containing residues 1–37 and 37–114 of SAMHD1$_{mnd}$ were prepared. Residues 1–37 comprise an N-terminal disordered sequence containing a NLS and 37–114 constitutes the SAM domain (cp. PDB: 2E8O). However, neither fragment alone was sufficient to induce degradation of the reporter construct (Figure 1C), demonstrating that both the N-terminal NLS region and SAM domain are required for SIV$_{mnd\text{-}2}$ Vpx/DCAF1-mediated degradation.

Structure of the SIV$_{mnd\text{-}2}$ Vpx/SAMHD$_{mnd}$-NtD/DCAF1-CtD Ternary Complex

To gain further insights into molecular recognition in the SIV$_{mnd\text{-}2}$ Vpx system, SIV$_{mnd\text{-}2}$ Vpx, SAMHD1$_{mnd}$-NtD, and DCAF1-CtD (Figure 2A) were assembled in vitro using recombinant proteins and the ternary complex purified by size-exclusion chromatography (Figure 2B). The protein complex crystallized in space group P6$_5$22, the crystals diffracted to 2.8 Å resolution, and the structure was solved by molecular replacement using the

Table 1. X-Ray Data Collection and Refinement Statistics

	mnd-8498
Data collection	
Space group	$P6_522$
Cell dimensions: *a*, *b*, *c* (Å)	102.04, 102.04, 265.09
Cell dimensions: α, β, γ (°)	90, 90, 120
Resolution (Å)	30 (2.81)[1] – 2.65
R_{sym} or R_{merge} (%)	7.9 (145.3)
$CC_{1/2}$[2]	99.9 (59.9)
$I/\sigma I$	20.70 (1.63)
Completeness (%)	99.6 (99.3)
Redundancy	9.4 (9.7)
Refinement	
Resolution (Å)	30 – 2.65
No. reflections	24,554
R_{work}/R_{free}	17.5/23.1
No. atoms: Protein	3,817
No. atoms: zinc ion	1
No. atoms: water	2
B factors $(Å)^2$:	
B factors $(Å)^2$: protein	87.6
B factors $(Å)^2$: zinc ion	105.1
B factors $(Å)^2$: water	71.15
rms deviations: bond lengths (Å)	0.008
rms deviations: bond angles (°)	1.183

[1]Values in parentheses are for highest-resolution shell. A single crystal was used for data collection.
[2]High-resolution cutoff based on criteria according to Karplus and Diederichs (2012).

SIV_{smm} Vpx/SAMHD1$_{hs}$-CtD/DCAF1-CtD structure (Schwefel et al., 2014) as a search model. Details of the data collection, structure determination, map quality, and refinement are presented in Table 1 and Figure S2.

DCAF1-CtD consists of a typical WD40-repeat seven-bladed β-propeller fold approximately 45 Å diameter and 25 Å in height (Figure 2C). SIV_{mnd-2} Vpx comprises an N-terminal extended arm that precedes three α helices. The side chains of amino acid residues H35 at the C terminus of helix α1 together with H78 and C83 at the C terminus of helix α3 constitute a zinc-binding motif that links the helices together, similar to that observed in SIV_{smm} Vpx (Schwefel et al., 2014). However, in contrast the fourth zinc coordinating ligand in SIV_{mnd-2} Vpx is a water molecule rather than an additional cysteine C-terminal to C83 as occurs in SIV_{smm} Vpx (Figure S3). In the structure, two sections of the SAMHD1$_{mnd}$-NtD are visible. Residues 1–22 are in an extended conformation, and residues 34-109 fold into the SAM domain, which consists of four α helices packed together by interaction of inward-facing hydrophobic amino acid side chains. The ternary complex is built up by three major protein interaction interfaces. These are (i) a Vpx-DCAF1 interface on top and side of the DCAF1 propeller, (ii) a combined interface between the N-terminal extended segment of SAMHD1-NtD, DCAF1 and the Vpx on the side of DCAF1, and (iii) contacts between the SAM domain and Vpx on top of DCAF1.

The DCAF1 Binding Mode Is Conserved in Vpx Proteins

The extensive Vpx-DCAF1 interface comprises four contact regions with a total surface area of 1,600 Å^2 (Figure 3; Table S1A). The first region comprises the C-terminal section of α2 together with first half of α3 of Vpx. On one face, apolar amino acid side chains from both α2 and α3 pack against a cluster of hydrophobic amino acids located in the "tall" loop that connects β2 to β3 in WD40 repeat 7 on the upper surface of DCAF1-CtD (Figure 3B, upper panel). On the other face, a network of hydrogen bonds and salt bridges connects Vpx residues Y62, Y65, R66, and K73 at the N terminus of α3 to acidic side chains E1191, D1092, and E1193 in the "acidic" loop of DCAF1 that intersperses WD40 repeats 7 and 1 (acidic loop, Figure 3B, lower panel). A second region of interaction involves the carboxy terminus of Vpx α3, which packs into a radial groove on the top side of DCAF1 lined by amino acids from WD40 repeats 1, 2, 4, 6, and 7. Interactions here include both the packing of hydrophobic residues as well as specific hydrogen bonding between the side-chains of Vpx Y76 and Q81 and the DCAF1 protein backbone (Figure 3C). The third binding interface involves residues at the extreme amino terminal of the Vpx N-terminal extended arm that inserts into a cavity between WD40 repeats 1 and 2 on the underside of DCAF1. Here, Vpx A2, E3 and E7 make main chain and side chain hydrogen-bonds to DCAF1 residues S1102, R1106, Y1131, and S1168 while the interposing Vpx residues A5 and P6 project into the hydrophobic cavity (Figure 3D). The remaining site of interaction involves contacts between residues in the WD40 repeat 2 of DCAF1 and residues in both Vpx α1 and α3. These include hydrophobic interactions between DCAF1 T1155 and W1156 and apolar side chains displayed on the underside of Vpx α1 and α3 and Vpx H72 on α3 that makes a hydrogen bond with mainchain carbonyl of N1135 of DCAF1 (Figure 3E).

Structural alignment of the DCAF1-CtD and Vpx molecules in the SIV_{mnd-2} and SIV_{smm} complexes (Schwefel et al., 2014) reveals that in both instances they adopt very similar conformations. The root-mean-square deviations (RMSDs) are 0.65 Å (305 aligned residues) for the DCAF1-CtD molecules and 1.24 Å for Vpx (80 aligned residues) (Figure S4A). Moreover, detailed comparison of SIV_{mnd-2} and SIV_{smm} Vpx amino acid side chains that contact DCAF1 shows that nearly all are at least type-conserved (Figures S4B–S4E). A notable exception is T47 in α2 that is exchanged to arginine in SIV_{smm} Vpx and is concomitant with a small movement of α2 and differences in conformation of the interacting DCAF1 tall loop between the two complexes (Figure S4B, upper panel). However, residues on the upper face of α2 also interact with the SAMHD1$_{mnd}$ SAM domain (see below) so the cause of the α2 shift is unclear. Therefore, given that the mode of the N-terminal arm - α2 - α3 Vpx association with DCAF1 is a common feature of SIV_{mnd-2} and SIV_{smm} complexes and that nearly all the DCAF1-interacting residues are type-conserved in HIV-2/SIV Vpx proteins (Figure S5), it is likely that the same principles of DCAF1 binding apply to both the SIV_{mnd-2} and SIV_{smm} Vpx lineages.

A Combined SIV_{mnd-2} Vpx/SAMHD1$_{mnd}$(1-22)/DCAF1-CtD Ternary Interface

In the complex, the N-terminal NLS region (residues 1–22) of SAMHD1$_{mnd}$ comprises an extended chain that binds to the

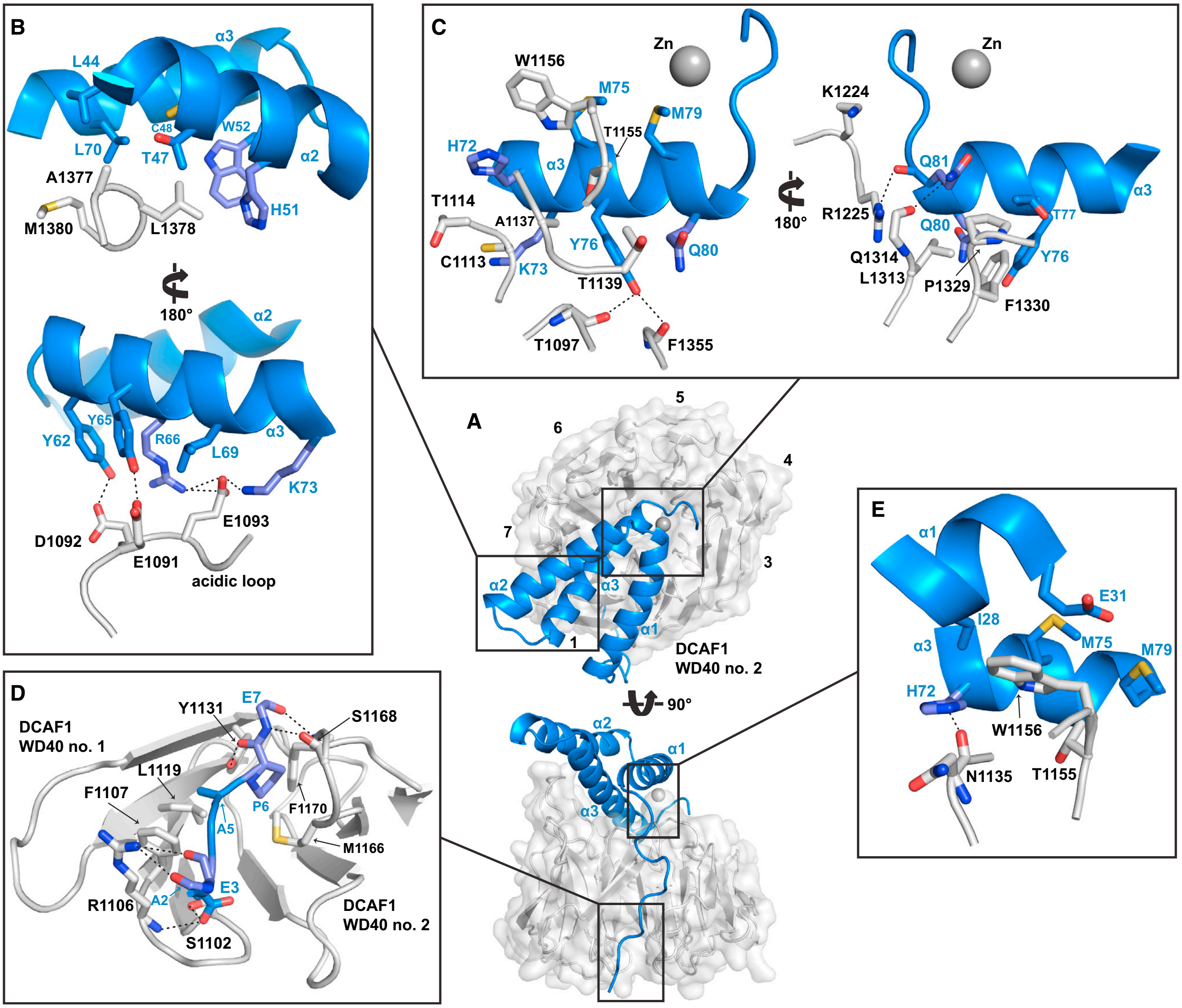

Figure 3. The SIV_{mnd-2} Vpx/DCAF1-CtD Interaction Interface

(A) Overview of Vpx/DCAF1-CtD interactions. Molecules are in the same representation as Figure 2, the WD40 β-propeller repeats of DCAF1-CtD are numbered.
(B–E) Details of Vpx/DCAF1-CtD interactions in the regions boxed in (A). Residues that make interactions are shown in stick representation, dashed lines indicate hydrogen bonds and salt bridges, see also Figure S4 and Table S1.

edge of DCAF1 in an interface that buries 500 $Å^2$ of solvent accessible surface (Figure 4A; Table S1B). Here, residues D5, D7, and Q8 make extensive sidechain and mainchain hydrogen bonds to residues in the interspersing loops of DCAF1 WD40 repeat-1. In addition, SAMHD1 residues R12 and R14, which are part of the SAMHD1 NLS (Brandariz-Nuñez et al., 2012; Hofmann et al., 2012), make salt bridges to E1091 and D1092 in the DCAF1 acidic loop. The ternary interaction is completed by a 750 $Å^2$ $SAMHD1_{mnd}$-SIV_{mnd-2} Vpx interface that includes multiple interactions with Vpx residues from the N-terminal arm, α1, and the α2-α3 loop (Figure 4B; Table S1C). One feature of this interface is a "trapped" region where the Vpx N-terminal arm wraps over SAMHD1 packing it against DCAF1. Within the trapped region, the main chains of SAMHD1 and Vpx interact through hydrogen bonding between E10, G13, and E14 of Vpx and Q2 and S10 of SAMHD1. In addition, the main chain of Vpx G13, and E14 also make interactions with the SAMHD1 D7 and S10 side chains. C-terminal to the trapped region there are further interactions where Vpx V15, L17, and W20 create a hydrophobic pocket at the N terminus of α1 that SAMHD1 P9 inserts into. Additionally, SAMHD1 NLS residue R12 makes a hydrogen bond to Vpx Y65, SAMHD1 F15 stacks with Vpx R58, and SAMHD1 R20 makes an electrostatic interaction with Vpx D54 (Figure 4B).

The functional importance of the Vpx trapping of the SAMHD1 N-terminal region was revealed in an EGFP-degron fusion protein degradation assay. Figure 4C shows that SIVmnd-2 Vpx induces efficient degradation of constructs containing SAMHD1 amino acids 1–114 and 5–114. In contrast, EGFP-SAMHD1 (10–114), which lacks D7 and Q8 that make hydrogen bonds to

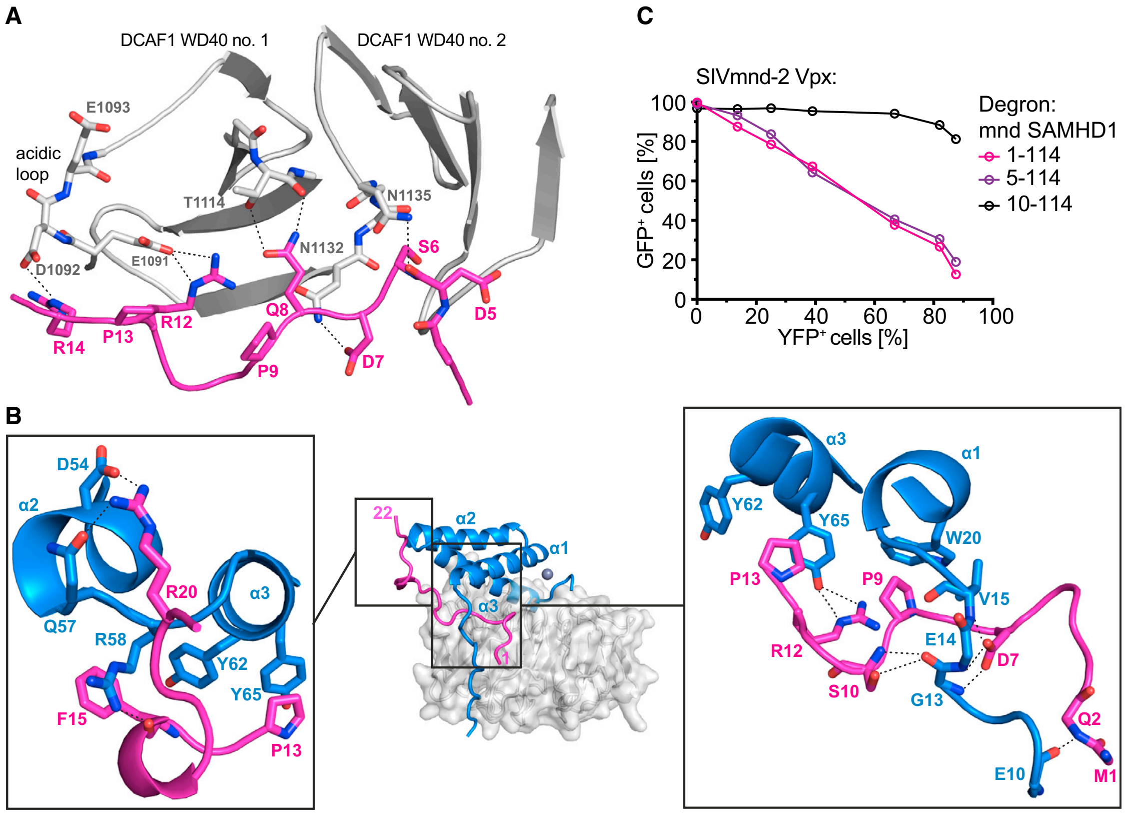

Figure 4. The Combined SIV_{mnd-2} Vpx/DCAF1-CtD/$SAMHD1_{mnd}$-NtD Ternary Interface

(A) Interface of DCAF1-CtD and $SAMHD1_{mnd}$ NLS region. β strands from WD40 repeat-1 and -2 of DCAF1-CtD are shown in gray cartoon representation. The N-terminal NLS region of $SAMHD1_{mnd}$ is shown in magenta. Residues making contacts at the interface are shown in stick representation with hydrogen bonding and salt bridge interactions displayed as dashed lines.

(B) The Vpx-$SAMHD1_{mnd}$ NLS region interface. The ternary complex is displayed (central) in the same orientation and representation as Figure 2. Details of SAMHD1-Vpx interactions within the boxed region are shown left and right. Residues in SAMHD1 (magenta) and Vpx (blue) making contacts at the interface are shown in stick representation with hydrogen bonding and salt bridge interactions displayed as dashed lines.

(C) Quantification of reporter expression in stable cell lines containing degron constructs with the indicated N-terminal truncations of $SAMHD1_{mnd}$. Cells were transduced with increasing titers of particles expressing SIV_{mnd-2} Vpx together with YFP and degron reporter expression (EGFP) measured by flow cytometry.

Vpx and DCAF1 within the trapped region, is not degraded by Vpx indicating the requirement of the trapping interaction for degron activity.

The SIV_{mnd-2} Vpx/ $SAMHD1_{mnd}$ SAM Domain Interface

In addition to the ternary interface that "traps" the $SAMHD1_{mnd}$ N terminus within the complex a further DCAF1-independent interaction between the SAM domain of $SAMHD1_{mnd}$-NtD and SIV_{mnd-2} Vpx is also observed. This interface has a buried surface area of 580 $Å^2$ and comprises residues that project upward from Vpx α2 that contact SAMHD1 residues N-terminal to the SAM domain helix 1 as well as those on the underside of the SAM domain in helices 1 and 2 (Figure 5A). The center of the interface comprises a hydrophobic core created by Vpx residues P38, L41, F42, W45, and V49 and SAMHD1 residues P34, V36, L38, and F52 (Figure 5A, upper panel). In addition to this hydrophobic patch, there are electrostatic interactions between SAMHD1 R55 and Vpx D54 (Figure 5A, upper panel), SAMHD1 R69 and Vpx E50 (Figure 5A, lower panel), as well as hydrogen bonding from Vpx E39 to the backbone of the first turn of SAM domain helix 1 (Figure 5B, lower panel). The contribution of residues in the SAM domain-Vpx interface to degron function was assessed by introduction of alanine mutations at SAMHD1 residues R55 and R69 in the EGFP-fusion reporter. Both residues make extensive electrostatic interactions at the interface, and the alanine substitutions result in a loss of reporter degradation (Figure 5B). R69 is also among a group of residues in the N-terminal region of SAMHD1 that have undergone positive evolutionary selection (Lim et al., 2012) indicative of an interaction hotspot between restriction factor and viral antagonist, further underlining the importance of Vpx-SAM domain contacts for induction of SAMHD1 proteasomal degradation.

Comparison of the sequences of human and mandrill SAMHD1-NtD regions reveals 13 amino-acid changes, nine in the SAM domain and four in the NLS region. Of these, only two make side chain contacts in the mandrill ternary complex, F15 in the NLS region and F52 in the SAM domain. In human SAMHD1, these residues are replaced by non-conservative cysteine and serine substitutions (Figure S6). In the mandrill complex, both phenylalanine residues make interactions, F15 stacks against the side chain of R58 in the Vpx α2-α3 loop (Figure 4B, left panel), and F52 is central to the hydrophobic SAM

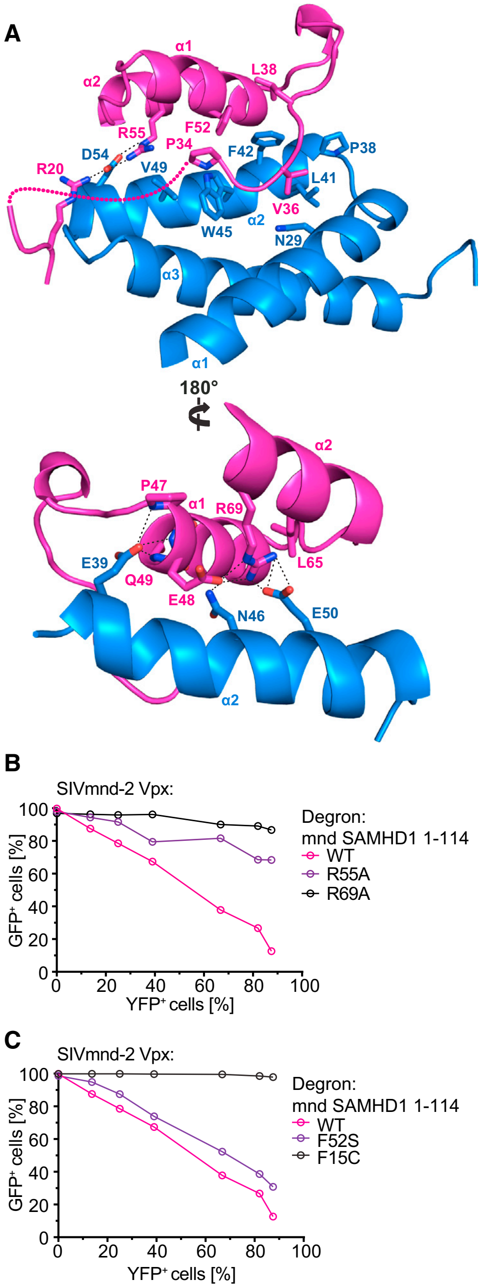

Figure 5. SAM Domain-SIV$_{mnd-2}$ Vpx Interactions

(A) The SAMHD1$_{mnd}$ SAM domain-SIV$_{mnd-2}$ Vpx interface. The SAMHD1$_{mnd}$ SAM domain (magenta) and SIV$_{mnd-2}$ Vpx (blue) are shown in cartoon representation. Residues making interactions are shown in stick representation with hydrogen bonding and salt bridges displayed as dashed lines.

(B and C) Quantification of reporter expression in cells stably expressing SAMHD1$_{mnd}$ (1–114) degron reporter constructs with (B) SAM domain point mutations and (C) mandrill to human SAMHD1 amino acid substitutions. Cells were transduced with increasing titers of particles expressing SIV$_{mnd-2}$ Vpx, and the level of transduction (YFP) and degron reporter (EGFP) expression was measured by flow cytometry.

domain-Vpx interaction (Figure 5A). In order to assess their role in the specificity of the Vpx-SAMHD1 interaction, F15 and F52 were mutated to cysteine and serine that are found in human SAMHD1 and their contribution tested in the degron assay (Figure 5C). These data show the F15C mutation renders SAMHD1$_{mnd}$ resistant to SIV$_{mnd-2}$ Vpx-induced proteasomal degradation but that surprisingly the F52S mutation only slightly reduces degradation of the degron reporter protein. Nevertheless, introduction of the reverse mutations C15F and S52F in human SAMHD1 has previously been shown to render human SAMHD1 sensitive to SIV$_{mnd-2}$ Vpx-mediated degradation (Wei et al., 2014). Therefore, taken together, these data support the idea that amino acid changes in primate SAM domain and NLS regions determine the lineage-specific formation of a Vpx-SAMHD1 interaction interface.

Specificity of Vpx-SAMHD1-NtD and -CtD Interactions

Despite similar overall structure and an identical mode of DCAF1 interaction (Figure S4), the SIV$_{mnd-2}$ Vpx/DCAF1 assembly recognizes the SAMHD1$_{mnd}$-NtD, while the HIV-2/SIV$_{mac}$/SIV$_{smm}$-type Vpx/DCAF1 are specific for the SAMHD1 C terminus. Comparison of the SIV$_{mnd-2}$ system presented here with the previously determined SIV$_{smm}$/DCAF1/SAMHD1-Ctd assembly (Schwefel et al., 2014) reveals the structural determinants for these fundamentally different specificities. In both complexes, a Vpx N-terminal arm and residues at the N terminus of α1 make contacts with either the amino-terminal region in SAMHD1$_{mnd}$ or the CtD region of SAMHD1$_{hs}$. The location of this contact on the DCAF1 disk is similar in both combined ternary interfaces (Figure 6A). However, the SAMHD1$_{mnd}$ N terminus makes additional contacts and occupies a larger surface area on Vpx and DCAF1, 750 Å^2 and 500 Å^2 versus 710 Å^2 and 210 Å^2 for the SAMHD1$_{hs}$ C terminus. In addition, in the mandrill complex, the Vpx arm folds over the first 20 residues of SAMHD1$_{mnd}$ trapping it against DCAF1, while the human SAMHD1 C-terminal peptide associates rather peripherally to the DCAF1/Vpx interface (Figure 6A).

In this context, it is important to note that Vpx amino acid residues at the N terminus of α1 that contribute to SAMHD1 binding differ significantly between SIV$_{smm}$ and SIV$_{mnd-2}$ type Vpx proteins constituting a variable region (VR1) (Figure S5). In the SIV$_{smm}$ complex, VR1 side chains of the conserved GEET-motif form salt bridges with basic residues in SAMHD1-CtD (Schwefel et al., 2014). However, in SIV$_{mnd-2}$ Vpx, VR1 comprises a proline (P9^{mnd-2}) followed by two glycine residues interspersed with a small hydrophobic amino acid and then a conserved acidic and hydrophobic residue (^{9}P[x]GAG[D/E]V/A^{15}). This configuration specifically accommodates the SAMHD1$_{mnd}$-NtD and sandwiches it between Vpx and the DCAF1 disk. The importance of this VR1 interaction for Vpx-mediated proteasomal degradation of SAMHD1$_{mnd}$ is demonstrated in EGFP-degron fusion protein assays where substitution of valine by bulky tryptophan at position 15 of SIV$_{mnd-2}$ Vpx substantially reduces degron reporter protein degradation (Figure 6B). In addition, analysis of the clustering of VR1 sequences (Figure S5) shows that SIV$_{rcm}$ and SIV$_{drl-1}$ Vpx that are SAMHD1-NtD specific also contain a SIV$_{mnd-2}$ type VR1, supporting the idea that VR1 is a major determinant for discrimination between SAMHD1 N-terminal and C-terminal target sequences.

As well as VR1, which confers NtD and CtD degron specificity to Vpx, a second region, VR2, in the α2-α3 loop and N terminus of α3 is conserved among Vpx but not in Vpr (Figure S5). Here,

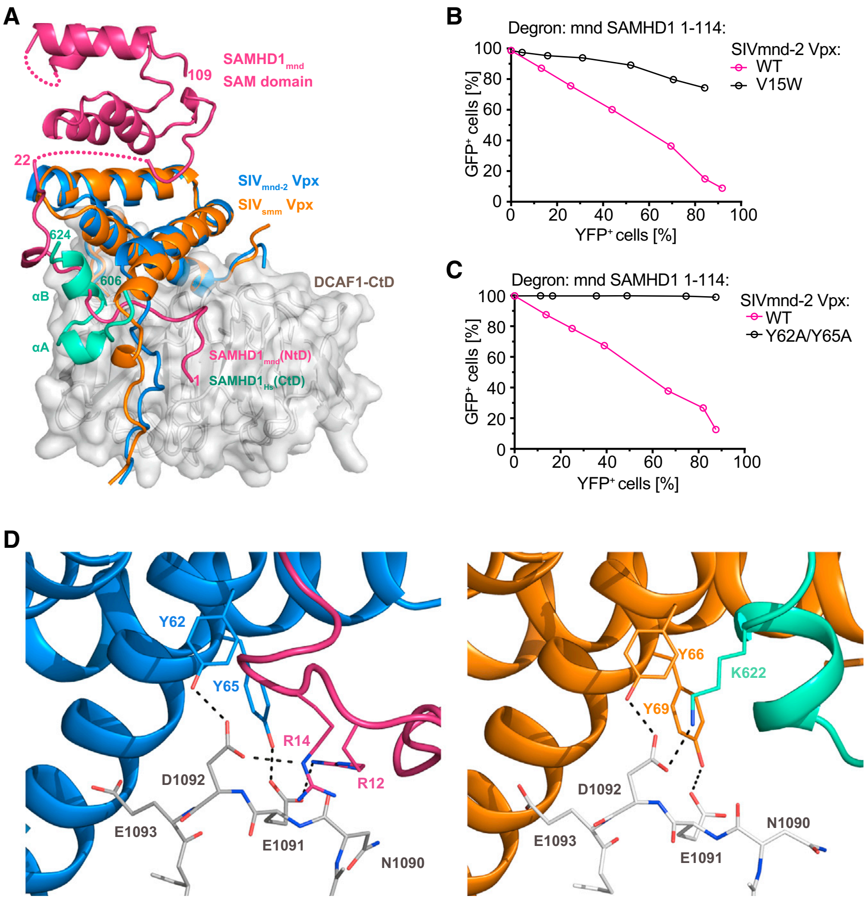

Figure 6. Comparison to the SIV_{smm} Vpx/DCAF1-CtD/SAMHD1$_{hs}$-CtD Structure

(A) Superposition of the SIV_{mnd-2} Vpx/DCAF1-CtD/SAMHD1$_{mnd}$-NtD and SIV_{smm} Vpx/DCAF1-CtD/SAMHD1$_{hs}$-CtD ternary complexes. Cartoons are colored as Figure 2 with the addition of SIV_{smm} Vpx (orange) and SAMHD1$_{hs}$-CtD (green). For clarity, only DCAF1-CtD from the mandrill complex is shown.

(B and C) Quantification of reporter expression in cells stably expressing the SAMHD1$_{mnd}$ (1–114) degron reporter construct after transduction with increasing titers of particles expressing SIV_{mnd-2} Vpx with mutations in (B) the N-terminal arm (V15W) and in (C) the di-tyrosine motif (Y62A/Y65A). The level of Vpx transduction (YFP) and degron reporter (EGFP) expression was measured by flow cytometry.

(D) Detailed view of the conserved VR2 Vpx di-tyrosine motif interaction with the DCAF1-CtD acidic loop and basic motifs of the SAMHD1$_{mnd}$-NtD (left) and SAMHD1$_{hs}$-CtD (right) degrons; see also Figure S5. Residues that contribute to the ternary interface are shown in stick representation with hydrogen bonding and salt bridge interactions displayed as dashed lines; see also Figure S6.

a di-tyrosine motif (Y62^{mnd-2}/Y66^{smm} and Y65^{mnd-2}/Y69^{smm}) is present, which engages the DCAF1 acidic loop (Figures 3B and S4B), providing a hydrophobic platform for binding of SAMHD1 residues (P13^{mnd} or V618^{hs}/F621^{hs}) and for positively charged amino acids (R12^{mnd}/R14^{mnd} or K622^{hs}) to form salt bridges to the DCAF1 acidic loop (Figure 6D). The importance of the Vpx di-tyrosine pair at the center of a ternary DCAF1-degron interaction was demonstrated in the degron assay where introduction of a double tyrosine to alanine mutant into SIV_{mnd-2} Vpx abolished reporter degradation (Figure 6C). Similarly, mutations of the basic residues that make salt bridges to the DCAF1 acidic loop also prevent degron reporter degradation (Schwefel et al., 2014) and in a pigtail macaque model SIV_{mne}, a Vpx Y66A, Y69A, Y71A triple mutant causes a reduction of viral load (Belshan et al., 2012). Taken together, these observations suggest that the VR2 region contains common binding determinants utilized in both SIV_{mnd-2} and SIV_{smm} type Vpx molecules for recognition of SAMHD1-NtD and SAMHD1-CtD degrons.

DISCUSSION

The current properties of accessory proteins Vpr and Vpx likely reflect a lengthy history of conflict between lentiviruses and their hosts. Eight different lineages of exogenous primate lentivirus including HIV-1 can be identified. All encode a Vpr that induces host G2/M cell-cycle arrest (Berger et al., 2015), probably by targeting a still poorly defined host cell protein for degradation through the proteasomal pathway or through interaction with proteins of the DNA damage pathway (Laguette et al., 2014). Vpr from two viral clades, typified by SIV_{agm} and SIV_{syk}, is also able to target SAMHD1 for degradation, facilitating infection of some types of non-dividing cells. In two further clades, both Vpr and Vpx are present with Vpr retaining only cell-cycle arrest activity and Vpx only inducing SAMHD1 degradation.

Phylogenetic reconstructions suggest that the Vprs from SIV_{agm} and SIV_{syk} acquired anti-SAMHD1 activity prior to the molecular events leading to the generation of Vpx (Lim et al., 2012). Moreover, it appears that once Vpr acquires anti-SAMHD1 activity, it does not lose it. This would imply that viruses containing both Vpr and Vpx arose by the recombination of two viruses; the first encoding an ancestral Vpr with the dual ability to cause cell-cycle arrest and degrade SAMHD1 and the second with a Vpr that had not acquired the capacity to induce SAMHD1 degradation. This allowed the ancestral, dual function Vpr to convert to Vpx and maintain SAMHD1 degradation activity but loose the cell-cycle arrest function provided by the second Vpr. Evaluation of our crystal structures is most informative in understanding these evolutionary changes and specifically the interaction between (i) Vpr/x and DCAF1 and (ii) between the two classes of Vpx and their SAMHD1 targets.

Amino acid residues that bind to DCAF1 and those that coordinate the bound zinc are highly conserved in all lentiviral Vpr/Vpx proteins (Figure S5), suggesting that the overall structure of the protein as well as mode of DCAF1 binding have been conserved during Vpr/Vpx evolution. This implies that the

HIV-1 Vpr/DCAF1 association is likely to be similar to the one observed in the SIV_{mnd-2} and SIV_{smm} (Schwefel et al., 2014) Vpx/DCAF1 crystal structures (Figure S4). Therefore, these structures might provide suitable starting points for rational design of compounds interfering with HIV-1 Vpr function even in the absence of definitive information concerning its cellular target. A notable exception is Vpr from SIV_{deb} that induces SAMHD1 degradation apparently without binding to DCAF1 (Berger et al., 2015), and it will be interesting to determine whether it utilizes an alternative pathway or different receptor protein to target SAMHD1.

Vpx from SIV_{smm} and SIV_{mnd-2} have been shown to interact with the C- and N-terminal regions of SAMHD1, respectively (Fregoso et al., 2013). Comparison of Vpx/SAMHD1/DCAF1 ternary complexes reveals that this discrimination of SAMHD1 N- and C-terminal degrons by the two classes of Vpx is determined both by polymorphisms in the SAMHD1 amino-terminal region (Figure S6) and by specific sequences in the VR1 region of Vpx (Figures 6 and S5). By contrast, in both classes of Vpx the VR2 region contains a conserved di-tyrosine pair that interacts with the DCAF1 acidic loop to provide a hydrophobic platform for N- and C-terminal SAMHD1 degrons. In this way, basic residues from both degron types are positioned for electrostatic interactions with the DCAF1 acidic loop. Interestingly, only the second tyrosine is present in SIV_{agm} Vpr proteins, which are incapable of inducing human SAMHD1 degradation, while the position corresponding to the first tyrosine is occupied by a glutamate residue (Figure S5). Gain of the bi-tyrosine motif might therefore have been a critical step in the development of Vpx allowing multiple modes of binding SAMHD1 while at the same time resulting in loss of the Vpr capacity to cause cell-cycle arrest.

A third highly variable region (VR3) in Vpr/Vpx proteins is directly C-terminal to the fourth conserved zinc-coordinating residue (Figure S5) that in both SIV_{smm} and SIV_{mnd-2} type Vpx contains proline-rich stretches of variable length. In the corresponding SAMHD1/Vpx/DCAF1 crystal structures, VR3 is either disordered (this study) or involved in crystal contacts (Schwefel et al., 2014), strongly suggesting that the region is not involved in SAMHD1 degron recognition. In agreement, SIV_{mac} Vpx lacking the poly-proline tail is still able to assemble into a ternary SAMHD1/Vpx/DCAF1 complex and to induce cullin-4-dependent SAMHD1 poly-ubiquitination (Ahn et al., 2012) and degradation (Belshan et al., 2012). Functional studies suggest involvement of VR3 in nuclear localization of Vpx and Vpr (Pancio et al., 2000; Zhou et al., 1998) with consequences for viral replication (Belshan et al., 2012), but notably, mutations in this region also influence protein expression levels significantly (Miyake et al., 2014).

Predictions regarding DCAF1/Vpr-mediated recruitment of target protein(s) that result in G2/M cell-cycle arrest remain speculative. In HIV-1, VR2 differs significantly from other Vpr and Vpx proteins, suggesting that in contrast to the Vpx di-tyrosine/DCAF1 acidic loop/SAMHD1 interaction, other principles may apply to HIV-1 Vpr target recognition. By contrast, VR1 of HIV-1 Vpr contains a ^{12}REP[F/Y/W][D/N]^{16} motif that is also conserved in SIV_{agm} Vpr proteins (Figure S5), suggesting similarities in VR1-mediated SIV_{agm} and HIV-1 Vpr target recruitment. Therefore, considering recent evidence for HIV-1 Vpr/DCAF1 association with components of the SLX4/MUS81/EME1 DNA-repair complex (Berger et al., 2015; Laguette et al., 2014), structural analysis of SIV_{agm} Vpr/DCAF1 together with the cognate $SAMHD1_{agm}$ will be of interest to further delineate the degron and/or target sequence(s) recognized by HIV-1 Vpr.

EXPERIMENTAL PROCEDURES

Degron Assay

Degron reporter constructs were generated by replacing the human SAMHD1-CtD degron sequence in pCMS28-NLS-EGFP-SAMHD1-CtD with N-terminal sequences from $SAMHD1_{mnd}$. Point mutations were created by site-directed mutagenesis. Virus-like particles (VLPs) were generated by co-transfecting 293T cells with pVSVG, pKB4 and pCMS28-NLS-EGFP-SAMHD1. Stable cell lines were produced by transduction of *Mus dunni* cells followed by puromycin selection. Expression of degron constructs was assessed using western blotting with anti-EGFP antibodies.

SIV_{smm} or SIV_{mnd-2} Vpx sequences were transferred into pLgatewayIeYFP (Gateway LR clonase™ II, Invitrogen) to create bicistronic Vpx-IRES-YFP expression constructs. Point mutations were created by site-directed mutagenesis. VLPs expressing Vpx-IRES-YFP were generated by co-transfecting 293T cells with pVSV-G, pKB4, and pLgatewayIeYFP-Vpx.

Parental *Mus dunni* or stable cell lines expressing degron reporters were seeded at 5×10^4 cells per well in a 24-well plate 1 day prior to infection with 2-fold serial dilutions of Vpx-YFP VLPs. After 48 hr, the percentage of EGFP- and YFP-positive cells was determined by flow cytometry.

Protein Expression and Purification

The nucleotide sequences coding for SIV_{mnd-2} Vpx isolate 5440 and amino acid residues 1–114 of $SAMHD1_{mnd}$ were inserted into pET-49b and pET-52b (Merck Millipore) expression plasmids respectively to generate N-terminally GST-tagged and Strep-II-tagged fusion proteins. SAMHD1 (1–114) was expressed in *E. coli* strain Rosetta 2 and purified using Strep-Tactin affinity followed by size-exclusion chromatography. SIV_{mnd-2} Vpx was captured from the bacterial lysate onto Glutathione Sepharose (GSH-Sepharose) (GE Healthcare) prior to an assembly reaction (see below). His-tagged DCAF1, residues 1,058–1,396 (DCAF1-CtD), was expressed in insect cells and purified using Ni-NTA Sepharose and size-exclusion chromatography.

For assembly, the GST-SIV_{mnd-2} Vpx bound beads were resuspended and incubated with 1 mg of DCAF1-CtD, an equimolar amount of $SAMHD1_{mnd}$-NtD, and 1 mg of HRV-3C protease (GE Healthcare) overnight at 4°C. After removal of the beads by centrifugation, the eluted ternary complex was further purified by size-exclusion chromatography. Details of protein expression constructs, protein production, and storage are provided in Supplemental Experimental Procedures.

Crystallization and Structure Solution

Crystals of the SIV_{mnd-2} Vpx/$SAMHD1_{mnd}$-NtD/DCAF1CtD complex were grown using the hanging drop vapor diffusion at 18°C in 2 μl droplets comprising 1 μl complex (6.34 mg/ml) and 1 μl of reservoir solution (0.16 M Trisodium Citrate-HCl [pH 5.2] and 4% PEG 6000). X-ray diffraction data were collected on beamline I04 at the Diamond Light Source, UK at a wavelength of 0.97965 Å. The structure was solved by molecular replacement using the previously determined DCAF1-CtD structure, and a homology model constructed with the previously determined SIV_{smm} Vpx as template (PDB: 4CC9). Details of crystallization, data collection, processing, and structure solution are provided in Supplemental Experimental Procedures.

Multiple Sequence Alignment

Amino acid sequences were aligned using the ClustalW server and adjusted manually. NCBI accession numbers for Vpr, Vpx, and SAMHD1 are provided in Supplemental Experimental Procedures.

ACCESSION NUMBERS

The coordinates and structure factors of the SIV_{mnd-2}-Vpx/$SAMHD_{mnd}$-NtD/DCAF1-CtD ternary complex have been deposited in the Protein Data Bank (PDB: 5aja).

SUPPLEMENTAL INFORMATION

Supplemental Information includes six figures, three table, and Supplemental Experimental Procedures and can be found with this article online at http://dx.doi.org/10.1016/j.chom.2015.03.004.

AUTHOR CONTRIBUTIONS

D.S., V.C.B., E.C., and P.A.W. performed experiments. D.S., V.C.B., E.C., P.A.W., J.P.S., K.N.B., and I.A.T. contributed to experimental design, data analysis, and manuscript writing. The authors declare no competing financial interests.

ACKNOWLEDGMENTS

We gratefully acknowledge Diamond Light Source for synchrotron access. This work was supported by the UK Medical Research Council file references U117512710 (J.P.S.), U117592729 (K.N.B.), and U117565647 (I.A.T.); The Wellcome Trust, ref 085955 (K.N.B.); an EMBO long-term fellowship co-funded by the European Commission Marie Curie Actions (EMBOCOFUND2010, GA-2010-267146) (D.S.); and an amfAR Mathilde Krim Fellowship in Basic Biomedical Research ref 108992-57-RKHF (D.S.).

Received: January 8, 2015
Revised: January 31, 2015
Accepted: February 27, 2015
Published: April 8, 2015

REFERENCES

Ahn, J., Hao, C., Yan, J., DeLucia, M., Mehrens, J., Wang, C., Gronenborn, A.M., and Skowronski, J. (2012). HIV/simian immunodeficiency virus (SIV) accessory virulence factor Vpx loads the host cell restriction factor SAMHD1 onto the E3 ubiquitin ligase complex CRL4DCAF1. J. Biol. Chem. *287*, 12550–12558.

Amie, S.M., Bambara, R.A., and Kim, B. (2013). GTP is the primary activator of the anti-HIV restriction factor SAMHD1. J. Biol. Chem. *288*, 25001–25006.

Baldauf, H.M., Pan, X., Erikson, E., Schmidt, S., Daddacha, W., Burggraf, M., Schenkova, K., Ambiel, I., Wabnitz, G., Gramberg, T., et al. (2012). SAMHD1 restricts HIV-1 infection in resting CD4(+) T cells. Nat. Med. *18*, 1682–1687.

Barry, M., and Früh, K. (2006). Viral modulators of cullin RING ubiquitin ligases: culling the host defense. Sci. STKE *2006*, pe21.

Beloglazova, N., Flick, R., Tchigvintsev, A., Brown, G., Popovic, A., Nocek, B., and Yakunin, A.F. (2013). Nuclease activity of the human SAMHD1 protein implicated in the Aicardi-Goutieres syndrome and HIV-1 restriction. J. Biol. Chem. *288*, 8101–8110.

Belshan, M., Kimata, J.T., Brown, C., Cheng, X., McCulley, A., Larsen, A., Thippeshappa, R., Hodara, V., Giavedoni, L., Hirsch, V., and Ratner, L. (2012). Vpx is critical for SIVmne infection of pigtail macaques. Retrovirology *9*, 32.

Bergamaschi, A., Ayinde, D., David, A., Le Rouzic, E., Morel, M., Collin, G., Descamps, D., Damond, F., Brun-Vezinet, F., Nisole, S., et al. (2009). The human immunodeficiency virus type 2 Vpx protein usurps the CUL4A-DDB1 DCAF1 ubiquitin ligase to overcome a postentry block in macrophage infection. J. Virol. *83*, 4854–4860.

Berger, A., Sommer, A.F., Zwarg, J., Hamdorf, M., Welzel, K., Esly, N., Panitz, S., Reuter, A., Ramos, I., Jatiani, A., et al. (2011). SAMHD1-deficient CD14+ cells from individuals with Aicardi-Goutières syndrome are highly susceptible to HIV-1 infection. PLoS Pathog. *7*, e1002425.

Berger, G., Lawrence, M., Hué, S., and Neil, S.J. (2015). G2/M cell cycle arrest correlates with primate lentiviral Vpr interaction with the SLX4 complex. J. Virol. *89*, 230–240.

Brandariz-Nuñez, A., Valle-Casuso, J.C., White, T.E., Laguette, N., Benkirane, M., Brojatsch, J., and Diaz-Griffero, F. (2012). Role of SAMHD1 nuclear localization in restriction of HIV-1 and SIVmac. Retrovirology *9*, 49.

Cribier, A., Descours, B., Valadão, A.L., Laguette, N., and Benkirane, M. (2013). Phosphorylation of SAMHD1 by cyclin A2/CDK1 regulates its restriction activity toward HIV-1. Cell Rep. *3*, 1036–1043.

Daugherty, M.D., and Malik, H.S. (2012). Rules of engagement: molecular insights from host-virus arms races. Annu. Rev. Genet. *46*, 677–700.

Descours, B., Cribier, A., Chable-Bessia, C., Ayinde, D., Rice, G., Crow, Y., Yatim, A., Schwartz, O., Laguette, N., and Benkirane, M. (2012). SAMHD1 restricts HIV-1 reverse transcription in quiescent CD4(+) T-cells. Retrovirology *9*, 87.

Fischer, E.S., Scrima, A., Böhm, K., Matsumoto, S., Lingaraju, G.M., Faty, M., Yasuda, T., Cavadini, S., Wakasugi, M., Hanaoka, F., et al. (2011). The molecular basis of CRL4DDB2/CSA ubiquitin ligase architecture, targeting, and activation. Cell *147*, 1024–1039.

Franzolin, E., Pontarin, G., Rampazzo, C., Miazzi, C., Ferraro, P., Palumbo, E., Reichard, P., and Bianchi, V. (2013). The deoxynucleotide triphosphohydrolase SAMHD1 is a major regulator of DNA precursor pools in mammalian cells. Proc. Natl. Acad. Sci. USA *110*, 14272–14277.

Fregoso, O.I., Ahn, J., Wang, C., Mehrens, J., Skowronski, J., and Emerman, M. (2013). Evolutionary toggling of Vpx/Vpr specificity results in divergent recognition of the restriction factor SAMHD1. PLoS Pathog. *9*, e1003496.

Goldstone, D.C., Ennis-Adeniran, V., Hedden, J.J., Groom, H.C., Rice, G.I., Christodoulou, E., Walker, P.A., Kelly, G., Haire, L.F., Yap, M.W., et al. (2011). HIV-1 restriction factor SAMHD1 is a deoxynucleoside triphosphate triphosphohydrolase. Nature *480*, 379–382.

Guo, Y., Dong, L., Qiu, X., Wang, Y., Zhang, B., Liu, H., Yu, Y., Zang, Y., Yang, M., and Huang, Z. (2014). Structural basis for hijacking CBF-β and CUL5 E3 ligase complex by HIV-1 Vif. Nature *505*, 229–233.

Hatziioannou, T., and Bieniasz, P.D. (2011). Antiretroviral restriction factors. Curr. Opin. Virol. *1*, 526–532.

Hatziioannou, T., Del Prete, G.Q., Keele, B.F., Estes, J.D., McNatt, M.W., Bitzegeio, J., Raymond, A., Rodriguez, A., Schmidt, F., Mac Trubey, C., et al. (2014). HIV-1-induced AIDS in monkeys. Science *344*, 1401–1405.

Hofmann, H., Logue, E.C., Bloch, N., Daddacha, W., Polsky, S.B., Schultz, M.L., Kim, B., and Landau, N.R. (2012). The Vpx lentiviral accessory protein targets SAMHD1 for degradation in the nucleus. J. Virol. *86*, 12552–12560.

Hrecka, K., Gierszewska, M., Srivastava, S., Kozaczkiewicz, L., Swanson, S.K., Florens, L., Washburn, M.P., and Skowronski, J. (2007). Lentiviral Vpr usurps Cul4-DDB1[VprBP] E3 ubiquitin ligase to modulate cell cycle. Proc. Natl. Acad. Sci. USA *104*, 11778–11783.

Hrecka, K., Hao, C., Gierszewska, M., Swanson, S.K., Kesik-Brodacka, M., Srivastava, S., Florens, L., Washburn, M.P., and Skowronski, J. (2011). Vpx relieves inhibition of HIV-1 infection of macrophages mediated by the SAMHD1 protein. Nature *474*, 658–661.

Ji, X., Wu, Y., Yan, J., Mehrens, J., Yang, H., DeLucia, M., Hao, C., Gronenborn, A.M., Skowronski, J., Ahn, J., and Xiong, Y. (2013). Mechanism of allosteric activation of SAMHD1 by dGTP. Nat. Struct. Mol. Biol. *20*, 1304–1309.

Karplus, P.A., and Diederichs, K. (2012). Linking crystallographic model and data quality. Science *336*, 1030–1033.

Kim, B., Nguyen, L.A., Daddacha, W., and Hollenbaugh, J.A. (2012). Tight interplay among SAMHD1 protein level, cellular dNTP levels, and HIV-1 proviral DNA synthesis kinetics in human primary monocyte-derived macrophages. J. Biol. Chem. *287*, 21570–21574.

Kretschmer, S., Wolf, C., König, N., Staroske, W., Guck, J., Häusler, M., Luksch, H., Nguyen, L.A., Kim, B., Alexopoulou, D., et al. (2015). SAMHD1 prevents autoimmunity by maintaining genome stability. Ann. Rheum. Dis. *74*, e17.

Laguette, N., Sobhian, B., Casartelli, N., Ringeard, M., Chable-Bessia, C., Ségéral, E., Yatim, A., Emiliani, S., Schwartz, O., and Benkirane, M. (2011). SAMHD1 is the dendritic- and myeloid-cell-specific HIV-1 restriction factor counteracted by Vpx. Nature *474*, 654–657.

Laguette, N., Rahm, N., Sobhian, B., Chable-Bessia, C., Münch, J., Snoeck, J., Sauter, D., Switzer, W.M., Heneine, W., Kirchhoff, F., et al. (2012). Evolutionary

and functional analyses of the interaction between the myeloid restriction factor SAMHD1 and the lentiviral Vpx protein. Cell Host Microbe *11*, 205–217.

Laguette, N., Brégnard, C., Hue, P., Basbous, J., Yatim, A., Larroque, M., Kirchhoff, F., Constantinou, A., Sobhian, B., and Benkirane, M. (2014). Premature activation of the SLX4 complex by Vpr promotes G2/M arrest and escape from innate immune sensing. Cell *156*, 134–145.

Lahouassa, H., Daddacha, W., Hofmann, H., Ayinde, D., Logue, E.C., Dragin, L., Bloch, N., Maudet, C., Bertrand, M., Gramberg, T., et al. (2012). SAMHD1 restricts the replication of human immunodeficiency virus type 1 by depleting the intracellular pool of deoxynucleoside triphosphates. Nat. Immunol. *13*, 223–228.

Le Rouzic, E., Belaïdouni, N., Estrabaud, E., Morel, M., Rain, J.C., Transy, C., and Margottin-Goguet, F. (2007). HIV1 Vpr arrests the cell cycle by recruiting DCAF1/VprBP, a receptor of the Cul4-DDB1 ubiquitin ligase. Cell Cycle *6*, 182–188.

Lim, E.S., Fregoso, O.I., McCoy, C.O., Matsen, F.A., Malik, H.S., and Emerman, M. (2012). The ability of primate lentiviruses to degrade the monocyte restriction factor SAMHD1 preceded the birth of the viral accessory protein Vpx. Cell Host Microbe *11*, 194–204.

Malim, M.H., and Bieniasz, P.D. (2012). HIV Restriction Factors and Mechanisms of Evasion. Cold Spring Harb. Perspect. Med. *2*, a006940.

Miazzi, C., Ferraro, P., Pontarin, G., Rampazzo, C., Reichard, P., and Bianchi, V. (2014). Allosteric regulation of the human and mouse deoxyribonucleotide triphosphohydrolase sterile α-motif/histidine-aspartate domain-containing protein 1 (SAMHD1). J. Biol. Chem. *289*, 18339–18346.

Mitchell, R.S., Katsura, C., Skasko, M.A., Fitzpatrick, K., Lau, D., Ruiz, A., Stephens, E.B., Margottin-Goguet, F., Benarous, R., and Guatelli, J.C. (2009). Vpu antagonizes BST-2-mediated restriction of HIV-1 release via beta-TrCP and endo-lysosomal trafficking. PLoS Pathog. *5*, e1000450.

Miyake, A., Fujita, M., Fujino, H., Koga, R., Kawamura, S., Otsuka, M., Ode, H., Iwatani, Y., Sakai, Y., Doi, N., et al. (2014). Poly-proline motif in HIV-2 Vpx is critical for its efficient translation. J. Gen. Virol. *95*, 179–189.

Neil, S.J., Zang, T., and Bieniasz, P.D. (2008). Tetherin inhibits retrovirus release and is antagonized by HIV-1 Vpu. Nature *451*, 425–430.

Pancio, H.A., Vander Heyden, N., and Ratner, L. (2000). The C-terminal proline-rich tail of human immunodeficiency virus type 2 Vpx is necessary for nuclear localization of the viral preintegration complex in nondividing cells. J. Virol. *74*, 6162–6167.

Pauls, E., Ruiz, A., Badia, R., Permanyer, M., Gubern, A., Riveira-Muñoz, E., Torres-Torronteras, J., Alvarez, M., Mothe, B., Brander, C., et al. (2014). Cell cycle control and HIV-1 susceptibility are linked by CDK6-dependent CDK2 phosphorylation of SAMHD1 in myeloid and lymphoid cells. J. Immunol. *193*, 1988–1997.

Powell, R.D., Holland, P.J., Hollis, T., and Perrino, F.W. (2011). Aicardi-Goutieres syndrome gene and HIV-1 restriction factor SAMHD1 is a dGTP-regulated deoxynucleotide triphosphohydrolase. J. Biol. Chem. *286*, 43596–43600.

Rehwinkel, J., Maelfait, J., Bridgeman, A., Rigby, R., Hayward, B., Liberatore, R.A., Bieniasz, P.D., Towers, G.J., Moita, L.F., Crow, Y.J., et al. (2013). SAMHD1-dependent retroviral control and escape in mice. EMBO J. *32*, 2454–2462.

Rice, G.I., Bond, J., Asipu, A., Brunette, R.L., Manfield, I.W., Carr, I.M., Fuller, J.C., Jackson, R.M., Lamb, T., Briggs, T.A., et al. (2009). Mutations involved in Aicardi-Goutières syndrome implicate SAMHD1 as regulator of the innate immune response. Nat. Genet. *41*, 829–832.

Ryoo, J., Choi, J., Oh, C., Kim, S., Seo, M., Kim, S.Y., Seo, D., Kim, J., White, T.E., Brandariz-Nuñez, A., et al. (2014). The ribonuclease activity of SAMHD1 is required for HIV-1 restriction. Nat. Med. *20*, 936–941.

Schwefel, D., Groom, H.C., Boucherit, V.C., Christodoulou, E., Walker, P.A., Stoye, J.P., Bishop, K.N., and Taylor, I.A. (2014). Structural basis of lentiviral subversion of a cellular protein degradation pathway. Nature *505*, 234–238.

Sharp, P.M., and Hahn, B.H. (2011). Origins of HIV and the AIDS pandemic. Cold Spring Harb. Perspect. Med. *1*, a006841.

Sheehy, A.M., Gaddis, N.C., Choi, J.D., and Malim, M.H. (2002). Isolation of a human gene that inhibits HIV-1 infection and is suppressed by the viral Vif protein. Nature *418*, 646–650.

Spragg, C.J., and Emerman, M. (2013). Antagonism of SAMHD1 is actively maintained in natural infections of simian immunodeficiency virus. Proc. Natl. Acad. Sci. USA *110*, 21136–21141.

Srivastava, S., Swanson, S.K., Manel, N., Florens, L., Washburn, M.P., and Skowronski, J. (2008). Lentiviral Vpx accessory factor targets VprBP/DCAF1 substrate adaptor for cullin 4 E3 ubiquitin ligase to enable macrophage infection. PLoS Pathog. *4*, e1000059.

St Gelais, C., de Silva, S., Amie, S.M., Coleman, C.M., Hoy, H., Hollenbaugh, J.A., Kim, B., and Wu, L. (2012). SAMHD1 restricts HIV-1 infection in dendritic cells (DCs) by dNTP depletion, but its expression in DCs and primary CD4+ T-lymphocytes cannot be upregulated by interferons. Retrovirology *9*, 105.

Strebel, K. (2013). HIV accessory proteins versus host restriction factors. Curr. Opin. Virol. *3*, 692–699.

Stremlau, M., Owens, C.M., Perron, M.J., Kiessling, M., Autissier, P., and Sodroski, J. (2004). The cytoplasmic body component TRIM5alpha restricts HIV-1 infection in Old World monkeys. Nature *427*, 848–853.

Towers, G.J., and Noursadeghi, M. (2014). Interactions between HIV-1 and the cell-autonomous innate immune system. Cell Host Microbe *16*, 10–18.

Tüngler, V., Staroske, W., Kind, B., Dobrick, M., Kretschmer, S., Schmidt, F., Krug, C., Lorenz, M., Chara, O., Schwille, P., and Lee-Kirsch, M.A. (2013). Single-stranded nucleic acids promote SAMHD1 complex formation. J. Mol. Med. *91*, 759–770.

Wei, W., Guo, H., Gao, Q., Markham, R., and Yu, X.F. (2014). Variation of two primate lineage-specific residues in human SAMHD1 confers resistance to N terminus-targeted SIV Vpx proteins. J. Virol. *88*, 583–591.

White, T.E., Brandariz-Nuñez, A., Valle-Casuso, J.C., Amie, S., Nguyen, L.A., Kim, B., Tuzova, M., and Diaz-Griffero, F. (2013). The retroviral restriction ability of SAMHD1, but not its deoxynucleotide triphosphohydrolase activity, is regulated by phosphorylation. Cell Host Microbe *13*, 441–451.

Wolf, D., and Goff, S.P. (2008). Host restriction factors blocking retroviral replication. Annu. Rev. Genet. *42*, 143–163.

Zhou, Y., Lu, Y., and Ratner, L. (1998). Arginine residues in the C-terminus of HIV-1 Vpr are important for nuclear localization and cell cycle arrest. Virology *242*, 414–424.

Zhu, C., Gao, W., Zhao, K., Qin, X., Zhang, Y., Peng, X., Zhang, L., Dong, Y., Zhang, W., Li, P., et al. (2013). Structural insight into dGTP-dependent activation of tetrameric SAMHD1 deoxynucleoside triphosphate triphosphohydrolase. Nat. Commun. *4*, 2722.

Zimmerman, E.S., Schulman, B.A., and Zheng, N. (2010). Structural assembly of cullin-RING ubiquitin ligase complexes. Curr. Opin. Struct. Biol. *20*, 714–721.

Cell Host & Microbe, Volume *17*

Supplemental Information

Molecular Determinants for Recognition

of Divergent SAMHD1 Proteins

by the Lentiviral Accessory Protein Vpx

David Schwefel, Virginie C. Boucherit, Evangelos Christodoulou, Philip A. Walker, Jonathan P. Stoye, Kate N. Bishop, and Ian A. Taylor

Molecular determinants for recognition of divergent SAMHD1 proteins by the lentiviral accessory protein Vpx.

INVENTORY OF SUPPLEMENTAL INFORMATION

ITEM 1) **Figure S1**. Western blot analysis of expression and primary FACS data for SAMHD1 degron assay. Panel **A** of this item shows the level of expression of each SAMHD1 degron used in the study. Panel **B** shows the primary FACS data from individual degron assays. Both **A** and **B** are associated with Figures 1, 4, 5 and 6 in the main article.

ITEM 2) **Figure S2**. Experimental electron density for each component of the SIV_{mnd-2} Vpx/$SAMHD_{mnd}$-NtD/DCAF1-CtD ternary complex. This item shows the quality of the maps used to build the structure presented in the paper and is associated with Table 1 and Figures 2-6 in the main article.

ITEM 3) **Figure S3**. Zinc ion co-ordination of Vpx SIV_{mnd-2} and Vpx SIV_{smm}. This item shows the details of the co-ordinated zinc ions bound in the Vpx SIV_{mnd-2} and Vpx SIV_{smm} structures. It is associated with Figure 2 in the main article.

ITEM 4) **Figure S4**. Superposition of Vpx SIV_{mnd-2} and Vpx SIV_{smm} ternary complexes. This item shows the structural overlap between Vpx SIV_{mnd-2} and Vpx SIV_{smm} at the DCAF1 interface. This alignment facilitates a comparison of the common interactions and is associated with Figure 3 of the main article.

ITEM 5) **Figure S5**. Multiple sequence alignment of Vpx and Vpr proteins. This alignment in combination with Vpx SIV_{mnd-2} and Vpx SIV_{smm} structures shows all the conserved and variable regions found in the different Vpx and Vpr lineages and is associated with Figure 3 and 6 of the main article.

ITEM 6) **Figure S6**. SAMHD1 multiple sequence alignment. This alignment shows the sequence conservation in SAMHD1 NtD and CtD degron regions and highlights the residues making contacts at the Vpx interface. It is associated with Figure 5 and 6 of the main article.

ITEM 7) Supplemental Figure legends. Detailed legends for Supplemental Figures S1-S6.

ITEM 8) **Supplemental Table S1A-C**. These tables provide a list of all the interactions observed in the $SIV_{mnd\text{-}2}$ Vpx/$SAMHD_{mnd}$-NtD/DCAF1-CtD ternary complex and are associated Figures 3-6 of the main article.

ITEM 9) SUPPLEMENTAL EXPERIMENTAL PROCEDURES

This item provides detailed methods to supplement the experimental procedures reported in the paper.

ITEM 10) SUPPLEMENTAL REFERENCES

This item contains the full references that are cited Supplemental Experimental Procedures

Figure S1

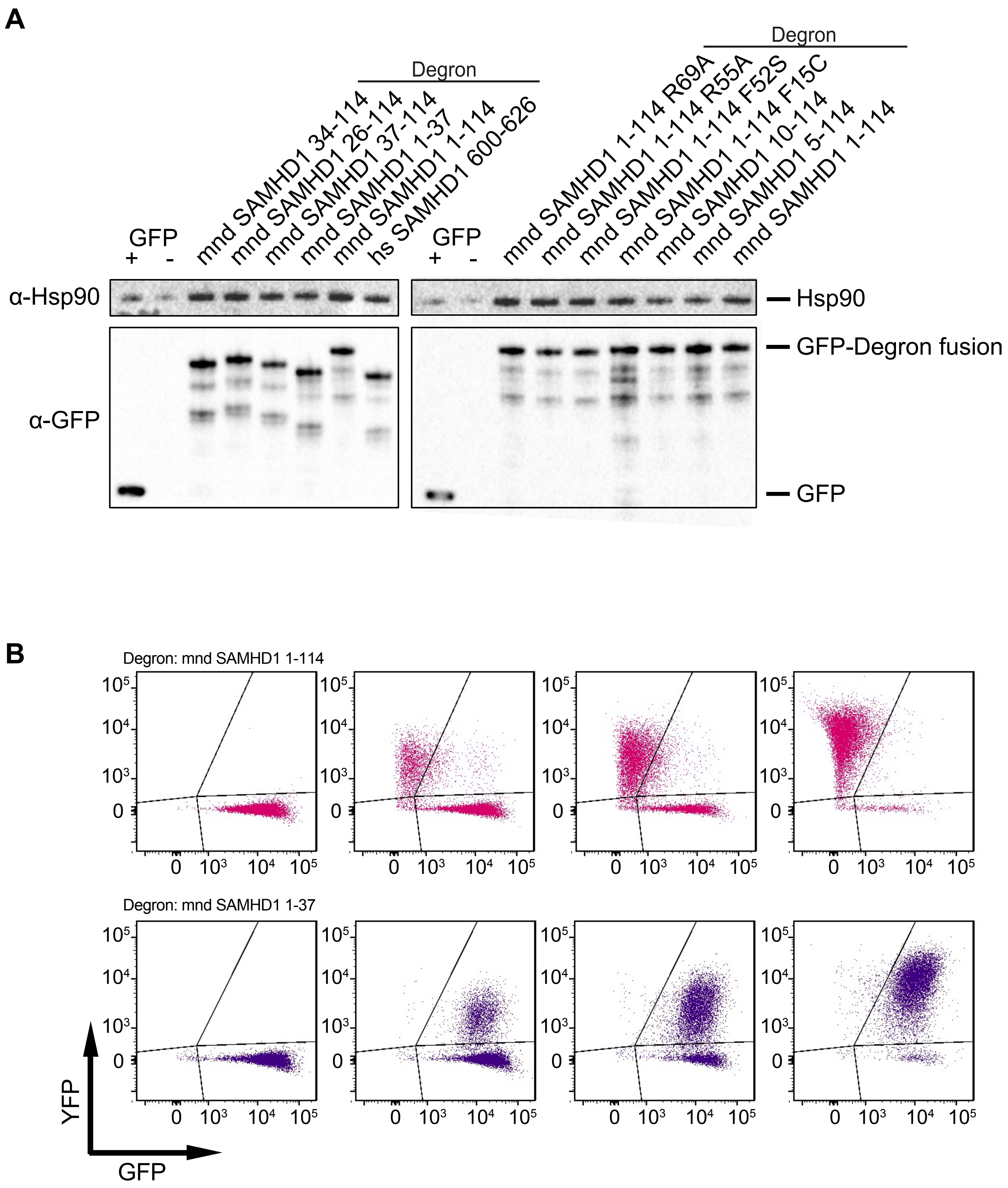
A
Degron
Degron
GFP
+ -
mnd SAMHD1 34-114
mnd SAMHD1 26-114
mnd SAMHD1 37-114
mnd SAMHD1 1-37
mnd SAMHD1 1-114
hs SAMHD1 600-626
mnd SAMHD1 1-114 R69A
mnd SAMHD1 1-114 R55A
mnd SAMHD1 1-114 F52S
mnd SAMHD1 1-114 F15C
mnd SAMHD1 10-114
mnd SAMHD1 5-114
mnd SAMHD1 1-114
α-Hsp90
α-GFP
Hsp90
GFP-Degron fusion
GFP
B
Degron: mnd SAMHD1 1-114
Degron: mnd SAMHD1 1-37
YFP
GFP

Figure S2

DCAF1-CtD

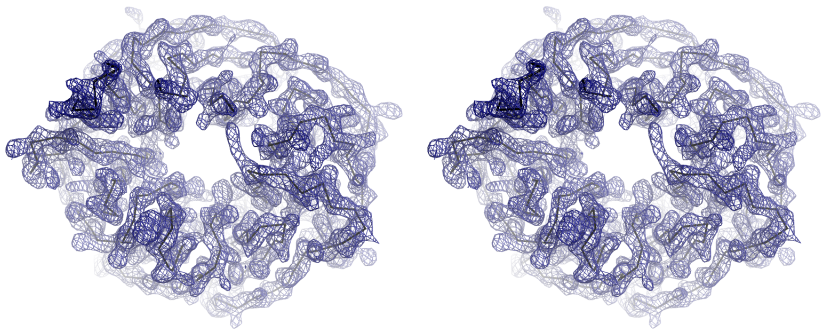

SIV$_{mnd-2}$ Vpx

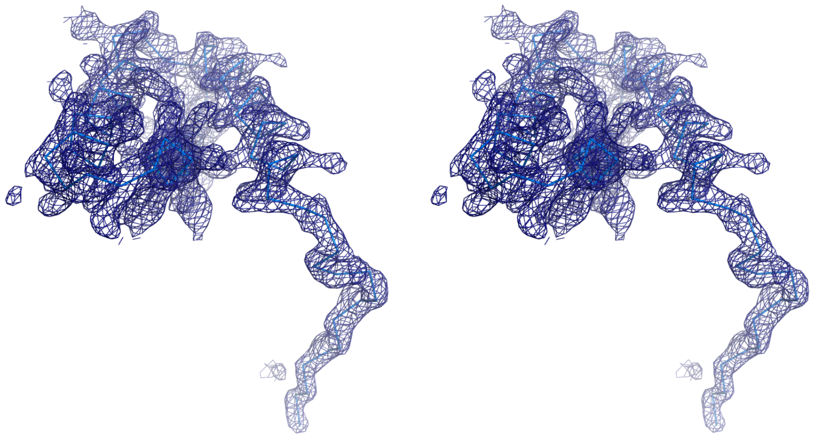

SAMHD1$_{mnd}$ 1-114

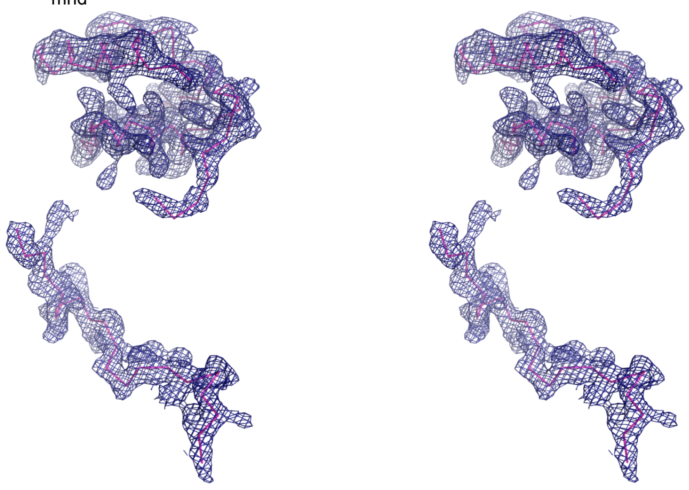

Figure S3

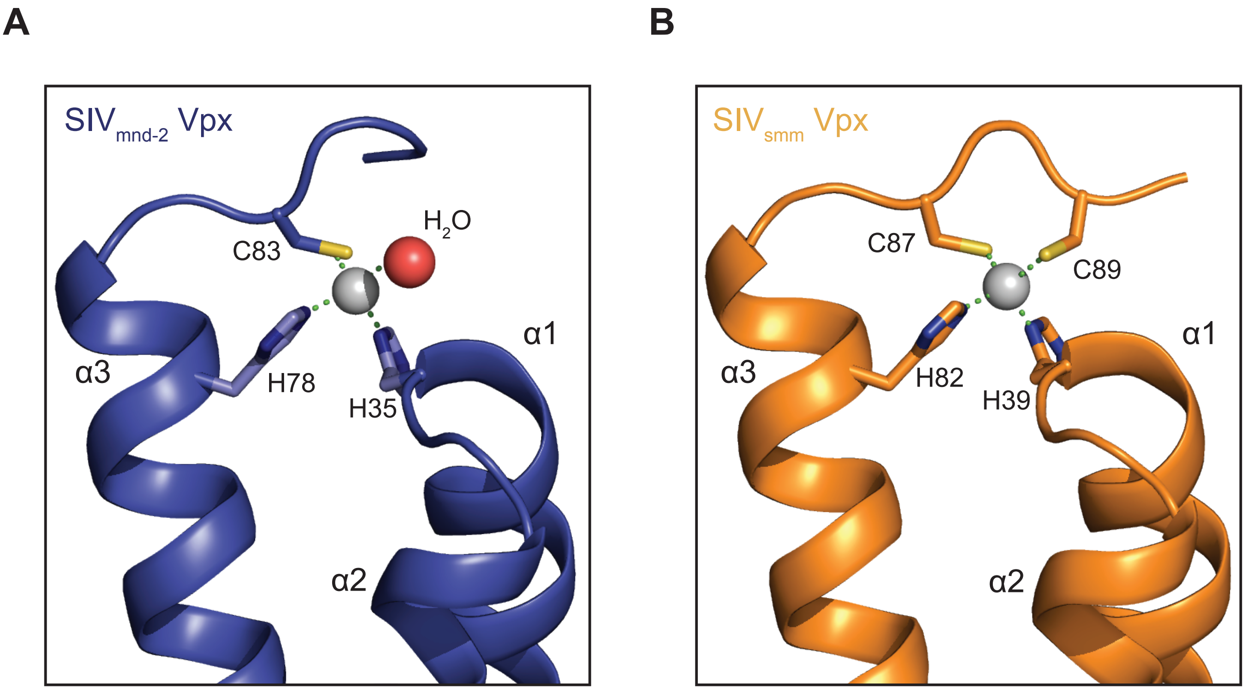
A
SIV mnd-2 Vpx
H2O
C83
α3
H78
H35
α1
α2
B
SIV smm Vpx
C87
C89
α3
H82
H39
α1
α2

Figure S4

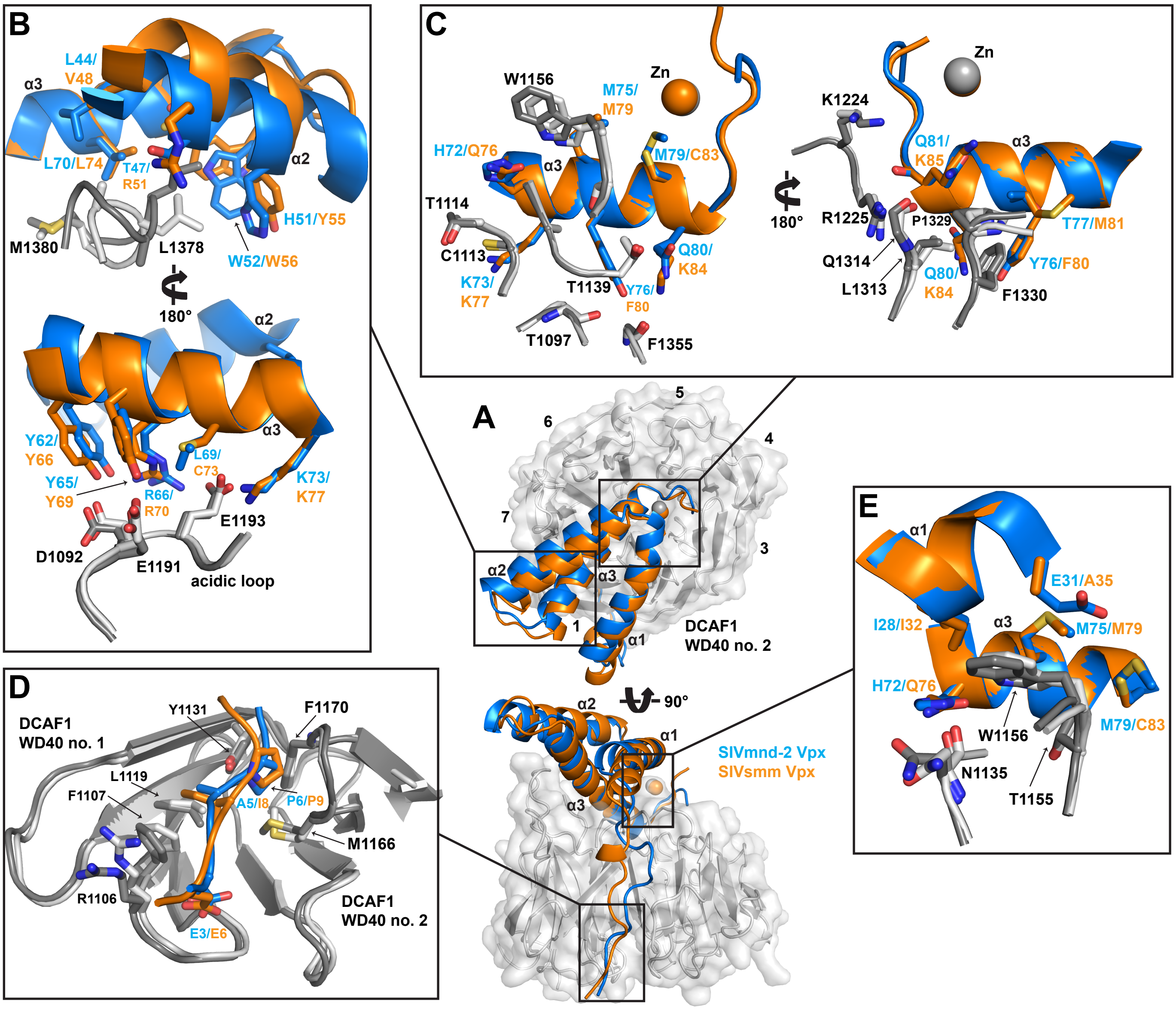

B
L44/
V48
α3
L70/L74
T47/
R51
α2
H51/Y55
M1380
L1378
W52/W56
180°
α2
α3
Y62/
Y66
Y65/
Y69
R66/
R70
L69/
C73
K73/
K77
E1193
D1092
E1191
acidic loop
C
W1156
Zn
M75/
M79
H72/Q76
α3
M79/C83
T1114
C1113
K73/
K77
T1139
Y76/
F80
Q80/
K84
T1097
F1355
Zn
K1224
Q81/
K85
α3
180°
R1225
P1329
T77/M81
Q1314
L1313
Q80/
K84
Y76/F80
F1330
A
6
5
4
7
3
α2
α3
1
α1
DCAF1
WD40 no. 2
α2
90°
α1
SIVmnd-2 Vpx
SIVsmm Vpx
α3
D
DCAF1
WD40 no. 1
Y1131
F1170
L1119
F1107
A5/I8
P6/P9
M1166
R1106
E3/E6
DCAF1
WD40 no. 2
E
α1
E31/A35
I28/I32
α3
M75/M79
H72/Q76
W1156
M79/C83
N1135
T1155

Figure S5

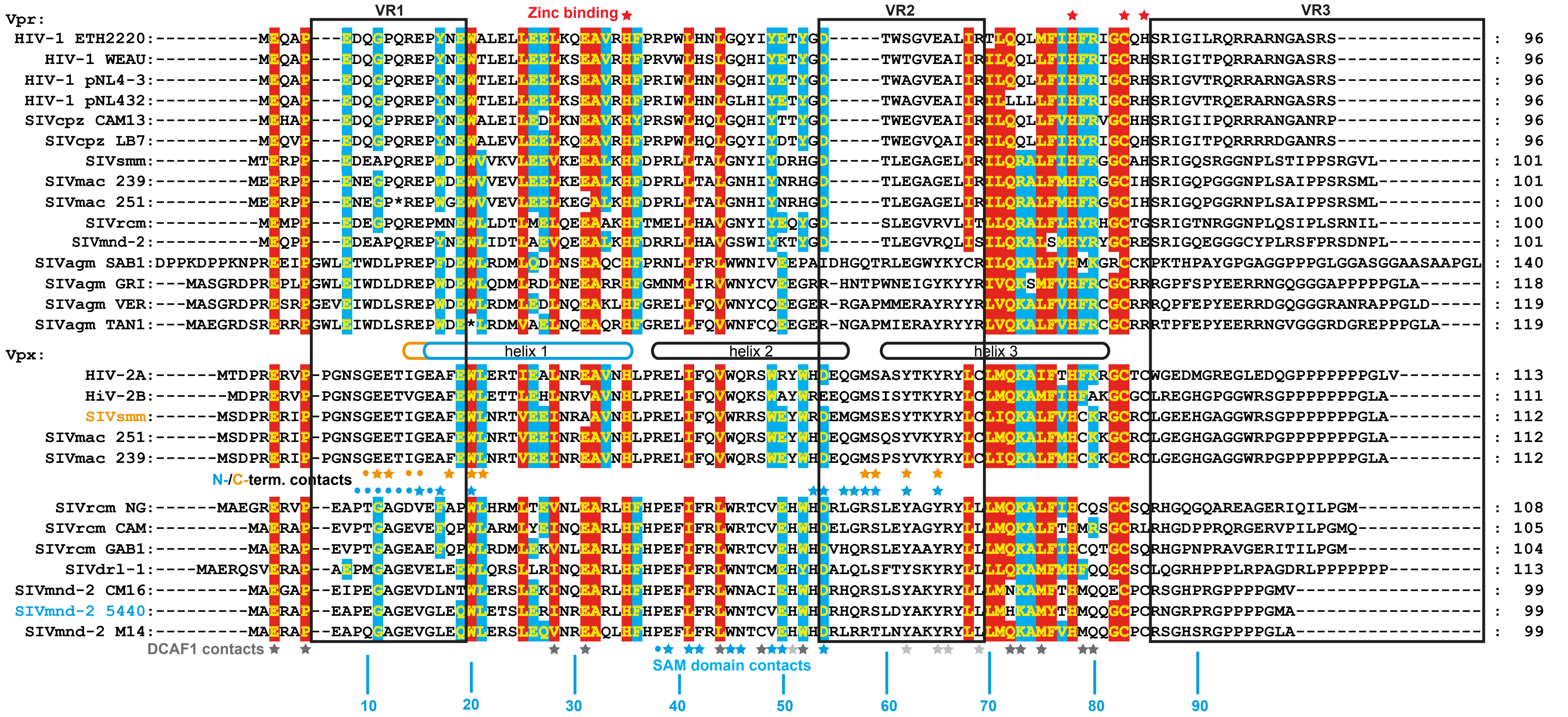

VR1
Zinc binding
VR2
VR3
Vpr:
HIV-1 ETH2220:----------MEQAP---EDQGPQREPYNEWALELLEELKQEAVRHFPRPWLHNLGQYIYETYGD-----TWSGVEALIRTLQQLMFIHFRIGCQHSRIGILRQRRARNGASRS-------------- : 96
HIV-1 WEAU:----------MEQAP---EDQGPQREPYNEWTLELLEELKSEAVRHFPRVWLHSLGQHIYETYGD-----TWTGVEAIIRILQQLLFIHFRIGCRHSRIGITPQRRARNGASRS-------------- : 96
HIV-1 pNL4-3:----------MEQAP---EDQGPQREPYNEWTLELLEELKSEAVRHFPRIWLHNLGQHIYETYGD-----TWAGVEAIIRILQQLLFIHFRIGCRHSRIGVTRQRRARNGASRS-------------- : 96
HIV-1 pNL432:----------MEQAP---EDQGPQREPYNEWTLELLEELKSEAVRHFPRIWLHNLGLHIYETYGD-----TWAGVEAIIRILLLLLFIHFRIGCRHSRIGVTRQERARNGASRS-------------- : 96
SIVcpz CAM13:----------MEHAP---EDQGPPREPYNEWALEILEDLKNEAVKHYPRSWLHQLGQHIYTTYGD-----TWEGVEAIIRILQQLLFVHFRVGCHHSRIGIIPQRRRANGANRP-------------- : 96
SIVcpz LB7:----------MEQVP---EDQGPQREPYNEWALEVLEELKQEAVRHFPRPWLHQLGQYIYDTYGD-----TWEGVQAIIRILQQLLFIHYRIGCQHSRIGITPQRRRRDGANRS-------------- : 96
SIVsmm:---------MTERPP---EDEAPQREPWDEWVVKVLEEVKEEALKHFDPRLLTALGNYIYDRHGD-----TLEGAGELIRILQRALFIHFRGGCAHSRIGQSRGGNPLSTIPPSRGVL---------- : 101
SIVmac 239:---------MEERPP---ENEGPQREPWDEWVVEVLEELKEEALKHFDPRLLTALGNHIYNRHGD-----TLEGAGELIRILQRALFMHFRGGCIHSRIGQPGGGNPLSAIPPSRSML---------- : 101
SIVmac 251:---------MEERPP---ENEGP*REPWGEWVVEVLEELKEGALKHFDPRLLTALGNHIYNRHGD-----TLEGAGELIRILQRALFMHFRGGCIHSRIGQPGGRNPLSAIPPSRSML---------- : 100
SIVrcm:----------MEMPP---EDEGPQREPMNEWLLDTLMELQEEAAKHFTMELLHAVGNYIYEQYGD-----SLEGVRVLITLLQRALFLHYRHGCTGSRIGTNRGGNPLQSIPLSRNIL---------- : 100
SIVmnd-2:----------MEQPP---EDEAPQREPYNEWLIDTLAEVQEEALKHFDRRLLHAVGSWIYKTYGD-----TLEGVRQLISILQKALSMHYRYGCRESRIGQEGGGCYPLRSFPRSDNPL--------- : 101
SIVagm SAB1:DPPKDPPKNPREEIPGWLETWDLPREPFDEWLRDMLQDLNSEAQCHFPRNLLFRLWWNIVEEPAIDHGQTRLEGWYKYCRILQKALFVHMKGRCCKPKTHPAYGPGAGGPPPGLGGASGGAASAAPGL : 140
SIVagm GRI:---MASGRDPREPLPGWLEIWDLDREPWDEWLQDMLRDLNEEARRHFGMNMLIRVWNYCVEEGRR-HNTPWNEIGYKYYRIVQKSMFVHFRCGCRRRGPFSPYEERRNGQGGGAPPPPPGLA------ : 118
SIVagm VER:---MASGRDPRESRPGEVEIWDLSREPWDEWLRDMLEDLNQEAKLHFGRELLFQVWNYCQEEGER-RGAPMMERAYRYYRLVQKALFVHFRCGCRRRQPFEPYEERRDGQGGGRANRAPPGLD----- : 119
SIVagm TAN1:---MAEGRDSRERRPGWLEIWDLSREPWDE*LRDMVAELNQEAQRHFGRELLFQVWNFCQEEGER-NGAPMIERAYRYYRLVQKALFVHFRCGCRRRTPFEPYEERRNGVGGGRDGREPPPGLA---- : 119
helix 1
helix 2
helix 3
Vpx:
HIV-2A:------MTDPRERVP-PGNSGEETIGEAFEWLERTIEALNREAVNHLPRELIFQVWQRSWRYWHDEQGMSASYTKYRYLCLMQKAIFTHFKRGCTCWGEDMGREGLEDQGPPPPPPPPGLV-------- : 113
HiV-2B:-------MDPRERVP-PGNSGEETVGEAFEWLETTLEHLNRVAVNHLPRELIFQVWQKSWAYWREEQGMSISYTKYRYLCLMQKAMFIHFAKGCGCLREGHGPGGWRSGPPPPPPPGLA--------- : 111
SIVsmm:------MSDPRERIP-PGNSGEETIGEAFEWLNRTVEEINRAAVNHLPRELIFQVWRRSWEYWRDEMGMSESYTKYRYLCLIQKALFVHCKRGCRCLGEEHGAGGWRSGPPPPPPPGLA--------- : 112
SIVmac 251:------MSDPRERIP-PGNSGEETIGEAFEWLNRTVEEINREAVNHLPRELIFQVWQRSWEYWHDEQGMSQSYVKYRYLCLMQKALFMHCKKGCRCLGEGHGAGGWRPGPPPPPPPGLA--------- : 112
SIVmac 239:------MSDPRERIP-PGNSGEETIGEAFEWLNRTVEEINREAVNHLPRELIFQVWQRSWEYWHDEQGMSPSYVKYRYLCLIQKALFMHCKKGCRCLGEGHGAGGWRPGPPPPPPPGLA--------- : 112
N-/C-term. contacts
SIVrcm NG:------MAEGRERVP--EAPTGAGDVEFAPWLHRMLTEVNLEARLHFHPEFIFRLWRTCVEHWHDRLGRSLEYAGYRYLLLMQKALFIHCQSGCSQRHGQGQAREAGERIQILPGM----------- : 108
SIVrcm CAM:---------MAERAP--EVPTGAGEVEFQPWLARMLYEINQEARLHFHPEFIFRLWRTCVEHWHDELGRSLEYAGYRYLLLMQKALFTHMRSGCRLRHGDPPRQRGERVPILPGMQ----------- : 105
SIVrcm GAB1:---------MAERAP--EVPTGAGEAEFQPWLRDMLEKVNLEARLHFHPEFIFRLWRTCVEHWHDVHQRSLEYAAYRYLLLMQKALFIHCQTGCSQRHGPNPRAVGERITILPGM------------ : 104
SIVdrl-1:----MAERQSVERAP--AEPMGAGEVELEEWLQRSLLRINQEARLHFHPEFLFRLWNTCMEHYHDALQLSFTYSKYRYLLLLQKAMFMHFQQGCSCLQGRHPPPLRPAGDRLPPPPPPP--------- : 113
SIVmnd-2 CM16:---------MAEGAP--EIPEGAGEVDLNTWLERSLEKINQEARLHFHPEFLFRLWNACIEHWHDRHQRSLSYAKYRYLLLMNKAMFTHMQQECPCRSGHPRGPPPPGMV------------------ : 99
SIVmnd-2 5440:---------MAERAP--EAPEGAGEVGLEQWLETSLERINREARLHFHPEFLFRLWNTCVEHWHDRHQRSLDYAKYRYLLLMHKAMYTHMQQGCPCRNGRPRGPPPPGMA------------------ : 99
SIVmnd-2 M14:---------MAERAP--EAPQGAGEVGLEQWLERSLEQVNREAQLHFHPEFLFRLWNTCVEHWHDRLRRTLNYAKYRYLLLMQKAMFVHMQQGCPCRSGHSRGPPPPGLA------------------ : 99
DCAF1 contacts
SAM domain contacts
10
20
30
40
50
60
70
80
90

Figure S6

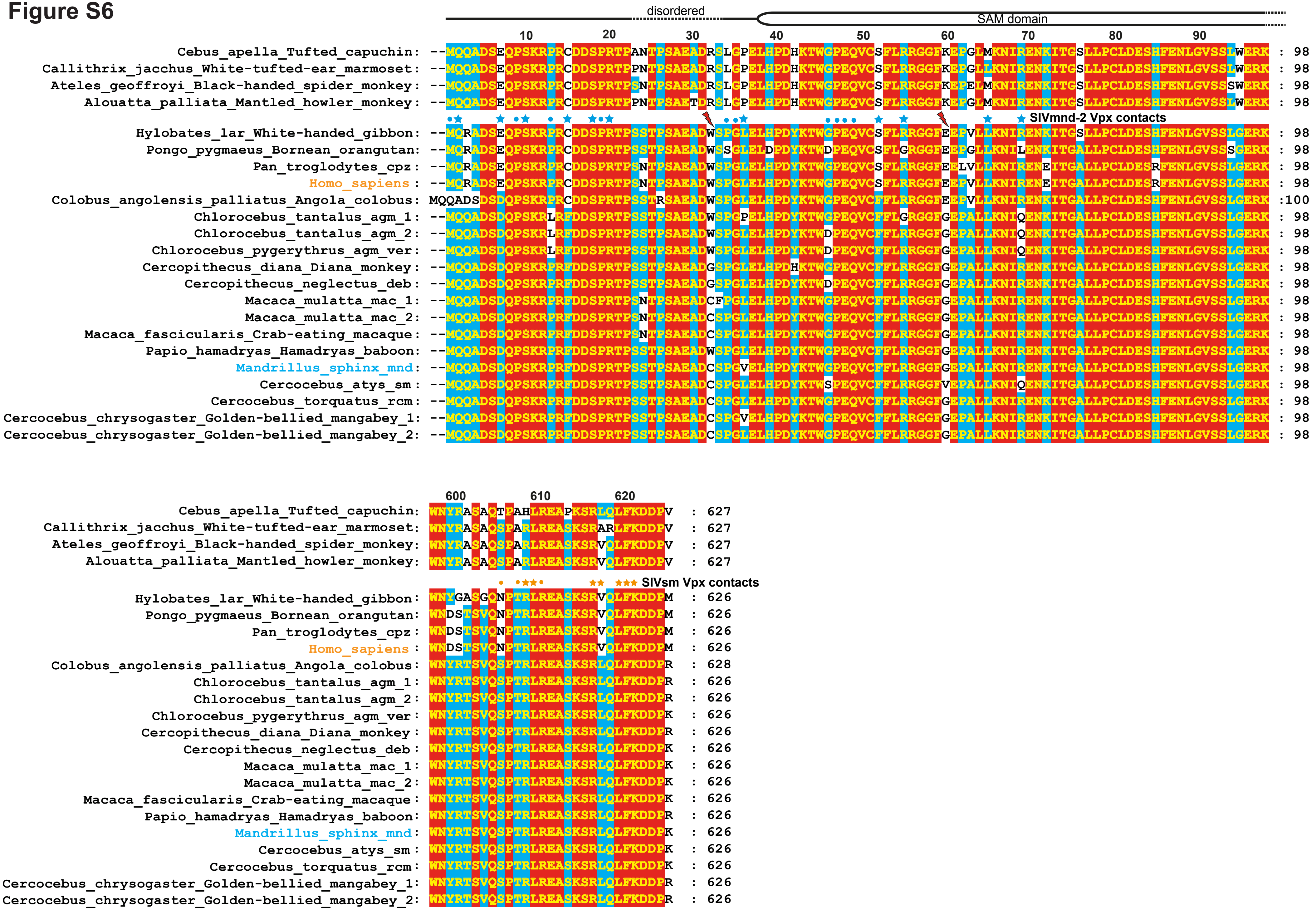
disordered
SAM domain
10 20 30 40 50 60 70 80 90
Cebus_apella_Tufted_capuchin: --MQQADSEQPSKRPRCDDSPRTPANTPSAEADRSLGPELHPDHKTWGPEQVCSFLRRGGEKEEGLMKNIRENKITGSLLPCLDESHFENLGVSSLWERK : 98
Callithrix_jacchus_White-tufted-ear_marmoset: --MQQADSEQPSKRPRCDDSPRTPPNTPSAEADRSLGPELHPDHKTWGPEQVCSFLRRGGEKEEGLLKNIRENKITGSLLPCLDESHFENLGVSSLWERK : 98
Ateles_geoffroyi_Black-handed_spider_monkey: --MQQADSEQPSKRPRCDDSPRTPSNTPSAEADRSLGPELHPDHKTWGPEQVCSFLRRGGEKEELMKNIRENKITGSLLPCLDESHFENLGVSSSWERK : 98
Alouatta_palliata_Mantled_howler_monkey: --MQQADSEQPSKRPRCDDSPRTPPNTPSAETDRSLGPELHPDHKTWGPEQVCSFLRRGGEKEEGLMKNIRENKITGSLLPCLDESHFENLGVSSLWERK : 98
SIVmnd-2 Vpx contacts
Hylobates_lar_White-handed_gibbon: --MQRADSEQPSKRPRCDDSPRTPSSTPSAEADWSPGLELHPDYKTWGPEQVCSFLRRGGEEEPVLLKNIRENKITGSLLPCLDESHFENLGVSSLGERK : 98
Pongo_pygmaeus_Bornean_orangutan: --MQRADSEQPSKRPRCDDSPRTPSSTPSAEADWSSGLELDPDYKTWDPEQVCSFLGRGGEEEPGLLKNILENKITGALLPCLDESHFENLGVSSSGERK : 98
Pan_troglodytes_cpz: --MQRADSEQPSKRPRCDDSPRTPSNTPSAEADWSPGLELHPDYKTWGPEQVCSFLRRGGEEELVLLKNIRENEITGALLPCLDESRFENLGVSSLGERK : 98
Homo_sapiens: --MQRADSEQPSKRPRCDDSPRTPSNTPSAEADWSPGLELHPDYKTWGPEQVCSFLRRGGEEEPVLLKNIRENEITGALLPCLDESRFENLGVSSLGERK : 98
Colobus_angolensis_palliatus_Angola_colobus: MQQADSDSDQPSKRPRCDDSPRTPSSTRSAEADWSPGLELHPDYKTWGPEQVCFFLRRGGEEEPVLLKNIRENKITGALLPCLDESHFENLGVSSLGERK :100
Chlorocebus_tantalus_agm_1: --MQQADSDQPSKRLRFDDSPRTPSSTPSAEADWSPGPELHPDYKTWGPEQVCFFLGRGGGEPALLKNIQENKITGALLPCLDESHFENLGVSSLGERK : 98
Chlorocebus_tantalus_agm_2: --MQQADSDQPSKRLRFDDSPRTPSSTPSAEADWSPGLELHPDYKTWDPEQVCFFLRRGGGEPALLKNIQENKITGALLPCLDESHFENLGVSSLGERK : 98
Chlorocebus_pygerythrus_agm_ver: --MQQADSDQPSKRLRFDDSPRTPSSTPSAEADWSPGLELHPDYKTWDPEQVCFFLRRGGGEPALLKNIQENKITGALLPCLDESHFENLGVSSLGERK : 98
Cercopithecus_diana_Diana_monkey: --MQQADSDQPSKRPRFDDSPRTPSSTPSAEADGSPGLELHPDHKTWGPEQVCFFLRRGGGEPALLKNIRENKITGALLPCLDESHFENLGVSSLGERK : 98
Cercopithecus_neglectus_deb: --MQQADSDQPSKRPRFDDSPRTPSSTPSAEADGSPGLELHPDYKTWDPEQVCFFLRRGGGEPALLKNIRENKITGALLPCLDESHFENLGVSSLGERK : 98
Macaca_mulatta_mac_1: --MQQADSDQPSKRPRFDDSPRTPSNTPSAEADCFPGLELHPDYKTWGPEQVCFFLRRGGGEPALLKNIRENKITGALLPCLDESHFENLGVSSLGERK : 98
Macaca_mulatta_mac_2: --MQQADSDQPSKRPRFDDSPRTPSNTPSAEADCSPGLELHPDYKTWGPEQVCFFLRRGGGEPALLKNIRENKITGALLPCLDESHFENLGVSSLGERK : 98
Macaca_fascicularis_Crab-eating_macaque: --MQQADSDQPSKRPRFDDSPRTPSNTPSAEADCSPGLELHPDYKTWGPEQVCFFLRRGGGEPALLKNIRENKITGALLPCLDESHFENLGVSSLGERK : 98
Papio_hamadryas_Hamadryas_baboon: --MQQADSDQPSKRPRFDDSPRTPSSTPSAEADWSPGLELHPDYKTWGPEQVCFFLRRGGGEPALLKNIRENKITGALLPCLDESHFENLGVSSLGERK : 98
Mandrillus_sphinx_mnd: --MQQADSDQPSKRPRFDDSPRTPSSTPSAEADCSPGVELHPDYKTWGPEQVCFFLRRGGGEPALLKNIRENKITGALLPCLDESHFENLGVSSLGERK : 98
Cercocebus_atys_sm: --MQQADSDQPSKRPRFDDSPRTPSSTPSAEADCSPGLELHPDYKTWSPEQVCFFLRRGGVEPALLKNIQENKITGALLPCLDESHFENLGVSSLGERK : 98
Cercocebus_torquatus_rcm: --MQQADSDQPSKRPRFDDSPRTPSSTPSAEADCSPGLELHPDYKTWGPEQVCFFLRRGGGEPALLKNIRENKITGALLPCLDESHFENLGVSSLGERK : 98
Cercocebus_chrysogaster_Golden-bellied_mangabey_1: --MQQADSDQPSKRPRFDDSPRTPSSTPSAEADCSPGVELHPDYKTWGPEQVCFFLRRGGGEPALLKNIRENKITGALLPCLDESHFENLGVSSLGERK : 98
Cercocebus_chrysogaster_Golden-bellied_mangabey_2: --MQQADSDQPSKRPRFDDSPRTPSSTPSAEADCSPGLELHPDYKTWGPEQVCFFLRRGGGEPALLKNIRENKITGALLPCLDESHFENLGVSSLGERK : 98
600 610 620
Cebus_apella_Tufted_capuchin: WNYRASAQTPAHLREAPKSRLQLFKDDPV : 627
Callithrix_jacchus_White-tufted-ear_marmoset: WNYRASAQSPARLREASKSRARLFKDDPV : 627
Ateles_geoffroyi_Black-handed_spider_monkey: WNYRASAQSPARLREASKSRVQLFKDDPV : 627
Alouatta_palliata_Mantled_howler_monkey: WNYRASAQSPARLREASKSRVQLFKDDPV : 627
SIVsm Vpx contacts
Hylobates_lar_White-handed_gibbon: WNYGASGQNPTRLREASKSRVQLFKDDPM : 626
Pongo_pygmaeus_Bornean_orangutan: WNDSTSVQNPTRLREASKSRVQLFKDDPM : 626
Pan_troglodytes_cpz: WNDSTSVQNPTRLREASKSRVQLFKDDPM : 626
Homo_sapiens: WNDSTSVQNPTRLREASKSRVQLFKDDPM : 626
Colobus_angolensis_palliatus_Angola_colobus: WNYRTSVQSPTRLREASKSRLQLFKDDPR : 628
Chlorocebus_tantalus_agm_1: WNYRTSVQSPTRLREASKSRLQLFKDDPR : 626
Chlorocebus_tantalus_agm_2: WNYRTSVQSPTRLREASKSRLQLFKDDPR : 626
Chlorocebus_pygerythrus_agm_ver: WNYRTSVQSPTRLREASKSRLQLFKDDPK : 626
Cercopithecus_diana_Diana_monkey: WNYRTSVQSPTRLREASKSRLQLFKDDPR : 626
Cercopithecus_neglectus_deb: WNYRTSVQSPTRLREASKSRLQLFKDDPK : 626
Macaca_mulatta_mac_1: WNYRTSVQSPTRLREASKSRLQLFKDDPK : 626
Macaca_mulatta_mac_2: WNYRTSVQSPTRLREASKSRLQLFKDDPK : 626
Macaca_fascicularis_Crab-eating_macaque: WNYRTSVQSPTRLREASKSRLQLFKDDPK : 626
Papio_hamadryas_Hamadryas_baboon: WNYRTSVQSPTRLREASKSRLQLFKDDPR : 626
Mandrillus_sphinx_mnd: WNYRTSVQSPTRLREASKSRLQLFKDDPK : 626
Cercocebus_atys_sm: WNYRTSVQSPTRLREASKSRLQLFKDDPK : 626
Cercocebus_torquatus_rcm: WNYRTSVQSPTRLREASKSRLQLFKDDPK : 626
Cercocebus_chrysogaster_Golden-bellied_mangabey_1: WNYRTSVQSPTRLREASKSRLQLFKDDPR : 626
Cercocebus_chrysogaster_Golden-bellied_mangabey_2: WNYRTSVQSPTRLREASKSRLQLFKDDPR : 626

SUPPLEMENTAL FIGURE LEGENDS

Figure S1, related to Figure 1. Degron fusion proteins. (**A**) Expression of (NLS)-GFP-degron fusion proteins in was assessed *M. dunni* cells by Western blotting. For each construct the top panel shows an anti-Hsp90 blot loading control. In the lower panels an anti-GFP antibody was used to detect the expression level of each degron fusion indicated. (**B**) Primary FACS data for degron assays. Stable cell lines expressing degrons containing $SAMHD1_{mnd}$ residues 1-114 (upper panels, magenta) or 1-37 (lower panels, purple) were transduced with increasing SIV_{mnd-2} Vpx (left to right) and analysed by flow cytometry.

Figure S2, related to Figure 2. Electron density. Stereo image of ($2F_{obs} – F_c$) refined electron density for DCAF1 (top), Vpx (middle) and SAMHD1 (bottom) contoured at 1 σ. The density is shown as light blue wireframe and the backbone Cα traces of the final refined models as ribbon representation.

Figure S3, related to Figure 2. Vpx conservation of zinc coordination. The protein backbones of (**A**) Vpx from SIV_{mnd-2} (blue) and (**B**) SIV_{smm} (orange) are shown in cartoon representation, α-helices are labelled and zinc ions are shown as grey spheres. Residues that co-ordinate zinc ions are shown as sticks, the coordinating water molecule in SIV_{mnd-2} Vpx as a red sphere and co-ordinating bonds as dashed lines.

Figure S4, related to Figure 3. Superposition of DCAF1-CtD-Vpx complexes. (**A**) Overview of structurally aligned Vpx/DCAF1-CtD complexes. The orientation is as in **Figure 3.** Bound Vpx molecules are shown in cartoon representation, SIV_{mnd-2} Vpx (blue) and SIV_{smm} Vpx

(orange). (**B-E**) Details of Vpx/DCAF1-CtD interactions in the regions boxed in **a**. Residues that make interactions are shown in stick representation, the DCAF1-CtD cartoon is coloured white, in the $SIV_{mnd\text{-}2}$ and grey in the SIV_{smm} complexes.

Figure S5, related to Figure 3 and Figure 6. Multiple sequence alignment of HIV/SIV Vpx and Vpr proteins. 90% type-conserved amino acid residues are highlighted in red, 60% type-conserved in cyan, variable regions are boxed and the position of secondary structure elements is displayed between the Vpr and Vpx groupings. Red stars above the alignment indicate zinc-binding side chains. Residues that interact with SAMHD1 degrons are indicated with blue ($SIV_{mnd\text{-}2}$) or orange (SIV_{smm}) stars (side chain) or dots (backbone). Grey stars indicate Vpx residues with side chains that interact with DCAF1. Darker shading is applied to those that are also type-conserved in Vpr. Numbering below is for $SIV_{mnd\text{-}2}$ 5440 Vpx.

Figure S6, related to Figure 5 and Figure 6. Multiple sequence alignment of primate SAMHD1 N- and C-terminal regions. 100% type-conserved amino acid residues are highlighted in red, 60% type-conserved in cyan. SAMHD1 residues that interact with the respective Vpx are indicated with blue ($SIV_{mnd\text{-}2}$) or orange (SIV_{smm}) stars (side chain) or dots (backbone). Flashes indicate the highly divergent residues 32 and 60.

Table S1A related to Figure 3. Vpx-DCAF1 Interface residues (1595 $Å^2$)

Vpx residue	Region	Interaction	DCAF1 residue	Region
$A2_{MC}$	Nt	HB	R1106	WD40 1
$E3_{MC}$	Nt	HB	R1106	WD40 1
E3	Nt	HB	$R1106_{MC}$	WD40 1
E3	Nt	HB	S1102	WD40 1
E3	Nt	HI	R1106, F1107	WD40 1
A5	Nt	HI	F1107, L1119	WD40 1
P6	Nt	HB	Y1131	WD40 1
P6	Nt	HI	Y1131	WD40 1
P6	Nt	HI	M1166, F1170	WD40 2
$E7_{MC}$	Nt	HB	$S1168_{MC}$	WD40 2
$E7_{MC}$	Nt	HB	$F1170_{MC}$	WD40 2
I28	α1	HI	W1156	WD40 2
E31	α1	HI	W1156	WD40 2
L44	α2	HI	A1377	WD40 7
T47	α2	HI	L1378	WD40 7
C48	α2	HI	L1378	WD40 7
H51	α2	HI	L1378	WD40 7
C52	α2	HI	L1378	WD40 7
Y62	α3	HB	D1092	WD40 7/1
Y65	α3	HB	E1091	WD40 7/1
R66	α3	SB	E1093	WD40 7/1
L68	α3	HI	T1114	WD40 1
L69	α3	HI	C1113, T1114	WD40 1
L70	α3	HI	A1377, L1378, M1380	WD40 7
H72	α3	HB	$N1135_{MC}$	WD40 1/2
H72	α3	HI	C1113, T1114	WD40 1
K73	α3	SB	E1093	WD40 7/1
K73	α3	HB	$S1094_{MC}$	WD40 7/1
K73	α3	HI	C1113	WD40 1
M75	α3	HI	W1156	WD40 2
Y76	α3	HB	$T1097_{MC}$	WD40 1
Y76	α3	HB	$F1355_{MC}$	WD40 7
Y76	α3	HI	A1137, T1139	WD40 1/2
Y76	α3	HI	F1330	WD40 6
T77	α3	HI	P1329, F1330	WD40 6
M79	α3	HI	T1155, W1156	WD40 2
Q81	α3	HB	$Q1314_{MC}$	WD40 6
$Q81_{MC}$	α3	HB	R1225	WD40 4
Q81	α3	HI	L1313, P1329	WD40 6

HB – hydrogen bond, HI – hydrophobic interaction, SB – salt bridge MC – mainchain

Table S1B, related to Figure 3. DCAF1-SAMHD1 interface residues (499 Å^2)

DCAF1 residue	Region	Type of contact	SAMHD1 residue	Region
$A1089_{MC}$	WD40 7/1	HB	R14	Nt
N1090	WD40 7/1	HI	R12	Nt
E1091	WD40 7/1	SB	R12	Nt
D1092	WD40 7/1	SB	R14	Nt
T1114, $T1114_{MC}$	WD40 1	HB	Q8	Nt
Q1116	WD40 1	HI	Q8, R12	Nt
N1132	WD40 1/2	HB	D7, $Q8_{MC}$	Nt
$H1134_{MC}$	WD40 1/2	HB	Q8	Nt
N1135	WD40 1/2	HB	$D5_{MC}$	Nt

HB – hydrogen bond, HI – hydrophobic interaction, SB – salt bridge MC – mainchain

Table S1C, related to Figure 3. Vpx-SAMHD1 Interface residues (1334 Å^2)

Vpx residue	Region	Interaction	SAMHD1 residue	Region
P9	Nt	HI	M1, Q2	Nt
$Q10_{MC}$	Nt	HB	$M1_{MC}$, $Q2_{MC}$	Nt
G11	Nt	HI	Q2	Nt
A12	Nt	HI	Q2	Nt
G13	Nt	HB	D7, S10, $S10_{MC}$	Nt
$E14_{MC}$	Nt	HB	D7	Nt
$V15_{MC}$	Nt	HB	D7	Nt
V15	Nt	HI	D7, P9	Nt
L17	α1	HI	P9, P13	Nt
W20	α1	HI	Q8, P9	Nt
N29	α1	HI	V36	Nt
E39	α2	HB	$P47_{MC}$, $E48_{MC}$, $Q49_{MC}$	SAM domain
L41	α2	HI	V36	Nt
F42	α2	HI	L38, Q49, F52	SAM domain
W45	α2	HI	P34	Nt
N46	α2	HB	E48, $E48_{MC}$	SAM domain
V49	α2	HI	R55	SAM domain
E50	α2	SB	R69	SAM domain
H53	α2	HI	R20	Nt
D54	α2	SB	R20	Nt
D54	α2	SB	R55	SAM domain
H56	α2	HI	F15	Nt
Q57	α2/ α3 loop	HB	R20	Nt
$Q57_{MC}$	α2/ α3 loop	HB	$R20_{MC}$	Nt
R58	α2/ α3 loop	HI	F15	Nt
R58	α2/ α3 loop	HB	$F15_{MC}$	Nt
$S59_{MC}$	α2/ α3 loop	HB	$S18_{MC}$	Nt
Y62	α3	HI	F15, S18	Nt
Y65	α3	HI	P13	Nt
Y65	α3	HB	R12	Nt

HB – hydrogen bond, HI – hydrophobic interaction, SB – salt bridge MC – mainchain

SUPPLEMENTAL EXPERIMENTAL PROCEDURES

Degron assay

Degron reporter constructs comprising two copies of Nuclear Localisation Signal (NLS)-EGFP fused to N-terminal sequences from $SAMHD1_{mnd}$ (NLS-EGFP-$SAMHD1_{mnd}$-NtD) were generated by replacing the human SAMHD1-CtD degron sequence in pCMS28-NLS-EGFP-SAMHD1-CtD (Schwefel et al., 2014) with sequences from the N-terminal region of $SAMHD1_{mnd}$. DNA coding for residues 1-114, 1-37, 37-114, 5-114 or 10-114 was amplified by PCR and inserted into the reporter construct using XhoI/EcoRI restriction sites. Point mutations were created by PCR-based site directed mutagenesis. Virus-like particles (VLPs) were generated by co-transfecting 293T cells with pVSVG, pKB4 and pCMS28-NLS-EGFP-SAMHD1 human-CtD or mandrill-NtD, wildtype or mutant (Schwefel et al., 2014). Stable cell lines were produced by transduction of *Mus dunni* cells followed by puromycin selection. Expression of degron constructs was assessed using Western blotting with anti-EGFP antibodies.

The SIV_{smm} Vpx sequence was amplified by PCR from pIRES2-EGFP-Vpx (a gift from Mario Stevenson), and $SIV_{mnd\text{-}2}$ Vpx was amplified from the PET49 plasmid used for *E. coli* expression. Sequences were inserted into pENTR/D/TOPO (Invitrogen) and transferred into pLgatewayIeYFP (Gateway LR clonase™ II, Invitrogen) to create bicistronic Vpx-IRES-YFP expression constructs. Point mutations were created by PCR-based site directed mutagenesis. VLPs expressing Vpx-IRES-YFP were generated by co-transfecting 293T cells with pVSV-G, pKB4 and pLgatewayIeYFP-Vpx (wildtype or mutants) (Schwefel et al., 2014). Approximately 18 hours after transfection, cells were washed and sodium butyrate medium (0.02 M sodium butyrate, 10% FCS and 1% penicillin/streptomycin in DMEM) was added for 6 hours before

replacing with fresh media. After a further 15 hours VLPs were harvested from the media by filtration.

Parental *Mus dunni* or stable cell lines expressing degron reporters were seeded at 5×10^4 cells per well in a 24-well plate one day prior to infection. Cells were infected with 2-fold serial dilutions of Vpx-YFP VLPs in the presence of 1µg/mL Polybrene. After 48 hours, cells were harvested and the percentage of EGFP-positive and YFP-positive cells was determined by flow cytometry using a FACSVerse analyser (BD Biosciences).

Protein expression and purification

The nucleotide sequences coding for SIV_{mnd-2} Vpx isolate 5440 (Hu et al., 2003) and amino acid residues 1-114 of $SAMHD1_{mnd}$ (Uniprot ID H6WEA4) were synthesised codon-optimised for *E. coli* (Life Technologies). The open reading frames were inserted into pET-49b and pET-52b (Merck Millipore) expression plasmids respectively using flanking XmaI/NotI restriction sites to generate N-terminally GST-tagged and N-terminally Strep-II-tagged fusion proteins. DCAF1-CtD was cloned and expressed as described previously (Schwefel et al., 2014).

SIV_{mnd-2} Vpx and $SAMHD1_{mnd}$-NtD were expressed in the *E. coli* strain Rosetta 2 (DE3) (Merck Millipore). Bacterial cultures were grown in terrific broth medium in a shaking incubator at 37 °C. Protein expression was induced by the addition of 0.1 mM IPTG at A_{600} = 0.5, then further incubated at 18 °C for 20 hours to express recombinant proteins. Cells were harvested by centrifugation for 20 min at 4,500 xg and 4 °C, the cell pellets resuspended in 30 mL lysis buffer (50 mM Tris-HCl pH 7.8, 500 mM NaCl, 4 mM $MgCl_2$, 0.5 mM TCEP, 1x EDTA-free mini complete protease inhibitors (Roche), 0.1 U/ml Benzonase (Novagen) per pellet of 1 L bacteria culture and stored at -20 °C.

SAMHD1$_{mnd}$-NtD cell suspensions were lysed by disruption using an EmulsiFlex-C5 homogeniser (Avestin). The lysate was cleared by centrifugation for 1 hour at 48,000 xg at 4 °C. All further purification steps were performed at 4 °C or on ice. Lysates were applied to 10 mL StrepTactin column (IBA). The column was washed with 600 mL of Wash buffer (50 mM Tris-HCl pH 7.8, 500 mM NaCl, 4 mM $MgCl_2$, 0.5 mM TCEP) and bound proteins were eluted in wash buffer containing 2.5 mM d-desthiobiotin. Eluted fractions were concentrated to 5 mL and further purified on a Superdex75 gel filtration column (GE Healthcare) equilibrated in Gel filtration buffer (10 mM Tris-HCl pH 7.8, 150 mM NaCl, 4 mM $MgCl_2$, 0.5 mM TCEP). Peak fractions containing SAMHD1$_{mnd}$-NtD were pooled, concentrated to 30 mg/mL, snap-frozen in liquid nitrogen in small aliquots and stored at -80 °C.

Protein complex assembly

Cell suspension from 1 L of GST-SIV$_{mnd\text{-}2}$ Vpx was lysed by homogenisation in an EmulsiFlex-C5 (Avestin). The lysate was cleared by centrifugation for 1 h at 48,000 xg at 4 °C. All further purification steps were performed at 4 °C or on ice. 1 mL of glutathione Sepharose (GSH-Sepharose) beads (GE Healthcare) were added to the lysate and incubated for 1 hour on a roller agitator. Beads were pelleted by centrifugation at 4000 xg for 10 min, the supernatant was discarded and the beads washed four times with 50 mL of Wash buffer. For assembly, the GST-SIV$_{mnd\text{-}2}$ Vpx bound beads were resuspended in 10 mL wash buffer in a 15 mL Falcon tube. 1 mg of DCAF1-CtD together with an equimolar amount of SAMHD1$_{mnd}$-NtD and 1 mg of HRV-3C protease (GE Healthcare) were added and the tube was incubated overnight on a rolling agitator. Beads were then removed by centrifugation at 4000 xg for 10 min. The supernatant was concentrated to 5 mL and applied to a Superdex200 size exclusion column equilibrated in Gel

filtration buffer. Peak fractions containing the ternary complex were pooled, concentrated to 12 mg/mL, snap-frozen in liquid nitrogen in small aliquots and stored at -80 °C.

Crystallization and data collection

Crystals of the SIV_{mnd-2} Vpx/$SAMHD1_{mnd}$-NtD/DCAF1CtD complex were grown using the hanging drop vapour diffusion method by mixing 1 μL complex at a concentration of 6.34 mg/mL with 1 μL of reservoir solution containing 0.16 M Trisodium Citrate-HCl pH 5.2 and 4% PEG 6000. Drops were equilibrated over a 450 μL reservoir solution at 18 °C. Crystals were adjusted to 25 % glycerol and cryo-cooled in liquid nitrogen. A data set from a single crystal was collected on station I04 at the Diamond synchrotron light source, UK at a wavelength of 0.97965 Å.

Structure solution

Diffraction data were reduced with the program XDS (Kabsch, 2010). The high resolution cut-offwas based on the $CC_{1/2}$ criteria (Karplus and Diederichs, 2012). The structure was solved by molecular replacement with the program Molrep (Vagin and Teplyakov, 2010) using the previously determined DCAF1-CtD structure and a homology model constructed with the previously determined SIV_{smm} Vpx as template (PDB code 4CC9 (Schwefel et al., 2014)). The SAM domain was placed manually into density using the NMR structure of the human SAMHD1 SAM domain as guidance (PDB code 2E8O). Iterative model adjustment using the program Coot (Emsley et al., 2010) combined with positional, real-space, individual b-factor and TLS refinement with the program phenix.refine (Adams et al., 2010) produced a final model for DCAF1-CtD residues 1073-1315, 1327-1392 (chain A), SIV_{mnd-2} Vpx residues 2-86 (chain B)

and SAMHD1$_{mnd}$-NtD residues 1-22, 34-88, 93-109 (chain C) with R(R$_{free}$)-factors of 17.5% (23.1%). 95.8% of all residues fall in the favoured region of the Ramachandran plot with 0.42% outliers. Data collection and refinement statistics are shown in Table 1.

Multiple sequence alignment

Amino acid sequences were aligned using the ClustalW server and adjusted manually. NCBI accession numbers for Vpr sequences: HIV-1 ETH2220 - U46016, HIV-1 WEAU - U21135, HIV-1 pNL4-3 - AF324493, HIV-1 pNL432 - M28355, SIV$_{smm}$ - AF077017, SIV$_{cpzCAM13}$ - AY169968, SIVcpz$_{LB7}$ - DQ373064, SIV$_{mac239}$ - M33262, SIV$_{mac251}$ - M76764, SIV$_{rcm}$ - HM803689, SIV$_{mnd-2}$ - AF367411, SIV$_{agmSAB1}$ - U04005, SIV$_{agmGRI}$ - M66437, SIV$_{agmVER}$ - KF741091, SIV$_{agmTAN1}$ - U58991; for Vpx sequences: HIV-2A - M30502, HIV-2B - U27200, SIV$_{smm}$ - AF077017, SIV$_{mac251}$ - M76764, SIV$_{mac239}$ - M33262, SIV$_{rcmNG}$ - AF349680, SIV$_{rcmCAM}$ - HM803689, SIV$_{rcmGAB1}$ - AF382829, SIV$_{drl-1}$ - AY159321, SIV$_{mnd-2CM16}$ - AF367411, SIV$_{mnd-25440}$ - AY159322, SIV$_{mnd-2M14}$ - AF328295; for SAMHD1 sequences: *Cebus paella* - JN936910, *Callithrix jacchus* - JN936906, *Ateles geoffroyi* - JN936911, *Alouatta palliata* - JN936912, *Hylobates lar* - JN936889, *Pongo pygmaeus* - JN936888, *Pan troglodytes* - JN936887, *Homo sapiens* - BC036450, *Colobus angolensis* - JN936905, *Chlorocebus tantalus 1* - JN936891, *Chlorocebus tantalus 2* - JN936892, *Chlorocebus pygerythrus* - JQ231137, *Cercopithecus Diana* - JN936902, *Cercopithecus neglectus* - JQ231141, *Macaca mulatta 1* - JN936894, *Macaca mulatta 2* - JN936895, *Macaca fascicularis* - JN936893, *Papio hamadryas* - JN936890, *Mandrillus sphinx* - JN936897, *Cercocebus atys* - JQ231132, *Cercocebus torquatus* - JQ231133, *Cercocebus chrysogaster 1* - JN936898, *Cercocebus chrysogaster 2* - JN936899.

SUPPLEMENTAL REFERENCES

Adams, P.D., Afonine, P.V., Bunkoczi, G., Chen, V.B., Davis, I.W., Echols, N., Headd, J.J., Hung, L.W., Kapral, G.J., Grosse-Kunstleve, R.W., *et al*. (2010). PHENIX: a comprehensive Python-based system for macromolecular structure solution. Acta crystallographica. Section D, Biological crystallography *66*, 213-221.

Emsley, P., Lohkamp, B., Scott, W.G., and Cowtan, K. (2010). Features and development of Coot. Acta crystallographica. Section D, Biological crystallography *66*, 486-501.

Hu, J., Switzer, W.M., Foley, B.T., Robertson, D.L., Goeken, R.M., Korber, B.T., Hirsch, V.M., and Beer, B.E. (2003). Characterization and comparison of recombinant simian immunodeficiency virus from drill (Mandrillus leucophaeus) and mandrill (Mandrillus sphinx) isolates. J Virol *77*, 4867-4880.

Kabsch, W. (2010). Xds. Acta crystallographica. Section D, Biological crystallography *66*, 125-132.

Karplus, P.A., and Diederichs, K. (2012). Linking crystallographic model and data quality. Science *336*, 1030-1033.

Vagin, A., and Teplyakov, A. (2010). Molecular replacement with MOLREP. Acta Crystallographica Section D *66*, 22-25.
